# Zr-Catalyzed Synthesis of Tetrasubstituted 1,3-Diacylpyrroles from *N*-Acyl α-Aminoaldehydes and 1,3-Dicarbonyls

**DOI:** 10.1021/acs.joc.3c00675

**Published:** 2023-06-09

**Authors:** Caria Evans, William J. Berkey, Christopher W. Jones, Stefan France

**Affiliations:** † School of Chemistry and Biochemistry, Georgia Institute of Technology, Atlanta, Georgia 30332, United States; ‡ School of Chemical & Biomolecular Engineering, Georgia Institute of Technology, Atlanta, Georgia 30332, United States; § Renewable Bioproducts Institute, Georgia Institute of Technology, Atlanta, Georgia 30332, United States

## Abstract

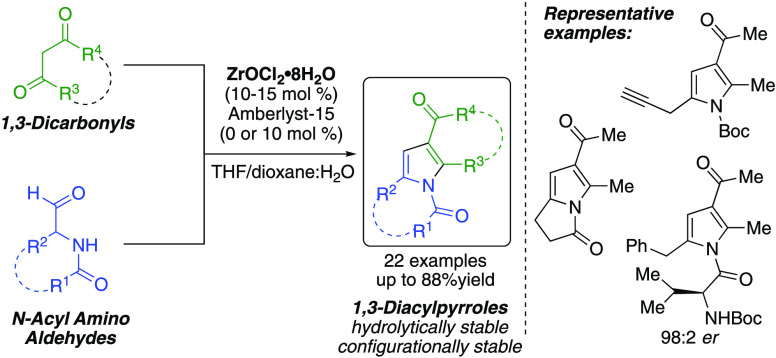

A Zr-catalyzed synthesis of tetrasubstituted 1,3-diacylpyrroles is reported that employs the direct use of *N*-acyl α-aminoaldehydes with 1,3-dicarbonyl compounds. The products were formed in up to 88% yield and shown to be hydrolytically and configurationally stable under the reaction conditions (THF/1,4-dioxane and H_2_O). The *N*-acyl α-aminoaldehydes were readily prepared from the corresponding α-amino acids. The reaction tolerates a wide array of substrate types including alkyl-, aryl-, heteroaryl-, and heteroatom-containing groups on the aminoaldehyde side chain. A variety of 1,3-dicarbonyls proved amenable to the reaction along with an aldehyde derived from a l,l-dipeptide, an aldehyde generated *in situ*, and an *N*-acylated glucosamine.

## Introduction

**Figure 1:**
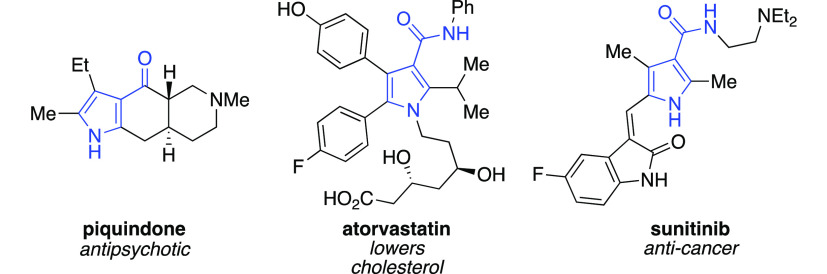
Representative commercial drugs containing the 3-acylpyrrole framework.

Pyrroles constitute an important heterocyclic framework that is heavily represented in biology, natural products, pharmaceuticals, and materials science.^1^ In particular, the 3-acylpyrrole motif is one of the most studied classes of pyrroles and appears in a number of drug molecules and other biologically active derivatives, such as piquindone, atorvastatin, and sunitinib (Figure 1). Based on its therapeutic potential, the 3-acylpyrrole framework has garnered a lot of attention from the synthetic community.^2^ Many approaches have been reported toward their synthesis, including classical ones like the Hantzsch, Knorr, and Paal–Knorr reactions.^3^ Most methods for 3-acylpyrroles involve the use of β-ketocarbonyl compounds, β-enaminones, α-aminoketones, propargylamines, allenamides, and/or α-haloketones.^4^ Despite the wealth of approaches, there is no one size fits all solution as each method comes with its own limitations. Many of these methods require high reaction temperatures (>80 °C) and the use of strong Lewis or Brønsted acids (often in stoichiometric amounts) as well as offer limited scope and functional group compatibility. These caveats necessitate the development of new, milder methods for the synthesis of functionalized 3-acylpyrroles.

**Scheme 1:**
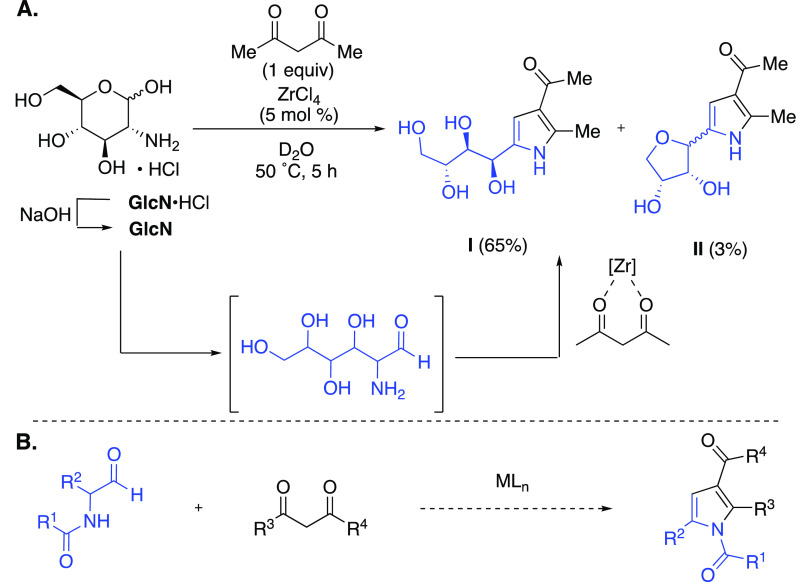
Zr-Catalyzed Reaction of Glucosamine·HCl and Acetylacetone (A) and the Proposed Synthesis of 1,2,3,5-Tetrasubstituted Pyrroles from α-Aminoaldehydes and 1,3-Dicarbonyls (B)

Though various methods are reported toward 3-acylpyrroles, to the best of our knowledge, α-aminoaldehydes^5^ as synthetic precursors have never been directly employed for their synthesis.^6^ This is largely due to their instability and propensity to polymerize or undergo a variety of degradation pathways. For 3-acylpyrrole synthesis, the closest example of the use of an α-aminoaldehyde with a 1,3-dicarbonyl comes from the Garcia Gonzalez reaction^7^ using glucosamine,^8^ which resembles a Knorr pyrrole^9^ synthesis (Scheme 1). As an aldose, glucosamine exists in an equilibrium between its cyclic hemiacetal and acyclic α-aminoaldehyde forms. We recently reported the enhancement of the Garcia Gonzalez reaction with unprotected sugars using Zr(IV)-based Lewis acid catalysis and demonstrated the compatibility of glucosamine to this process.^10^ In the presence of a catalytic ZrCl_4_, a cascade reaction occurs to form a 2-(polyhydroxyalkyl)-substituted 3-acylpyrrole **I** (65%) and a *C*-glycosyl 3-acylpyrrole **II** (3% yield).

Encouraged by this success, we sought to explore the direct reactions of α-aminoaldehydes in the reactions with 1,3-dicarbonyls. Given the instability of unprotected α-aminoaldehydes, we looked to employ bench-stable *N*-acyl derivatives instead. The resulting reactions with 1,3-dicarbonyls would form tetrasubstituted 1,3-diacylpyrroles, which have been identified as potent inhibitors of MEK kinase for the treatment of various cancers.^11^ Thus, herein, we report the first successful synthesis of functionalized 1,3-diacylpyrroles from the direct use of stable α-aminoaldehyde precursors (Scheme 1B).

*N*-Acyl α-aminoaldehydes **1** were mainly prepared from the corresponding *N*-acyl α-amino acids using the one-pot CDI/DIBAL-H method reported by Breinbauer and co-workers.^12^ At the outset, we selected *N*-Boc-phenylalaninal (**1a**, *N*-Boc-Phe-H) as the model system due to its high yield and purity from the acid. Considering our previous work,^10^ acetylacetone **2a** was chosen as the reaction partner. The aldehyde and diketone reaction was stirred in a 0.5 M THF/H_2_O (2:1) solution at room temperature for 6 h (Table 1). With no Lewis acid catalyst present, slight background reactivity was observed with pyrrole **3a** being formed in 8% yield after up to 24 h (entry 1). We next screened Zr(IV) salts that proved successful in our previous work.^10^ When ZrCl_4_ (10 mol %) was employed, pyrrole **3aa** was formed in 62% yield (entry 2). With ZrOCl_2_·8H_2_O (10 mol %), pyrrole **3aa** was generated in 88% yield (entry 3). Comparatively, other Lewis acids did not perform as well, resulting in reduced yields and/or poor conversions (entries 4–10). Increasing the loading of ZrOCl_2_·8H_2_O to 15 mol % (entry 11) resulted in a reduced yield (75%) of pyrrole **3aa** as did reducing the loading to 5 mol % (48%, entry 12). Efforts in changing the ratios of **1a** to **2a** similarly failed to improve yields (entries 13–15).^13^

**Table 1:**
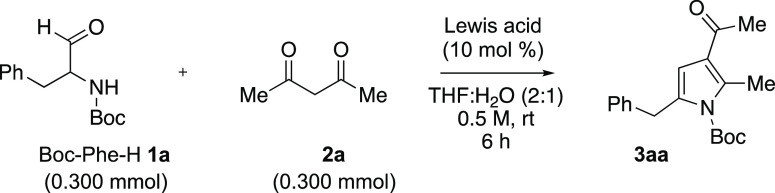
Reaction Optimization^a^

entry	Lewis acid	**1a**:**2a**	yield of **3aa** (%)^b^
1	none	1:1	8^c^
2	ZrCl_4_	1:1	62
3	ZrOCl_2_·8H_2_O	1:1	88
4	Sc(OTf)_3_	1:1	77
5	CeCl_3_	1:1	13
6	Yb(OTf)_3_	1:1	38
7	Bi(OTf)_3_	1:1	46
8	Y(OTf)_3_	1:1	62
9	Ga(OTf)_3_	1:1	33
10	Zn(OTf)_2_	1:1	33
11^d^	ZrOCl_2_·8H_2_O	1:1	75
12^e^	ZrOCl_2_·8H_2_O	1:1	48
13	ZrOCl_2_·8H_2_O	1:5:1	68
14	ZrOCl_2_·8H_2_O	2:1	77
15	ZrOCl_2_·8H_2_O	1:1.5	40

a Reactions performed with Boc-Phe-H **1a** (0.300 mmol), acetylacetone **2a** (0.300 mmol), and Lewis acid (10 mol %) in THF/H_2_O (2:1, 0.6 mL, 0.5 M) at room temperature for 6 h.

b NMR Yield of **3aa** using dimethyl terephthalate as internal standard.

c Yield after 24 h.

d 15 mol % ZrOCl_2_·8H_2_O was employed.

e 5 mol % ZrOCl_2_·8H_2_O was employed.

**Scheme 2:**
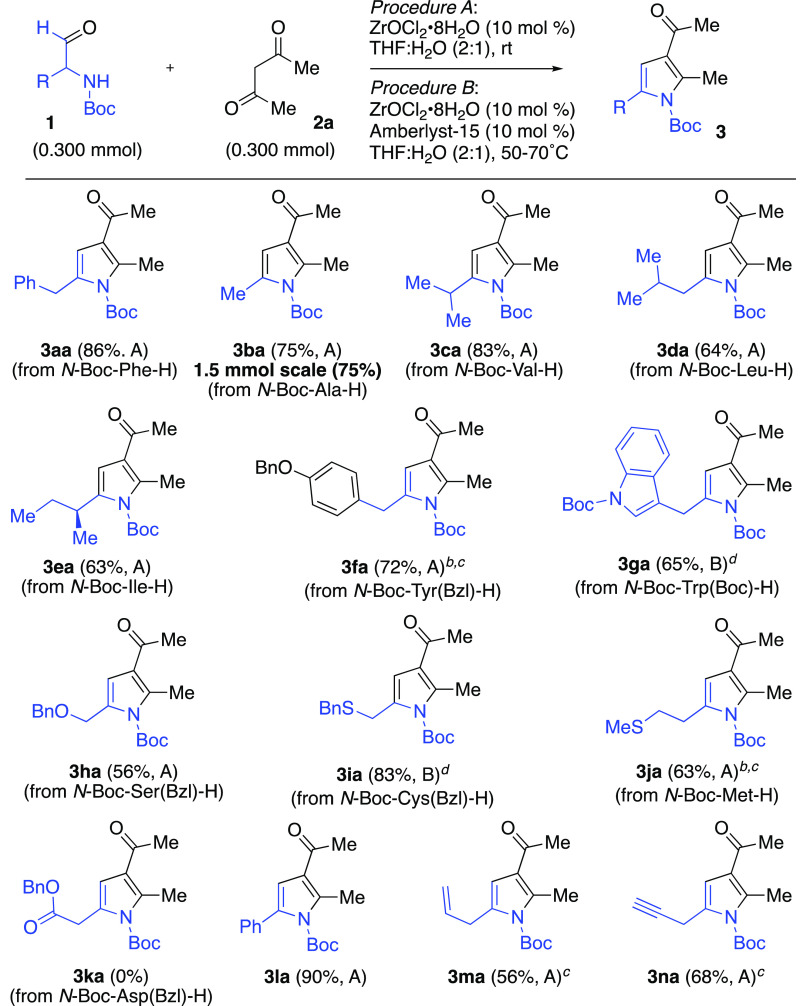
Zr-Catalyzed Reactions of Various α-Aminoaldehydes **1** with Acetylacetone **2a** 15 mol % ZrOCl_2_·*8H_2_O was employed. Reaction was run at 50 °C. Reaction was in 1,4-dioxane:water (2:1). Isolated yields following work-up.

With optimized conditions, we proceeded to explore substrate scope by reacting different *N*-Boc α-aminoaldehydes **1** with acetylacetone **2a** (Scheme 2). α-Alkyl-substituted aminoaldehydes derived from Boc-alaninal (**1b**), Boc-valinal (**1c**), and Boc-leucinal (**1d**), provided their respective pyrroles **3ba**, **3ca**, and **3da** in 78, 83, and 64%, respectively. When the reaction with Boc-alanine (**1b**) was conducted on a 1.5 mmol (∼250 mg) scale, pyrrole **3ba** was obtained in 75% yield. In the case of Boc-isoleucinal (**1e**), the resulting enantiopure pyrrole (*S*)-**3ea** was obtained in 63% yield. For aminoaldehyde **1f** (from *O*-benzyl-*N*-Boc-tyrosinal), the reaction initially gave a 23% yield of pyrrole **3fa**. Fortunately, we determined that running the reaction at 50 °C with 15 mol % catalyst increased the yield of **3fa** to 72% yield. Only 9% yield of pyrrole **3ga** was obtained from the bis-Boc-protected tryptophan-derived aldehyde **1g** under the standard reaction conditions. Given that Brønsted acids have been used cooperatively with Lewis acid to enhance organic reactions,^14^ we looked to the addition of a cocatalyst. Amberlyst-15 ion exchange resin has emerged as an efficient heterogeneous Brønsted acid catalyst that can promote numerous organic reactions (particular ones involving the formation and reaction of enols) while offering synergistic effects with Lewis acids.^15^ After some minor optimization,^16^ we found that conducting the reaction in 1,4-dioxane^17^/H_2_O (2:1) at 50 °C with 10 mol % Amberlyst-15 afforded the desired product **3ga** in 65% yield.

For *O*-benzyl-Boc-serinal (**1h**), the desired pyrrole **3ha** was isolated in 56% yield under standard reaction conditions. The corresponding *S*-benzyl-Boc-cysteinal (**1i**) provided pyrrole **3ia** in poor yield (13%). However, as with the tryptophan-derived aldehyde, we found that heating the reaction at 50 °C in 1,4-dioxane/H_2_O (2:1) with Amberlyst-15 (10 mol %) afforded the desired product **3ia** in 83% yield. For aminoaldehyde **1j** (from Boc-methionine), running the standard reaction at 50 °C with 15 mol % catalyst generated a 63% yield of pyrrole **3ja**. No desired pyrrole product was observed using the aldehyde from *O*-benzyl-*N*-Boc aspartic acid (**1k**).^18^ Lastly, aldehydes derived from Boc-phenylglycine (**1l**), Boc-allylglycine (**1m**), and Bocpropargylglycine (**1m**) smoothly converted to their respective pyrroles **3la**, **3ma**, and **3na** in 90, 56, and 68% yields. Pyrroles **3ma** and **3na** are particularly interesting given the alkene and alkyne moieties that can serve as points of functionalization.

**Scheme 3:**
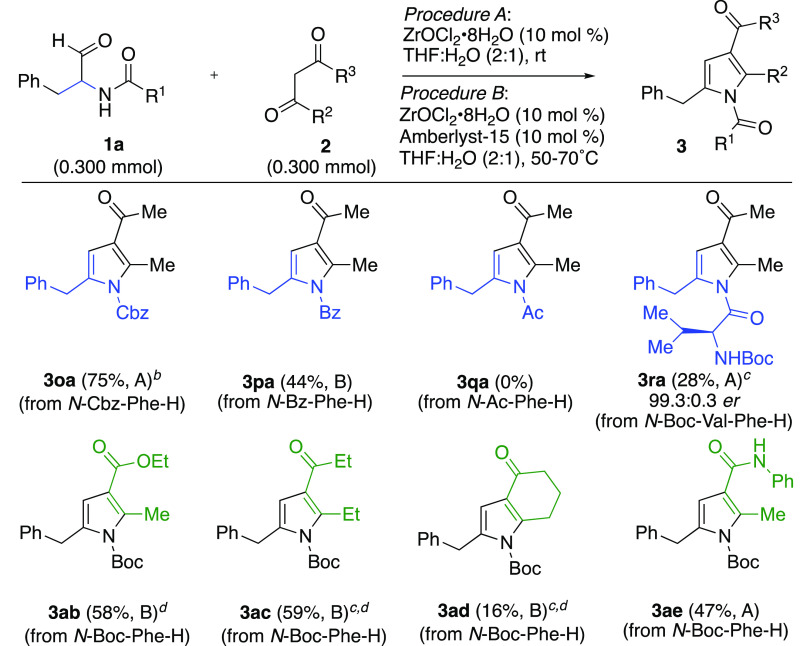
Effects of Changing *N*-Acyl Group or 1,3-Dicarbonyl 30 mol % ZrOCl_2_·*8H_2_O was employed. 15 mol % ZrOCl_2_·*8H_2_O was employed. Performed in THF only (no water). Isolated yields following extraction or column chromatography.

Next, we examined the effects of changing either the *N*-acyl group on the aminoaldehyde or the 1,3-dicarbonyl substrate (Scheme 3). We found that *N*-Cbz-phenylalaninal (**1o**) underperformed compared to its *N*-Boc counterpart (**1a**) with acetylacetone (**2a**), forming pyrrole **3oa** in 67% yield upon conducting the reaction in 1,4-dioxane:H_2_O with 30 mol % catalyst. To convert *N*-benzoyl-phenylalaninal (**1p**), the reaction was heated to 60 °C with added Amberlyst-15 cocatalyst to provide pyrrole **3pa** in 44% yield. In contrast, no desired product was observed with *N*-acetyl phenylalaninal (**1q**). It is possible that the reaction is hindered due to a combination of Lewis acid coordination with the more basic acetyl-protected nitrogen (compared to the carbamates or benzoyl groups) and aminoaldehyde dimerization. Gratifyingly, the l,l-dipeptide aldehyde (**1r**, *N*-Boc-l-Val-l-Phe-H) afforded 28% yield of the desired (*S*)-pyrrole **3ra** with a 99.3:0.7 enantiomeric ratio^19^ (no racemization) when the reaction was performed at 50 °C with 15 mol % ZrOCl_2_·8H_2_O.^20^ This result opens the door for reactions with oligopeptide aldehydes in hopes of providing biologically relevant peptide *N*-acylpyrroles for therapeutic and peptidomimetic applications.^21^

When ethyl acetoacetate (**2b**) was used in place of acetylacetone, pyrrole **3ab** was formed in 58% yield when the reaction was conducted in THF (no added H_2_O) at 50 °C with added Amberlyst-15. Ester hydrolysis was observed when water was present. Hepta-3,5-dione (**2c**) required 15 mol % ZrOCl_2_·8H_2_O in conjunction with Amberlyst-15 at 50 °C to form pyrrole **3ac** in 59% yield. Cyclohexane-1,3-dione (**2d**) afforded a particularly low yield (16%) of tetrahydroindol-4-one **3ad** using similar conditions at 70 °C. This is likely due to the reduced nucleophilicity of **2d** (resulting from the reduced p*K*_a_ and added stability of the enol form in aqueous conditions compared to the acyclic diketones).^22^ Lastly, 3-oxo-*N*-phenylbutanamide (**2e**) provided pyrrole **3ae** in 47% yield using the standard reaction conditions.

**Scheme 4:**
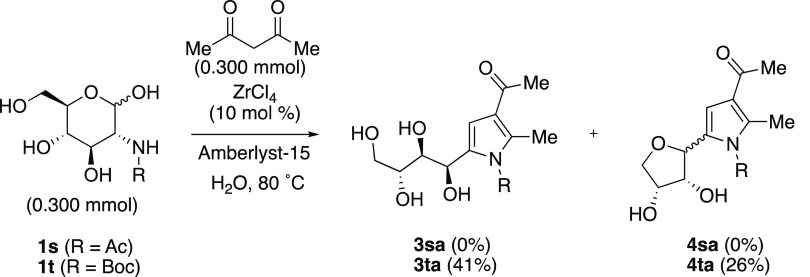
Reactions of *N*-Acyl-glucosamines **1s** and **1t** with Acetylacetone (**2a**)

*N*-Acyl glucosamines were subsequently studied as substrates for the reaction (Scheme 4). We started with *N*-acetyl glucosamine (**1s**) as a model given its importance in biology, its role as the key monomer in chitin, and its general availability.^23^ Unfortunately, in line with what we observed with *N*-acetyl phenylalaninal (**1q**), we were unable to generate any desired pyrrole from **1s** using the original or any of our modified reaction conditions. It is not surprising as its cyclic form is uniquely stabilized over its acyclic form as determined by the kinetics of mutarotation.^24^ Fortunately, when acetylacetone (**2a**) and *N*-Boc-glucosamine (**1t**) were used in combination with ZrCl_4_ and Amberlyst-15 at 80 °C in H_2_O, a 1.56:1 mixture of polyhydroxyalkyl pyrrole **3ta** and *C*-glycosylpyrrole **4ta** was obtained in a 67% total yield.

**Scheme 5:**
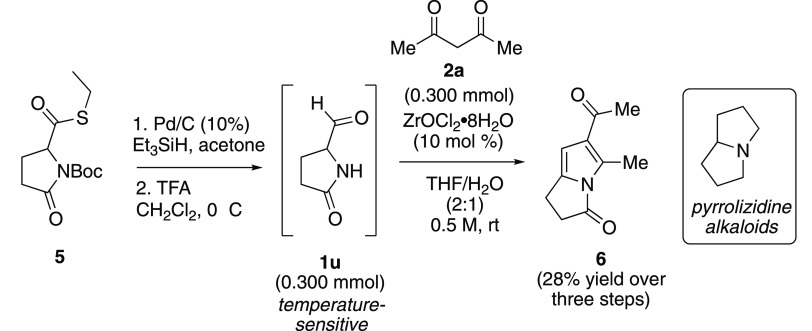
Reaction of 5-Oxoprolinal (**1u**) with Acetylacetone (**2a**)

Finally, we were interested in examining the reaction of aminoaldehydes that could not be readily prepared by the Briebauer method due to stability issues. For example, only trace amounts of 5-oxoprolinal (**1u**) could be detected from the corresponding acid using CDI/DIBAL-H. 5-Oxoprolinal (**1u**) has been previously prepared *in situ* from *N*-Boc-protected thioester **5**.^25^ Crude **1u** was then used immediately for further reactions since it readily degraded. Following the known synthetic sequence, thioester **5** was first reduced to the aldehyde and then deprotected using trifluoroacetic acid (TFA) to give crude **1u**. Immediate treatment with acetylacetone and ZrOCl_2_·8H_2_O under the reaction conditions provided pyrrolizidin-3-one **6** in 28% yield over three steps from thioester **5** without the need for column chromatography. Pyrrole **6** is representative of the pyrrolizidine-3-one scaffold^26^ that is present in a variety of bioactive natural products, drug candidates, herbicides, and fungicides (Scheme 5).

**Scheme 6:**
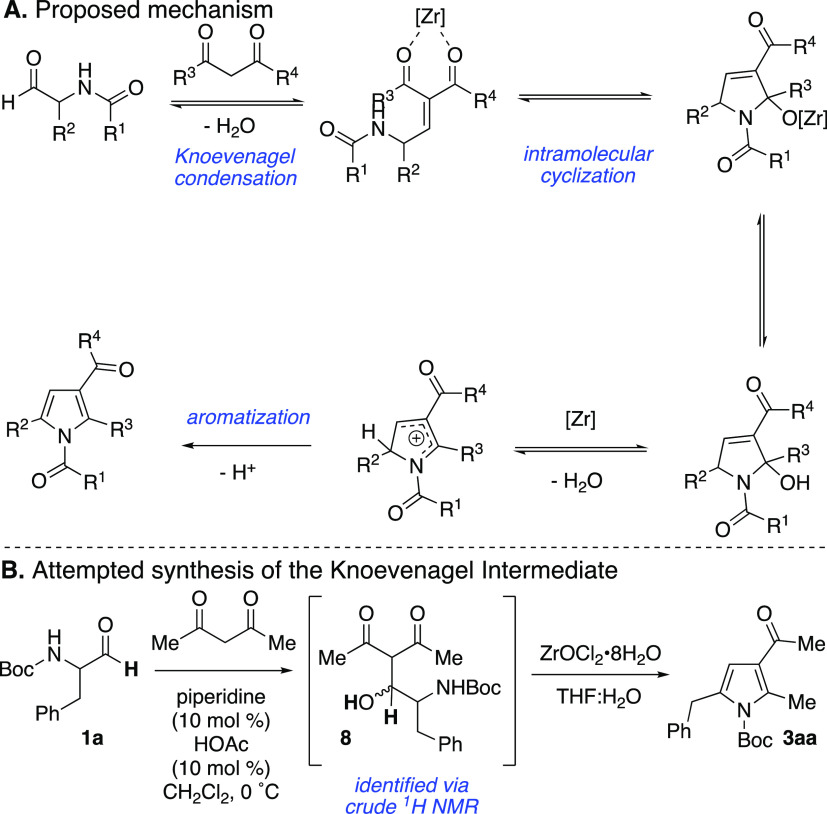
(A) Proposed Mechanism for Zr-Catalyzed Reaction with *N*-Acyl α-Aminoaldehydes and (B) Attempted Knoevenagel Intermediate Generation and Reactivity

Mechanistically, we posit that the first step of the pyrrole-forming reaction with the *N*-acyl aminoaldehyde involves Zr-catalyzed Knoevenagel condensation followed by intramolecular attack of the amide moiety onto the adjacent Zr-coordinated ketone (from the 1,3-dicarbonyl, Scheme 6A).^10^ Subsequently, a series of proton transfers and loss of water provide the *N*-acylpyrrole. This mechanism supports the observation that the nature of the *N*-acyl group plays an important and direct role in product outcomes. To support this mechanism, we looked to prepare the Knoevenagel product and subject it to the reaction conditions. Upon subjecting Boc-Phe-H **1a** and acetylacetone **2a** to catalytic piperidine and HOAc, Knoevaenagel product was not observed, but a diastereomeric mixture of aldol addition product **8** was identified by crude ^1^H NMR. Furthermore, both pyrrole **3aa** and unreacted aldehyde **1a** were not detected. As anticipated, treatment of the crude mixture with ZrOCl_2_·8H_2_O under the standard reaction conditions generated the desired pyrrole **3aa**. Interestingly, attempts to purify the crude aldol addition product using silica gel also resulted in some conversion to pyrrole.

**Scheme 7:**
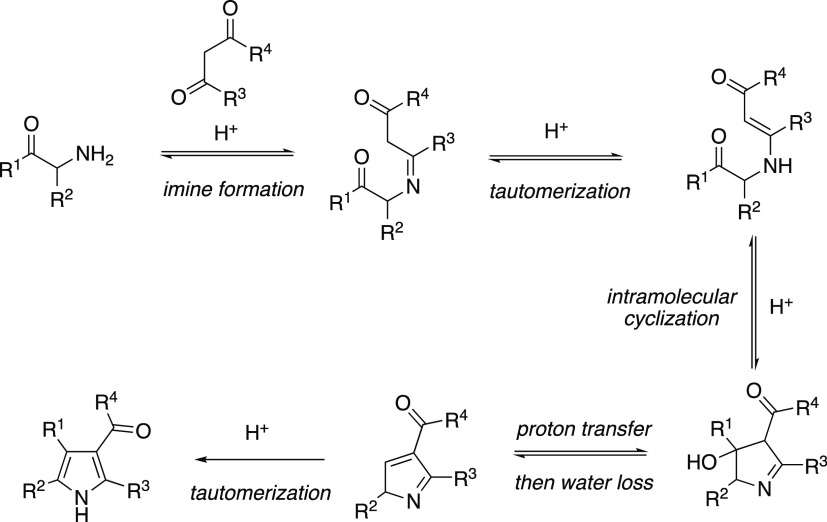
Accepted Knorr Reaction Mechanism

This proposed mechanism differs from the classic Knorr pyrrole synthesis using unprotected aminoketones with 1,3-dicarbonyls (Scheme 7).^27^ The first step of the Knorr reaction involves imine formation between the amino moiety and the ketone of the 1,3-dicarbonyl. Subsequent tautomerization to the enamine sets up the intramolecular cyclization onto the adjacent ketone. Pyrrole is formed following sequential proton transfer, loss of water, and tautomerization.

In summary, we report the first direct use of *N*-protected α-aminoaldehydes with 1,3-dicarbonyls to generate 1,3-diacylpyrrole compounds using a Zr(IV) catalyst and, in certain cases, Amberlyst-15 as a cocatalyst. *N*-Acyl α-aminoaldehydes were accessible directly from commercial or readily accessible *N*-acyl α-amino acids, including ones containing purely alkyl side chains, heteroatoms, and (hetero)aryl groups. The resulting pyrroles were formed in up to 88% yield. In most examples, the pyrroles were obtained in high purity (>99%) without the need for column chromatography following extraction. The 1,3-dicarbonyl component could also be varied as needed to provide broader scope. An *N*-protected l,l-dipeptide aldehyde also proved amenable to the reaction conditions (providing the highly enantioenriched pyrrole) along with an example of an *N*-acyl aminoaldehyde that was prepared *in situ* to form a functionalized pyrrolizidin-3-one. The method provides functionalized *N*-acylpyrroles that are configurationally and hydrolytically stable in acidic aqueous solution. This is significant as *N*-acylpyrroles have historically not been employed in medicinal chemistry due to issues with hydrolysis.^28^ Thus, future efforts will involve exploring the medicinal chemistry of the synthesized 1,3-diacylpyrroles.

## Experimental Section

### General Information

Chromatographic purification was performed as flash chromatography with Silicycle SiliaFlash P60 silica gel (40–63 μm) or preparative thin-layer chromatography (prep-TLC) using silica gel F254 (1000 μm) plates and solvents indicated as eluent with 0.1–0.5 bar pressure. For quantitative flash chromatography, technical grade solvents were utilized. Analytical thin-layer chromatography (TLC) was performed on Silicycle SiliaPlate TLC silica gel F254 (250 μm) TLC glass plates. Visualization was accomplished with UV light. Infrared (IR) spectra were obtained via attenuated total reflection (ATR) with a diamond plate using a Nicolet 6700 Fourier transform infrared spectrophotometer. The IR bands are characterized as broad (br), weak (w), medium (m), and strong (s). Proton and carbon nuclear magnetic resonance spectra (^1^H NMR and ^13^C NMR) were recorded on a Varian Mercury Vx 300 MHz spectrometer or a Bruker AV3 HD 700 MHz spectrometer with solvent resonances as the internal standard (^1^H NMR: CDCl_3_ at 7.26 ppm; ^13^C NMR: CDCl_3_ at 77.0 ppm). ^1^H NMR data are reported as follows: chemical shift (ppm), multiplicity (s = singlet, d = doublet, dd = doublet of doublets, dt = doublet of triplets, ddd = doublet of doublet of doublets, t = triplet, m = multiplet, brs = broad singlet), coupling constants (Hz), and integration. Samples were analyzed by flow injection ESI in positive mode using a Thermo Scientific Vanquish Horizon coupled to a Thermo Scientific QEHF mass spectrometer. The mobile phase was 90/10 isopropanol:acetonitrile containing 0.1% formic acid and 10 mM ammonium formate at a rate of 0.05 mL/min. Samples were injected at 1 μL. The mass spectrometer was operated at 132–1000 mz and a resolution of 240,000. Melting points were determined using an Electrothermal digital melting point apparatus, Melting Point Apparatus, AP7431, and are uncorrected. Reagents were purchased from various commercial suppliers, stored as specified by the manufacturer, and used without purification unless otherwise stated.

### General Synthesis of *N*-Acyl α-Aminoaldehydes

*N*-Acyl α-aminoaldehydes were synthesized following the previously reported literature method.^12^ Commercially bought *N*-acyl amino acids were first converted to the acyl imidazolides using 1,1′-carbonyldiimidazole (1.1 equiv) and later reduced to the corresponding *N*-acyl α-aminoaldehydes using DIBAL-H (1.0 M in CH_2_Cl_2_) (2.1 equiv). Extractive work-up resulted in pure products with isolated yields of 45–90%.

#### *tert*-Butyl (*S*)-(1-Oxo-3-phenylpropan-2-yl)carbamate (**1a**)

Prepared following the general procedure using commercially available Boc-Phe-OH (1.50 g, 5.70 mmol), which was dissolved in CH_2_Cl_2_ (40 mL). The colorless solution was treated with CDI (1.02 g, 6.27 mmol) at 0 °C for 60 min, and subsequently, dropwise with DIBAL-H (11.1 mL, 11.1 mmol). The mixture was stirred for 60 min at −78 °C. Extractive work-up afforded compound **1a** as a white powder (1.28 g, 90% yield). ^**1**^**H NMR** (700 MHz, CDCl_3_) δ 9.63 (s, 1H, CHO), 7.32–7.15 (m, 5H, Ar–H), 5.06 (m, 1H, NH), 4.41 (m, 1H, CHCHO), 3.12–3.13 (d, *J* = 6.4 Hz, 2H, CH_2_), 1.43 (s, 9H, (CH_3_)_3_CO). ^**13**^**C**{^1^H} **NMR** (175 MHz, CDCl_3_) δ 199.5, 155, 135.8, 129.4, 128.8, 127.1, 80.2, 60.8, 35.5, 28.6 ppm. All spectroscopic data were consistent with those previously reported.^12^

#### *tert*-Butyl (*S*)-(1-Oxopropan-2-yl)carbamate (**1b**)

Prepared following the general procedure using commercially available Boc-Ala-OH (2.36 g, 12.5 mmol), which was dissolved in CH_2_Cl_2_ (88 mL). The colorless solution was treated with CDI (2.23 g, 13.8 mmol) at 0 °C for 60 min, and subsequently, dropwise with DIBAL-H (26.3 mL, 26.3 mmol). The mixture was stirred for 60 min at −78 °C. Extractive work-up afforded compound **1b** as a white powder (2.00 g, 92% yield). ^**1**^**H NMR** (700 MHz, CDCl_3_) δ 9.56 (s, 1H, CHO), 5.11 (s, 1H, NH), 4.22 (s, 1H, CHCHO), 1.47 (s, 9H, (CH_3_)_3_CO) 1.33 (d, ^3^*J* = 7.3 Hz, 3H, CHCH_3_) ppm. ^**13**^**C**{^1^H} **NMR** (175 MHz, CDCl_3_) δ 199.7, 155.3, 80.1, 55.5, 28.4, 15.1 ppm. All spectroscopic data were consistent with those previously reported.^12^

#### *tert*-Butyl (*S*)-(3-Methyl-1-oxobutan-2-yl)carbamate (**1c**)

Prepared following the general procedure using commercially available Boc-Val-OH (1.50 g, 6.9 mmol), which was dissolved in CH_2_Cl_2_ (48 mL). The colorless solution was treated with CDI (1.23 g, 7.59 mmol) at 0 °C for 60 min, and subsequently, dropwise with DIBAL-H (14.5 mL, 14.5 mmol). The mixture was stirred for 60 min at −78 °C. Extractive work-up afforded compound **1c** as a colorless oil (1.21 g, 87% yield). ^**1**^**H NMR** (700 MHz, CDCl_3_) δ 9.65 (s, 1H, CHO), 5.09 (m, 1H, NH), 4.27–4.24 (m, 1H, CHCHO), 2.31–2.26 (m, 1H, CH(CH_3_)_2_), 1.45 (s, 9H, (CH_3_)_3_CO), 1.04 (d, 3H, ^3^*J* = 6.9 Hz, CH(CH_3_)(CH_3_)), 0.95 (d, 3H, ^3^*J* =7.0 Hz, CH(CH_3_)(CH_3_)) ppm. ^**13**^**C**{^1^H} **NMR** (175 MHz, CDCl_3_) δ 200.4, 155.6, 80.0, 64.4, 29.0, 28.2, 19.0, 17.4 ppm. All spectroscopic data were consistent with those previously reported.^12^

#### *tert*-Butyl (*S*)-(4-Methyl-1-oxopentan-2-yl)carbamate (**1d**)

Prepared following the general procedure using commercially available Boc-Leu-OH (1.50 g, 6.22 mmol), which was dissolved in CH_2_Cl_2_ (44 mL). The colorless solution was treated with CDI (1.07 g, 6.62 mmol) at 0 °C for 60 min, and subsequently, dropwise with DIBAL-H (12.6 mL, 12.6 mmol). The mixture was stirred for 60 min at −78 °C. Extractive work-up afforded compound **1d** as a colorless oil (1.04 g, 75% yield). 1H NMR (700 MHz, CDCl_3_) δ 9.58 (s, 1H, CHO), 4.93 (s, 1H, NH), 4.25 (m, 1H, CHCHO), 1.80–1.73 (m, 1H, (CH_3_)_2_CH), 1.67–1.60 (m, 2H, NCHCHH), 1.44 (s, 9H, (CH_3_)_3_CO), 0.97–0.95 (m, 6H, 2CH_3_) ppm. ^**13**^**C**{^1^H} **NMR** (175 MHz, CDCl_3_) δ 200.4, 155.6 pp, 80.0, 58.2, 37.8, 28.2, 24.4, 23.2, 21.7 ppm. All spectroscopic data were consistent with those previously reported.^12^

#### *tert*-Butyl ((2*S*,3*S*)-3-Methyl-1-oxopentan-2-yl)carbamate (**1e**)

Prepared following the general procedure using commercially available Boc-Ile-OH (1.50 g, 6.5 mmol), which was dissolved in CH_2_Cl_2_ (46 mL). The colorless solution was treated with CDI (1.16 g, 7.15 mmol) at 0 °C for 60 min, and subsequently, dropwise with DIBAL-H (13.7 mL, 13.7 mmol). The mixture was stirred for 60 min at −78 °C. Extractive work-up afforded compound **1e** as a colorless oil (0.840 g, 62% yield). ^**1**^**H NMR** (700 MHz, CDCl_3_) δ 9.65 (s, 1H, CHO), 5.13–5.12 (m, 1H, NH), 4.30–4.27 (m, 1H, CHCHO), 2.03 (m, 1H, C*H*CH_2_), 1.48–1.47 (m, 1H, CH_3_CHH), 1.44 (s, 9H, (CH_3_)_3_CO), 1.31–1.28 (m, 1H, CH_3_CHH), 0.98–0.95 (m, 6H, 2CH_3_) ppm. ^**13**^**C**{^1^H} **NMR** (175 MHz, CDCl_3_) δ = 200.6, 155.7, 79.9, 64.2, 36.4, 28.3, 25.3, 15.6, 11.9 ppm. All spectroscopic data were consistent with those previously reported.^12^

#### *tert*-Butyl (*S*)-(1-(4-(Benzyloxy)phenyl)-3-oxopropan-2-yl)carbamate (**1f**)

Prepared following the general procedure using commercially available Boc-Tyr(OBzl)-OH (1.50 g, 4.04 mmol), which was dissolved in CH_2_Cl_2_ (28 mL). The colorless solution was treated with CDI (0.720 g, 4.44 mmol) at 0 °C for 60 min, and subsequently, dropwise with DIBAL-H (8.48 mL, 8.48 mmol). The mixture was stirred for 60 min at −78 °C. Extractive work-up afforded compound **1f** as a white powder (1.35 g, 93% yield). ^**1**^**H NMR** (700 MHz, CDCl_3_) δ 9.62 (s, 1H, CHO), 7.44–7.35 (m, 5H, Ar–H), 7.09–7.07 (m, 2H, Ar-H), 6.93–6.91 (m, 2H, Ar-H), 5.04 (s, 2H, OCH_2_Ph), 4.42–4.37 (m, 1H, CHNH), 3.07–3.05 (d, ^3^*J* = 6.2 Hz, 2H, CH_2_Ar), 1.44 (s, 9H, (CH_3_)_3_CO) ppm. ^**13**^**C**{^1^H} **NMR** (175 MHz, CDCl_3_) δ 199.6, 155.4 ppm, 157.9, 136.9, 130.4, 128.6, 128.0, 127.5, 115.1, 80.2, 70.1, 34.6, 28.0 ppm. All spectroscopic data were consistent with those previously reported.^29^

#### *tert*-Butyl 3-(2-((*tert*-Butoxycarbonyl)amino)-3-oxopropyl)-1*H*-indole-1-carboxylate (**1g**)

Prepared following the general procedure using commercially available Boc-Trp (Boc)-OH (1.50 g, 3.71 mmol), which was dissolved in CH_2_Cl_2_ (26 mL). The colorless solution was treated with CDI (0.661 g, 4.08 mmol) at 0 °C for 60 min, and subsequently, dropwise with DIBAL-H (7.79 mL, 7.79 mmol). The mixture was stirred for 60 min at −78 °C. Extractive work-up afforded compound **1g** as a clear oil (1.35 g, 94% yield). ^**1**^**H NMR** (700 MHz, CDCl_3_) δ 9.66 (s, 1H, CHO), 8.13 (s, 1H, IndH), 7.56–7.55 (d, ^3^*J* = 7.7 Hz, 1H, Ar-H), 7.43 (s, 1H, Ar-H), 7.34–7.33 (m, 1H, Ar-H), 7.26–7.25 (m, 1H, Ar-H), 5.20 (s, 1H, NH), 4.50 (s, 1H, CHNH), 3.24–3.22 (t, ^3^*J* = 7.2 Hz, 2H, CH_2_), 1.66 (s, 9H, (CH_3_)_3_CO), 1.45 (s, 9H, (CH_3_)_3_CO) ppm. ^**13**^**C**{^1^H} **NMR** (175 MHz, CDCl_3_) δ 199.5, 155.4, 149.5, 135.5, 130.3, 124.7, 124.2, 122.8, 119.0, 115.3, 114.8, 83.8, 80.3, 28.2, 25.1 ppm. All spectroscopic data were consistent with those previously reported.^30^

#### *tert*-Butyl (*S*)-(1-(Benzyloxy)-3-oxopropan-2-yl)carbamate (**1h**)

Prepared following the general procedure using commercially available Boc-Ser-(OBzl)-OH (1.50 g, 8.6 mmol), which was dissolved in CH_2_Cl_2_ (60 mL). The colorless solution was treated with CDI (1.53 g, 9.46 mmol) at 0 °C for 60 min, and subsequently, dropwise with DIBAL-H (18.1 mL, 18.1 mmol). The mixture was stirred for 60 min at −78 °C. Extractive work-up afforded compound **1h** as a colorless oil (1.24 g, 87% yield). ^**1**^**H NMR** (700 MHz, CDCl_3_) δ 9.65 (s, 1H, CHO), 7.38–7.27 (m, 5H, Ar–H), 5.45 (m, 1H, NH), 4.55–4.50 (q, ^3^*J* = 12.0 Hz, 2H, CH_2_), 4.34 (s, 1H, CH), 4.02 (d, ^3^*J* = 9.2 Hz, 1H, CH_2_), 3.71 (m, 1H, CH_2_), 1.48 (s, 9H, (CH_3_)_3_CO) ppm. ^**13**^**C**{^1^H} **NMR** (175 MHz, CDCl_3_) δ 199.2, 155.6 ppm, 137.3, 128.6, 128.0, 127.7, 80.3, 73.6, 67.8, 36.4, 28.3 ppm. All spectroscopic data were consistent with those previously reported.^31^

#### *tert*-Butyl (*R*)-(1-(Benzylthio)-3-oxopropan-2-yl)carbamate (**1i**)

Prepared following the general procedure using commercially available Boc-Cys(OBzl)-OH (1.50 g, 4.82 mmol), which was dissolved in CH_2_Cl_2_ (34 mL). The colorless solution was treated with CDI (0.843 g, 5.30 mmol) at 0 °C for 60 min, and subsequently, dropwise with DIBAL-H (10.1 mL, 10.1 mmol). The mixture was stirred for 60 min at −78 °C. Extractive work-up afforded compound **1i** as a white powder (0.87 g, 61% yield). ^**1**^**H NMR** (700 MHz, CDCl_3_) δ 9.53 (s, 1H, CHO), 7.33–7.30 (m, 5H, Ar–H), 5.33 (s, 1 H, NH), 4.33–4.28 (m, 1H, CHNH), 3.74 (s, 2H, CH_2_S), 2.87–2.84 (m, 2H, CH_2_S), 1.46 (m, 9H, (CH_3_)_3_CO) ppm. ^**13**^**C**{^1^H} **NMR** (175 MHz, CDCl_3_) δ 198.7, 155.4 ppm, 157.9, 137.5, 128.9, 128.7, 127.4, 115.1, 80.5, 59.1, 36.9, 28.3 ppm. All spectroscopic data were consistent with those previously reported.^32^

#### *tert*-Butyl (*S*)-(4-(Methylthio)-1-oxobutan-2-yl)carbamate (**1j**)

Prepared following the general procedure using commercially available Boc-Met-OH (1.50 g, 6.02 mmol), which was dissolved in CH_2_Cl_2_ (43 mL). The colorless solution was treated with CDI (1.07 g, 6.61 mmol) at 0 °C for 60 min, and subsequently, dropwise with DIBAL-H (12.6 mL, 12.6 mmol). The mixture was stirred for 60 min at −78 °C. Extractive work-up afforded compound **1j** as a white powder (1.15 g, 82% yield). ^**1**^**H NMR** (700 MHz, CDCl_3_) δ 9.63 (s, 1H, CHO), 5.22 (s, 1H, NH), 4.31–4.28 (m, 1H, C*H*NH), 2.59–2.54 (m, 2H, C*H*_2_S), 2.24–2.21 (m, 1H, C*H*HCH_2_S), 2.07 (s, 3H, SCH_3_), 1.93–1.90 (m, 1H, CH*H*CH_2_S), 1.44 (m, 9H, (CH_3_)_3_CO) ppm. ^**13**^**C**{^1^H} **NMR** (175 MHz, CDCl_3_) δ 199.1, 155.5, 80.3, 59.0, 29.8, 28.7, 28.3, 15.4 ppm. All spectroscopic data were consistent with those previously reported.^12^

#### Benzyl 3-((*tert*-Butoxycarbonyl)amino)-4-oxobutanoate (**1k**)

Prepared following the general procedure using commercially available Boc-Asp(Bzl)-OH (1.50 g, 4.64 mmol), which was dissolved in CH_2_Cl_2_ (33 mL). The colorless solution was treated with CDI (0.827 g, 5.10 mmol) at 0 °C for 60 min, and subsequently, dropwise with DIBAL-H (9.3 mL, 9.28 mmol). The mixture was stirred for 60 min at −78 °C. Extractive work-up afforded compound **1j** as a white powder (0.820 g, 57% yield). ^**1**^**H NMR** (700 MHz, CDCl_3_) δ 9.73 (s, 1H, CHO), 7.36 (m, 5H, Ar-H), 5.54 (m, 1H, NH), 5.18 (d, 2H, C*H*_2_Ph), 4.64 (m, 1H, CHNH), 3.06 (m, 1H, C*H*HC(O)), 2.82 (m, 1H, CH*H*C(O)), 1.44 (m, 9H, (CH_3_)_3_CO) ppm. ^**13**^**C**{^1^H} **NMR** (175 MHz, CDCl_3_) δ 199.4, 171.2, 155.4, 135.5, 128.6, 128.5, 128.4, 80.2, 50.1, 36.9, 167.5, 28.4, 28.3, 14.2 ppm. All spectroscopic data were consistent with those previously reported.^12^

#### *tert*-Butyl (2-Oxo-1-phenylethyl)carbamate (**1l**)

Prepared following the general procedure using commercially available Boc-PhG-OH (0.100 g, 0.398 mmol), which was dissolved in CH_2_Cl_2_ (3 mL). The colorless solution was treated with CDI (0.071 g, 0.438 mmol) at 0 °C for 60 min, and subsequently, dropwise with DIBAL-H (0.836 mL, 0.836 mmol). The mixture was stirred for 60 min at −78 °C. Extractive work-up afforded compound **1l** as a yellow oil (0.041 g, 43% yield). ^**1**^**H NMR** (700 MHz, CDCl_3_) δ 9.57 (s, 1H, CHO), 7.44–7.27 (m, 5H, Ar–H), 5.79 (m, 1H, NH), 5.35 (m, 1H, CHCHO), 1.46 (s, 9H, (CH_3_)_3_CO) ppm. ^**13**^**C**{^1^H} **NMR** (175 MHz, CDCl_3_) δ 195.5, 155.0, 133.8, 129.4, 128.8, 127.9, 80.2, 65.2, 28.6 ppm. All spectroscopic data were consistent with those previously reported.^12^

#### *tert*-Butyl (1-Oxopent-4-en-2-yl)carbamate (**1m**)

Prepared following the general procedure using commercially available Boc-Allyl-Gly-OH (0.250 g, 1.16 mmol), which was dissolved in CH_2_Cl_2_ (8 mL). The colorless solution was treated with CDI (0.208 g, 1.28 mmol) at 0 °C for 60 min, and subsequently, dropwise with DIBAL-H (1.40 mL, 1.40 mmol). The mixture was stirred for 60 min at −78 °C. Extractive work-up afforded compound **1m** as a clear oil (0.150 g, 65% yield). ^**1**^**H NMR** (700 MHz, CDCl_3_) δ 9.60 (s, 1H, CHO), 5.72–5.69 (m, 1H, NH), 5.18–5.16 (m, 2H, =CH_2_), 5.14–5.10 (m, 1H, =CH), 4.29–4.26 (m, 1H, CHNH), 2.61–2.58 (m, 1H, CH_2_), 2.52–2.48 (m, 1H, CH_2_), 1.45 (s, 9H, (CH_3_)_3_CO) ppm. ^**13**^**C**{^1^H} **NMR** (175 MHz, CDCl_3_) δ 199.5, 155.1, 132.0, 119.3, 80.1, 60.2, 33.7, 28.3 ppm. All spectroscopic data were consistent with those previously reported.^33^

#### *tert*-Butyl (1-Oxopent-4-*yn*-2-yl)carbamate (**1n**)

Prepared following the general procedure using commercially available Boc-Propargyl-Gly-OH (0.250 g, 1.16 mmol), which was dissolved in CH_2_Cl_2_ (8 mL). The colorless solution was treated with CDI (0.208 g, 1.28 mmol) at 0 °C for 60 min, and subsequently, dropwise with DIBAL-H (1.40 mL, 1.40 mmol). The mixture was stirred for 60 min at −78 °C. Extractive work-up afforded compound **1n** as a clear oil (0.100 g, 44% yield). ^**1**^**H NMR** (700 MHz, CDCl_3_) δ 9.64 (s, 1H, CHO), 5.38 (s, 1H, NH), 4.33–4.30 (m, 1H, CHNH), 2.84–2.78 (m, 1H, CH), 2.70–2.65 (m, 2H, CH_2_), 1.45 (s, 9H, (CH_3_)_3_CO) ppm. ^**13**^**C**{^1^H} **NMR** (700 MHz, CDCl_3_) δ 198.2, 155.4, 80.6, 78.5, 72.0, 58.0, 28.5 ppm. All spectroscopic data were consistent with those previously reported.^34^

#### Benzyl (*S*)-(1-Oxo-3-phenylpropan-2-yl)carbamate (**1o**)

Prepared following the general procedure using commercially available Cbz-Phe-OH (1.50 g, 5.01 mmol), which was dissolved in CH_2_Cl_2_ (35 mL). The colorless solution was treated with CDI (0.893 g, 5.51 mmol) at 0 °C for 60 min, and subsequently, dropwise with DIBAL-H (10.5 mL, 10.5 mmol). The mixture was stirred for 60 min at −78 °C. Extractive work-up afforded compound **1o** as a white powder (0.825 g, 68% yield). ^**1**^**H NMR** (700 MHz, CDCl_3_) δ 9.66 (s, 1H, CHO), 7.39–7.16 (m, 10H, Ar-H), 5.34 (m, 1H, NH), 5.14 (s, 2H, CH_2_O), 4.56–4.53 (q, *J* = 6.6 Hz, 1H, CHCHO), 3.16 (d, *J* = 6.2 Hz, 2H, CH_2_) ppm. ^**13**^**C**{^1^H} **NMR** (175 MHz, CDCl_3_) δ 198.8, 155.9, 136.1, 135.4, 129.3, 129.0, 128.6, 128.6, 128.3, 128.2, 127.2, 67.2, 61.1, 35.4 ppm. All spectroscopic data were consistent with those previously reported.^12^

#### (*S*)-*N*-(1-Oxo-3-phenylpropan-2-yl)benzamide (**1p**)

Prepared following the general procedure using commercially available Bz-Phe-OH (1.50 g, 5.57 mmol), which was dissolved in CH_2_Cl_2_ (39 mL). The colorless solution was treated with CDI (0.992 g, 6.12 mmol) at 0 °C for 60 min, and subsequently, dropwise with DIBAL-H (11.7 mL, 11.7 mmol). The mixture was stirred for 60 min at −78 °C. Extractive work-up afforded compound **1p** as a white powder (0.600 g, 42% yield). ^**1**^**H NMR** (700 MHz, CDCl_3_) δ 9.76 (s, 1H, CHO), 7.77–7.75 (m, 2H, Ar-H), 7.56–7.43 (m, 3H, Ar-H), 7.32–7.30 (m, 3H, Ar-H), 7.23–7.21 (m, 2H, Ar-H), 6.74 (m, 1H, NH), 4.98–4.94 (m, 1H, CHNH), 3.36–3.31 (m, 2H, CH_2_) ppm. ^**13**^**C**{^1^H} **NMR** (175 MHz, CDCl_3_) δ 199.5, 155.4, 149.5, 135.5, 130.3, 124.7, 124.2, 122.8, 119.0, 115.3, 114.8, 83.8, 80.3, 28.2, 25.1 ppm. All spectroscopic data were consistent with those previously reported.^35^

#### (S)-*N*-(1-Oxo-3-phenylpropan-2-yl)acetamide (**1q**)

Prepared following the general procedure using commercially available *N*-Ac-Phe-OH (1.50 g, 7.24 mmol), which was dissolved in CH_2_Cl_2_ (51 mL). The colorless solution was treated with CDI (1.29 g, 7.96 mmol) at 0 °C for 60 min, and subsequently, dropwise with DIBAL-H (15.2 mL, 15.2 mmol). The mixture was stirred for 60 min at −78 °C. Extractive work-up afforded compound **1q** as a white powder (0.200 g, 15% yield). ^**1**^**H NMR** (700 MHz, CDCl_3_) δ 9.63 (s, 1H, CHO), 7.32–7.30 (m, 2H, Ar-H), 7.27–7.25 (m, 1H, Ar-H), 7.16–7.14 (m, 2H, Ar-H), 5.98 (s, 1H, NH), 4.75–4.72 (m, 1H, CHNH), 3.20–3.15 (m, 1H, CH_2_), 2.01 (s, 3H, CH_3_) ppm. ^**13**^**C**{^1^H} **NMR** (175 MHz, CDCl_3_) δ 198.8, 170.1, 135.5, 129.3, 128.8, 127.2, 59.8, 35.1, 23.1 ppm. All spectroscopic data were consistent with those previously reported.^36^

### Synthesis of Boc-Val-Phe (**1r**)

#### *N*-Boc-Val-Phe-OMe

According to the previously reported procedure,^12^ Boc-Val-OH (0.700 g, 3.22 mmol) was dissolved in *N*,*N*-dimethylformamide (DMF) (20 mL) and Hünig’s base (1.67 mL, 12.9 mmol) was added to the stirred solution. After cooling to 0 °C, HBTU (1.46 g, 3.86 mmol) was added in one portion. After 5 min of activation time, Phe-OMe·HCl (0.764 g, 3.54 mmol) was added, the ice bath was removed, and the reaction mixture was stirred for 50 min. Extractive work-up and purification afforded *N-Boc-Val-Phe-OMe* as a white powder (1.05 g, 86% yield). ^**1**^**H NMR** (700 MHz, CDCl_3_) δ = 7.32–7.28 (m, 2H, Ar–H), 7.26–7.24 (m, 1H, Ar–H), 7.11–7.10 (m, 2H, Ar–H), 6.25 (s, 1H, OCNH), 4.99–4.98 (d, ^3^*J* = 7.1 Hz, 1H, O_2_CNH), 4.89–4.86 (q, ^3^*J* = 6.1 Hz, 1H, BnCH), 3.90–3.87 (m, 1H, C*H*(CH_3_)_2_), 3.71 (s, 3H, CO_2_CH_3_), 3.13–3.11 (m, 2H, PhCH_2_), 2.10–2.07 (m, 1H, CH(CH_3_)_2_), 1.45 (s, 9H, (CH_3_)_3_), 0.93 (d, ^3^*J* = 6.8 Hz, 3H, CHCH_3_CH_3_), 0.87 (d, ^3^*J* = 5.6 Hz, 3H, CHCH_3_CH_3_) ppm. ^**13**^**C**{^1^H} **NMR** (175 MHz, CDCl_3_) δ = 172.0, 171.2, 155.9, 135.6, 129.3, 128.7, 127.2, 80.0, 59.9, 53.1, 52.3, 38.6, 30.9, 28.3, 19.2, 17.6 ppm. All spectroscopic data were consistent with those previously reported.^12^

#### *N*-Boc-Val-Phe-OH

According to the previously reported procedure,^12^ Boc-Val-Phe-OMe (1.05 g, 2.78 mmol) were dissolved in THF (25 mL). A solution of LiOH·H_2_O (0.466 g, 11.1 mmol) in H_2_O (10 mL) was added under vigorous stirring. After full conversion was indicated by TLC, EtOAc (5 mL) was added, and the pH was adjusted to 4 with 25% aqueous citric acid. Extractive work-up and purification via silica gel filtration afforded *N-Boc-Val-Phe-OH* as a white solid (0.800 g, 79% yield). ^**1**^**H NMR** (700 MHz, CDCl_3_) δ = 7.30–7.06 (m, 5H, Ar–H), 5.24 (d, 1H, O_2_CNH), 4.73 (m, 1H, BnCH), 3.96–3.94 (m, 1H, CH(CH_3_)_2_), 3.16 (m, 1H, PhCHH), 2.95 (m, ^3^*J* = 6.5 Hz, 1H, PhCHH), 1.96 (m, 1H, CH(CH_3_)_2_), 1.43 (s, 9H, (CH_3_)_3_), 0.83–0.82 (d, ^3^*J* = 6.8 Hz, 3H, CH(CH_3_)_2_), 0.79–0.78 (d, 3H, CH(CH_3_)_2_) ppm. ^**13**^**C**{^1^H} **NMR** (175 MHz, CDCl_3_) δ = 175.8, 171.7, 156.0, 135.8, 130.3, 128.6, 127.1, 80.3, 60.5, 53.2, 38.7, 32.2, 28.3, 19.1, 17.8 ppm. All spectroscopic data were consistent with those previously reported.^12^

#### *N*-Boc-Val-Phe-H (**1r**)

This compound was synthesized according to previously reported.^12^ Boc-Val-Phe-OH (0.150 g, 0.400 mmol) was dissolved in CH_2_Cl_2_ (8 mL), and the solution was cooled to 0 °C and CDI (0.080 g, 0.480 mmol) was added. After stirring for 60 min, the reaction mixture was cooled to −78 °C for 15 min. Subsequently, DIBAL-H solution (1.24 mL, 1.2 mmol, 1.0 M in toluene) was added dropwise with a syringe through the septum at a rate of 2.0 mL/h. The reaction mixture was stirred at −78 °C until TLC indicated quantitative conversion (150 min). Extractive work-up and column yielded compound **1r** (125 mg, 90% yield) yield as a white powder. ^**1**^**H NMR** (700 MHz, CDCl_3_) δ = 9.62 (s, 1H, CHO), 7.33–7.18 (m, 5H, Ar–H), 6.62 (br s, 1H, HNCO), 5.04–5.03 (m, 1H, HNCO_2_), 4.73–4.72 (m, 1H, BnCH), 3.95–3.93 (m, 1H, *i*-PrCH), 3.17–3.16(m, 2H, PhCH_2_), 2.14–2.12 (m, 1H, (CH_3_)_2_CH), 1.46 (s, 9H, (CH_3_)_3_), 0.95 (d, 3J = 6.8 Hz, 3H, CHCH_3_CH_3_), 0.89 (d, ^3^*J* = 5.6 Hz, 3H, CHCH_3_CH_3_) ppm. ^**13**^**C**{^1^H} **NMR** (175 MHz, CDCl_3_) δ = 198.6, 171.9, 155.8, 135.5, 129.3, 128.8, 127.2, 80.1, 60.0, 52.8, 35.3, 30.3, 28.3, 19.3, 17.7 ppm. All spectroscopic data were consistent with those previously reported.^12^

### Synthesis of 1,3-Diacylpyrroles

#### General Procedure A

In a 10 mL round-bottom flask, 1,3-dicarbonyl (0.300 mmol), ZrOCl_2_·8H_2_O (0.030–0.090 mmol, 10–30 mol %), and H_2_O (0.2 mL) were combined and allowed to stir. α-Aminoaldehyde (0.300 mmol), dissolved in THF (0.4 mL), was added dropwise to the reaction mixture. The mixture was stirred for 6 h at room temperature (RT) or 50 °C (oil bath). The reaction was stopped, and the product was extracted with ethyl acetate and dried over Na_2_SO_4_ and concentrated via rotary evaporation. After work-up, the residue was purified by flash chromatography on silica gel (20% EtOAc/Hexanes).

#### General Procedure B

In a 10 mL round-bottom flask, 1,3-dicarbonyl (0.300 mmol), ZrOCl_2_·8H_2_O (0.030–0.090 mmol, 10–30 mol %), and H_2_O (0.2 mL) were combined and allowed to stir. α-Aminoaldehyde (0.300 mmol), dissolved in THF (0.4 mL), was added dropwise to the reaction mixture followed by the addition of Amberlyst-15 (0.030 mmol, 10 mol %). The mixture was stirred for 6 h at RT or 50 °C (oil bath). The reaction was stopped, and the product was extracted with ethyl acetate, dried over Na_2_SO_4_, and concentrated via rotary evaporation. After work-up, the residue was purified by flash chromatography on silica gel (20% EtOAc/Hexanes).

##### *tert*-Butyl 3-Acetyl-2-methyl-5-phenyl-1*H*-pyrrole-1-carboxylate (**3aa**)

Prepared following general procedure A using Boc-Phe-H **1a** (75 mg, 0.300 mmol) in THF (0.4 mL), acetylacetone **2a**, (0.031 mL, 0.300 mmol), ZrOCl_2_·8H_2_O (9.7 mg, 0.030 mmol), and H_2_O (0.2 mL), and the solution was allowed to stir at room temperature for 6 h. Extractive work-up afforded compound **3aa** as a yellow oil (81 mg, 86% yield) without further purification. ^**1**^**H NMR** (700 MHz, CDCl_3_) δ 7.28 (t, ^3^*J* = 7.6 Hz, 2H, Ar-H), 7.21 (t, 3J = 7.4 Hz, 1H, Ar-H), 7.11–7.10 (m, 2H, Ar-H), 6.13 (s, 1H, Pyrrole-H), 4.13 (s, 2H, CH_2_Ph), 2.69 (s, 3H, CH_3_), 2.36 (s, 3H, CH_3_), 1.41 (s, 9H, (CH_3_)_3_CO) ppm. ^**13**^**C**{^1^H} **NMR** (175 MHz, CDCl_3_) δ 195.9, 149.8, 139.2, 137.7, 132.5, 128.6, 126.5, 122.3, 112.4, 85.2, 34.9, 29.6, 27.7,14.3 ppm. **IR** (film) 3308 (s), 2978 (w), 1745 (s), 1645 (s), 1300 (m), 1280 (m), 1216 (m), 1122 (s) cm^–1^. **HRMS (ESI)** (*m*/*z*): [M + H]^+^ calcd for C_19_H_24_O_3_N, 314.1751; found, 314.1745.

##### *tert*-Butyl 3-Acetyl-2-methyl-5-phenyl-1*H*-pyrrole-1-carboxylate (**3ba**)

Prepared following general procedure A using Boc-Ala-H **1b** (52 mg, 0.300 mmol) in THF (0.4 mL), acetylacetone **2a** (0.031 mL, 0.300 mmol), ZrOCl_2_·8H_2_O (9.7 mg, 0.030 mmol), and H_2_O (0.2 mL), and the solution was allowed to stir at room temperature for 6 h. Extractive work-up afforded compound **3ba** as a white solid (53 mg, 75% yield) without further purification. Mp 50–51 °C. ^**1**^**H NMR** (700 MHz, CDCl_3_) δ 6.17 (s, 1H, Pyrrole-H), 2.69 (s, 3H, CH_3_), 2.37 (s, 3H, CH_3_), 2.34 (s, 3H, CH_3_), 1.60 (s, 9H, (CH_3_)_3_CO) ppm. ^**13**^**C**{^1^H} **NMR** (175 MHz, CDCl_3_) δ 195.8, 150.0, 137.1, 130.0, 122.3, 111.1, 85.0, 29.6, 28.1, 15.8, 14.4 ppm. **IR** (film) 2979 (m), 1748 (s), 1668 (s), 1537 (m), 1260 (s), 1172 (s), 1020 (s) cm^–1^. **HRMS (ESI)** (*m*/*z*): [M + H]^+^ calcd for C_13_H_20_O_3_N, 238.1437; found, 238.1434.

##### 1.50 mmol Scale-up of **3ba**

Prepared following general procedure A using Boc-Ala-H **1b** (259.8 mg, 1.50 mmol) in THF (2.5 mL), acetylacetone **2a** (0.154 mL, 1.50 mmol), ZrOCl_2_·8H_2_O (48.3 mg, 0.15 mmol), and H_2_O (1.25 mL), and the solution was allowed to stir at room temperature for 6 h. Extractive work-up afforded compound **3ba** as a white solid (260 mg, 75% yield) without further purification.

##### *tert*-Butyl 3-Acetyl-5-isopropyl-2-methyl-1*H*-pyrrole-1-carboxylate (**3ca**)

Prepared following general procedure A using Boc-Val-H **1c** (60 mg, 0.300 mmol) in THF (0.4 mL), acetylacetone **2a** (0.031 mL, 0.300 mmol), ZrOCl_2_·8H_2_O (9.7 mg, 0.030 mmol), and H_2_O (0.2 mL), and the solution was allowed to stir at room temperature for 6 h. Extractive work-up afforded compound **3ca** as a yellow oil (66 mg, 83% yield) without further purification. ^**1**^**H NMR** (700 MHz, CDCl_3_) δ 6.21 (s, 1H, Pyrrole-H), 3.32 (m, 1H, C*H*(CH_3_)_2_), 2.65 (s, 3H, CH_3_), 2.39 (s, 3H, CH_3_), 1.61 (s, 9H, (CH_3_)_3_CO), 1.21 (d, ^3^*J* = 6.9 Hz, 6H, CH_3_) ppm. ^**13**^**C**{^1^H} **NMR** (175 MHz, CDCl_3_) δ 195.8, 150.1, 136.8, 133.3, 122.0,107.3, 85.2, 29.5, 28.1, 26.5, 23.0, 14.1 ppm. **IR** (film) 2970 (m), 1748 (s), 1668 (s), 1533 (m), 1248 (s), 1152 (s), 1118 (s) cm^–1^. **HRMS (ESI)** (*m*/*z*): [M + H]^+^ calcd for C_15_H_23_O_3_N, 266.1751; found, 266.1747.

##### *tert*-Butyl 3-Acetyl-5-isobutyl-2-methyl-1*H*-pyrrole-1-carboxylate (**3da**)

Prepared following general procedure A using Boc-Leu-H **1d** (65 mg, 0.300 mmol) in THF (0.4 mL), acetylacetone **2a** (0.031 mL, 0.300 mmol), ZrOCl_2_·8H_2_O (9.7 mg, 0.030 mmol), and H_2_O (0.2 mL), and the solution was allowed to stir at room temperature for 6 h. Extractive work-up afforded compound **3da** as a yellow oil (55 mg, 64% yield) without further purification. ^**1**^**H NMR** (700 MHz, CDCl_3_) δ 6.19 (s, 1H, Pyrrole-H), 2.69 (s, 3H, CH_3_), 2.63 (d, ^3^*J* = 7.1Hz, 2H, CH_2_), 2.41 (s, 3H, CH_3_), 1.85–1.77 (m, 1H, C*H*(CH_3_)_2_), 1.63 (s, 9H, (CH_3_)_3_CO), 0.93 (d, ^3^*J* = 6.6 Hz, 6H, CH_3_) ppm. ^**13**^**C**{^1^H} **NMR** (175 MHz, CDCl_3_) δ 195.8, 150.1, 137.1, 141.1, 136.6, 122.0, 111.2, 85.2, 37.6, 2 9.4, 28.1, 27.9, 22.4 14.1 ppm. **IR** (film) 2957 (m), 1748 (s), 1669 (s), 1533 (m), 1276 (s), 1153 (s), 1120 (s) cm^–1^. **HRMS (ESI)** (*m*/*z*): [M + H]^+^ calcd for C_16_H_26_O_3_N, 280.1907; found, 280.1906.

##### *tert*-Butyl (*S*)-3-Acetyl-5-(*sec*-butyl)-2-methyl-1*H*-pyrrole-1-carboxylate ((*S*)-**3ea**)

Prepared following general procedure A using (2*S*,3*S*)-Boc-Ile-H **1e** (65 mg, 0.300 mmol) in THF (0.4 mL), acetylacetone **2a** (0.031 mL, 0.300 mmol), ZrOCl_2_·8H_2_O (9.7 mg, 0.030 mmol), and H_2_O (0.2 mL), and the solution was allowed to stir at room temperature for 6 h. Extractive work-up afforded compound (*S*)-**3ea** as a yellow oil (53 mg, 63% yield) without further purification. ^**1**^**H NMR** (700 MHz, CDCl_3_) δ 6.19 (s, 1H, Pyrrole-H), 3.12 (q, ^3^*J* = 6.7 Hz 1H, CHCH_3_), 2.64 (s, 3H, CH_3_), 2.39 (s, 3H, CH_3_), 1.68–1.64 (m, 2H, CH_2_CH_3_), 1.61 (s, 9H, (CH_3_)_3_CO), 1.19 (d, 3J = 6.8 Hz, 3H, CH_3_), 0.90 (t, ^3^*J* = 7.4 Hz, 3H, CH_3_) ppm. ^**13**^**C**{^1^H} **NMR** (175 MHz, CDCl_3_) δ 195.7, 150.1, 139.9, 136.2, 122.0, 107.7, 85.0, 32.8, 29.8, 29.4, 27.9, 20.0, 14.1, 11.6 ppm. **IR** (film) 2968 (m), 1749 (s), 1668 (s), 1531 (m), 1244 (s), 1151 (s), 1120 (s) cm^–1^. **HRMS (ESI)** (*m*/*z*): [M + H]^+^ calcd for C_16_H_26_O_3_N, 280.1907; found, 280.1903.

##### *tert*-Butyl 3-Acetyl-5-(4-(benzyloxy)benzyl)-2-methyl-1*H*-pyrrole-1-carboxylate (**3fa**)

Prepared following general procedure A using Boc-Tyr(OBzl)-H **1f** (107 mg, 0.300 mmol) in THF (0.4 mL), acetylacetone **2a** (0.031 mL, 0.300 mmol), ZrOCl_2_·8H_2_O (14.5 mg, 0.045 mmol), and H_2_O (0.2 mL), and the solution was allowed to stir at room temperature for 6 h. Extractive work-up afforded compound **3fa** as a yellow oil (91 mg, 72% yield) without further purification. ^**1**^**H NMR** (700 MHz, CDCl_3_) δ 7.42 (d, ^3^*J* = 8.3, 7.0 Hz, 2H, Ar-H),7.37 (t, ^3^*J* = 7.5 Hz, 2H, Ar-H), 7.31 (t, ^3^*J* = 7.1 Hz, 1H, Ar-H), 7.03 (d, ^3^*J* = 8.3 Hz, 2H, Ar-H), 6.90 (d, ^3^*J* = 8.6 Hz, 2H, Ar-H), 6.09 (s, 1H, Pyrrole-H), 5.05 (s, 2H, CH_2_), 4.06 (s, 2H, CH_2_), 2.69 (s, 3H, CH_3_), 2.35 (s, 3H, CH_3_), 1.44 (s, 9H, (CH_3_)_3_CO) ppm. ^**13**^**C**{^1^H} **NMR** (175 MHz, CDCl_3_) δ 195.7, 157.3, 149.7, 137.5, 137.1, 133.0, 131.4, 129.5, 128.6, 127.9, 127.4, 122.1, 114.9, 112.0, 85.1, 34.0, 29.5, 27.7, 14.1 ppm. **IR** (film) 2978 (m), 1748 (s), 1668 (s), 1509 (m), 1216 (s), 1121 (s) cm^–1^. **HRMS (ESI)** (*m*/*z*): [M + H]^+^ calcd for C_26_H_30_O_4_N, 420.2169; found, 420.2162.

##### *tert*-Butyl 3-((4-Acetyl-1-(*tert*-butoxycarbonyl)-5-methyl-1*H*-pyrrol-2-yl)methyl)-1*H*-indole-1-carboxylate (**3ga**)

Prepared following general procedure B using Boc-Trp(Boc)-H **1g** (117 mg, 0.300 mmol) in 1,4-dioxane (0.4 mL), acetylacetone **2a** (0.031 mL, 0.300 mmol), ZrOCl_2_·8H_2_O (9.7 mg, 0.030 mmol), Amberlyst-15 (9.4 mg, 0.030 mmol), and H_2_O (0.2 mL), and the solution was allowed to stir at 60 °C (oil bath) for 6 h. Extractive work-up afforded compound **3ga** as a red oil (88 mg, 65% yield) without further purification. ^**1**^**H NMR** (700 MHz, CDCl_3_) δ 8.13 (s, 1H, Ar-H), 7.46 (d, ^3^*J* = 7.8 Hz, 1H, Ar-H), 7.33–7.30 (m, 3H, Ar-H), 7.23 (t, ^3^*J* = 7.3 Hz, 2H, CH_2_), 6.17 (s, 1H, Pyrrole-H), 4.17 (s, 2H, CH_2_), 2.70 (s, 3H, CH_3_), 2.31 (s, 3H, CH_3_), 1.66 (s, 9H, (CH_3_)_3_CO), 1.49 (s, 9H, (CH_3_)_3_CO) ppm. ^**13**^**C**{^1^H} **NMR** (175 MHz, CDCl_3_) δ 195.7, 149.8, 138.8, 137.3, 131.5, 130.2, 124.5, 123.8, 122.5, 122.2, 119.2, 118.1, 115.3, 111.7, 85.7, 83.6, 29.5, 28.2, 27.8, 24.9, 14.2, 14.2 ppm. **IR** (film) 2979 (m), 1732 (s), 1668 (s), 1533 (m), 1452 (m), 1370 (s), 1256 (m), 1160 (s), 1122 (m) cm^–1^. **HRMS (ESI)** (*m*/*z*): [M + H]^+^ calcd for C_26_H_33_O_5_N_2_, 453.2383; found, 453.2375.

##### *tert*-Butyl 3-Acetyl-5-((benzyloxy)methyl)-2-methyl-1*H*-pyrrole-1-carboxylate (**3ha**)

Prepared following general procedure A using Boc-Ser(OBzl)-H **1h** (84 mg, 0.300 mmol) in THF (0.4 mL), acetylacetone **2a** (0.031 mL, 0.300 mmol), ZrOCl_2_·8H_2_O (9.7 mg, 0.030 mmol), and H_2_O (0.2 mL), and the solution was allowed to stir at room temperature for 6 h. Extractive work-up afforded compound **3ha** as a yellow oil (58 mg, 56% yield) without further purification. ^**1**^**H NMR** (700 MHz, CDCl_3_) 7.35–7.29 (m, 5H, Ar-H), 6.48 (s, 1H, Pyrrole-H), 4.63 (s, 3H, CH_3_), 4.52 (s, 3H, CH_3_), 2.71 (s, 3H, CH_3_), 2.40 (s, 3H, CH_3_), 1.58 (s, 9H, (CH_3_)_3_CO) ppm. ^**13**^**C**{^1^H} **NMR** (175 MHz, CDCl_3_) δ 195.6, 149.5, 138.6, 138.1, 130.2, 128.4, 127.8,127.7, 122.0, 113.0, 85.2, 72.0, 65.4, 29.4, 27.8, 13.4 ppm. **IR** (film) 2979 (w), 2931 (w), 1751 (s), 1669 (m), 1533 (m), 1280 (m), 1216 (m), 1128 (m) cm^–1^. **HRMS (ESI)** (*m*/*z*): [M + H]^+^ calcd for C_20_H_26_O_4_N, 344.1856; found, 344.1851.

##### *tert*-Butyl 3-Acetyl-5-((benzylthio)methyl)-2-methyl-1*H*-pyrrole-1-carboxylate (**3ia**)

Prepared following general procedure B using Boc-Cys(Bzl)-H **1i** (89 mg, 0.300 mmol) in 1,4-dioxane (0.4 mL), acetylacetone **2a** (0.031 mL, 0.300 mmol), ZrOCl_2_·8H_2_O (9.7 mg, 0.030 mmol), Amberlyst-15 (9.4 mg, 0.030 mmol), and H_2_O (0.2 mL), and the solution was allowed to stir at 50 °C (oil bath) for 6 h. Extractive work-up afforded compound **3ia** as a yellow oil (90 mg, 83% yield) without further purification. ^**1**^**H NMR** (700 MHz, CDCl_3_) δ 7.30 (t, ^3^*J* =7.5 Hz, 2H, Ar-H), 7.26 (d, ^3^*J* = 7.5 Hz, 2H, Ar-H), 7.23 (t, ^3^*J* = 7.5 Hz, 1H, Ar-H), 6.23 (s, 1H, Pyrrole-H), 3.74 (s, 2H, CH_2_), 3.59 (s, 2H, CH_2_), 2.70 (s, 3H, CH_3_), 2.38 (s, 3H, CH_3_), 1.61 (s, 9H, (CH_3_)_3_CO), 1.49 (s, 9H, (CH_3_)_3_CO) ppm. ^**13**^**C**{^1^H} **NMR** (700 MHz, CDCl_3_) δ 195.4, 149.5, 138.8, 138.2, 129.5, 129.1,128.6, 127.1, 121.8, 112.9, 85.7, 35.5, 29.6, 29.1, 28.0, 14.2 ppm. **IR** (film) 2977 (m), 2928 (m), 1747 (s), 1667 (s), 1529 (m), 1281 (s), 1154 (s), 1117 (s) cm^–1^. **HRMS (ESI)** (*m*/*z*): [M + H]^+^ calcd for C_20_H_26_O_3_NS, 360.1628; found, 360.1621.

##### *tert*-Butyl 3-Acetyl-2-methyl-5-(2-(methylthio)ethyl)-1*H*-pyrrole-1-carboxylate (**3ja**)

Prepared following general procedure A using Boc-Met-H **1j** (70 mg, 0.300 mmol) in THF (0.4 mL), acetylacetone **2a** (0.031 mL, 0.300 mmol), ZrOCl_2_·8H_2_O (14.5 mg, 0.045 mmol), and H_2_O (0.2 mL), and the solution was allowed to stir at 50 °C (oil bath) for 6 h. Extractive work-up afforded compound **3ja** as a yellow oil (56 mg, 63% yield) without further purification. ^**1**^**H NMR** (700 MHz, CDCl_3_) δ 6.27 (s, 1H, Pyrrole-H), 3.04 (t, ^3^*J* = 7.7 Hz, 2H, CH_2_), 2.72 (m, 2H, CH_2_), 2.68 (s, 3H, CH_3_), 2.39 (s, 3H, CH_3_), 2.13 (s, 3H, CH_3_), 1.62 (s, 9H, (CH_3_)_3_CO) ppm. ^**13**^**C**{^1^H} **NMR** (175 MHz, CDCl_3_) δ 195.8, 149.9, 137.3, 132.7, 122.4, 111.2, 85.4, 33.9, 29.6, 29.2, 28.1, 15.8, 14.4 ppm. **IR** (film) 2979 (m), 2919 (m), 1747 (s), 1668 (s), 1533 (m), 1212 (s), 1156 (s), 1120 (s) cm^–1^. **HRMS (ESI)** (*m*/*z*): [M + H]^+^ calcd for C_15_H_24_O_3_NS, 298.1471; found, 298.1466.

##### *tert*-Butyl 3-Acetyl-2-methyl-5-phenyl-1*H*-pyrrole-1-carboxylate (**3la**)

Prepared following general procedure A using Boc-Phg-H **1l** (71 mg, 0.300 mmol) in THF (0.4 mL), acetylacetone **2a** (0.031 mL, 0.300 mmol), ZrOCl_2_·8H_2_O (9.7 mg, 0.030 mmol), and H_2_O (0.2 mL), and the solution was allowed to stir at room temperature for 6 h. Extractive work-up afforded compound **3la** as a yellow oil (81 mg, 90% yield) without further purification. ^**1**^**H NMR** (700 MHz, CDCl_3_) δ 7.37 (m, 2H, Ar-H), 7.33 (m, 1H, Ar-H), 7.30 (m, 2H, Ar-H), 6.47 (s, 1H, Pyrrole-H), 2.77 (s, 3H, CH_3_), 2.44 (s, 3H, CH_3_), 1.27 (s, 9H, (CH_3_)_3_CO) ppm. ^**13**^**C**{^1^H} **NMR** (175 MHz, CDCl_3_) δ 195.6, 149.6, 138.3, 134.0, 133.8, 128.3, 127.6, 122.6, 112.4, 85.2, 29.5, 27.4, 13.4 ppm. **IR** (film) 3057 (w), 2980 (s), 2931 (m), 1736 (s), 1667 (s), 1530 (m), 1230 (m), 1144 (s) cm^–1^. **HRMS (ESI)** (*m*/*z*): [M + H]^+^ calcd for C_18_H_22_O_3_N, 300.1741; found, 300.1745.

##### *tert*-Butyl 3-Acetyl-5-allyl-2-methyl-1*H*-pyrrole-1-carboxylate (**3ma**)

Prepared following general procedure A using Boc-allylGly-H **1m** (60 mg, 0.300 mmol) in THF (0.4 mL), acetylacetone **2a** (0.031 mL, 0.300 mmol), ZrOCl_2_·8H_2_O (9.7 mg, 0.030 mmol), and H_2_O (0.2 mL), and the solution was allowed to stir at 50 °C (oil bath) for 6 h. Extractive work-up afforded compound **3ma** as a yellow oil (44 mg, 56% yield) without further purification. ^**1**^**H NMR** (700 MHz, CDCl_3_) δ 6.22 (s, 1H, Pyrrole-H), 5.96–5.91 (m, 1H, =CH), 5.10 (dd, ^3^*J* = 10.1, ^*2*^*J* = 1.6 Hz, 1H, = CH_2_), 5.04 (dd, ^3^*J* = 17.1, ^*2*^*J* = 1.6 Hz, 1H, =CH_2_), 3.51 (d, ^3^*J* = 6.0, 2H, CH_2_), 2.69 (s, 3H, CH_3_), 2.38 (s, 3H, CH_3_), 1.60 (s, 9H, (CH_3_)_3_CO) ppm. ^**13**^**C**{^1^H} **NMR** (700 MHz, CDCl_3_) δ 195.8, 149.9, 137.4, 135.4, 132.2, 122.3, 116.7, 111.1, 85.2, 33.2, 29.6, 28.0, 14.1 ppm. **IR** (film) 2979 (m), 2931 (w), 1748 (s), 1668 (s), 1533 (m), 1121 (s) cm^–1^. **HRMS (ESI)** (*m*/*z*): [M + H]^+^ calcd for C_15_H_22_O_3_N, 264.1594; found, 264.1590.

##### *tert*-Butyl 3-Acetyl-2-methyl-5-(prop-2-*yn*-1-yl)-1*H*-pyrrole-1-carboxylate (**3na**)

Prepared following general procedure A using Boc-propargyl-Gly-H **1n** (60 mg, 0.300 mmol) in THF (0.4 mL), acetylacetone **2a** (0.031 mL, 0.300 mmol), ZrOCl_2_·8H_2_O (9.7 mg, 0.030 mmol), and H_2_O (0.2 mL), and the solution was allowed to stir at 50 °C (oil bath) for 6 h. Extractive work-up afforded compound **3na** as a yellow oil (54 mg, 68% yield) without further purification. ^**1**^**H NMR** (700 MHz, CDCl_3_) δ 6.49 (s, 1H, Pyrrole-H), 3.72 (m, 2H, CH_2_), 2.72 (s, 3H, CH_3_), 2.40 (s, 3H, CH_3_), 2.15 (t, ^3^*J* = 2.6 Hz, 1H, ≡CH), 1.62 (s, 9H, (CH_3_)_3_CO) ppm. ^**13**^**C**{^1^H} **NMR** (175 MHz, CDCl_3_) δ 195.6, 149.5, 138.2, 128.3, 122.2, 111.8, 85.6, 80.6, 70.1, 29.5, 27.9, 19.9, 14.1 ppm. **IR** (film) 3287 (s), 2980 (m), 2931 (w), 1750 (s), 1669 (s), 1534 (m), 1122 (m) cm^–1^. **HRMS (ESI)** (*m*/*z*): [M + H]^+^ calcd for C_15_H_20_O_3_N, 262.1437; found, 262.1434.

##### Benzyl 3-Acetyl-5-benzyl-2-methyl-1*H*-pyrrole-1-carboxylate (**3oa**)

Prepared following general procedure A using Cbz-Phe-H **1o** (85 mg, 0.300 mmol) in 1,4-dioxane (0.4 mL), acetylacetone 2a (0.031 mL, 0.300 mmol), ZrOCl_2_·8H_2_O (29 mg, 0.090 mmol), and H_2_O (0.2 mL), and the solution was allowed to stir at room temperature for 6 h. Extractive work-up afforded compound **3oa** as a yellow oil (78 mg, 75% yield) without further purification. ^**1**^**H NMR** (700 MHz, CDCl_3_) δ 7.38–7.35 (m, 3H, Ar-H), 7.31–7.25 (m, 4H, Ar-H), 7.23–7.20 (m, 1H, Ar-H), 7.07–7.03 (m, 2H, Ar-H), 6.14 (s, 1H, Pyrrole-H), 5.20 (s, 2H, CH_2_), 4.10 (s, 2H, CH_2_), 2.71 (s, 3H, CH_3_), 2.36 (s, 3H, CH_3_) ppm. ^**13**^**C**{^1^H} **NMR** (175 MHz, CDCl_3_) δ 195.6, 151.3, 138.9, 138.2, 134.1, 133.1, 128.9, 128.8, 128.8, 128.5, 128.4, 126.4, 122.7, 113.0, 69.5, 35.2, 29.6,14.2 ppm. **IR** (film) 3029 (m), 2924 (w), 1753 (s), 1667 (s), 1530 (m), 1318 (m), 1266 (s), 1213 (s), 1116 (m) cm^–1^. **HRMS (ESI)** (*m*/*z*): [M + H]^+^ calcd for C_22_H_22_O_3_N, 348.1594; found, 348.1588.

##### 1-(1-Benzoyl-5-benzyl-2-methyl-1*H*-pyrrol-3-yl)ethan-1-one (**3pa**)

Prepared following general procedure B using Bz-Phe-H **1p** (76 mg, 0.300 mmol) in THF (0.4 mL), acetylacetone **2a** (0.031 mL, 0.300 mmol), ZrOCl_2_·8H_2_O (9.7 mg, 0.030 mmol), Amberlyst-15 (9.4 mg, 0.030 mmol), and H_2_O (0.2 mL), and the solution was allowed to stir at 60 °C (oil bath) for 6 h. After work-up, the residue was purified by column chromatography on silica gel (20% EtOAc/Hexanes) to afford compound **3pa** as a yellow solid (42 mg, 44% yield). **Mp** 195–196 °C. ^**1**^**H NMR** (700 MHz, CDCl_3_) δ 7.60 (t, ^3^*J* = 6.9 Hz, 1H, Ar-H), 7.51 (d, ^3^*J* = 7.9 Hz, 2H, Ar-H), 7.41 (t, ^3^*J* = 7.7 Hz, 2H, Ar-H), 7.16 (t, ^3^*J* = 7.5 Hz, 2H, Ar-H), 7.10 (t, ^3^*J* = 7.3 Hz, 1H, Ar-H), 7.01 (d, ^3^*J* = 7.5 Hz, 2H, Ar-H), 6.32 (s, 1H, Pyrrole-H), 3.87 (s, 2H, CH_2_), 2.42 (s, 3H, CH_3_), 2.28 (s, 3H, CH_3_) ppm. ^**13**^**C**{^1^H} **NMR** (175 MHz, CDCl_3_) δ 195.7, 171.1, 138.2, 136.4, 134.6, 133.9, 132.9, 130.5, 129.0, 128.5, 128.5, 126.7, 122.0, 111.3, 33.8, 29.3, 14.1 ppm. **IR** (film) 3063 (w), 2925 (m), 1708 (s), 1664 (s), 1522 (m), 1451 (m), 1403 (m), 1263 (s), 1172 (s), 1215 (s) cm^–1^. **HRMS (ESI)** (*m*/*z*): [M + H]^+^ calcd for C_21_H_20_O_2_N, 318.1488; found, 318.1484.

##### *tert*-Butyl (*S*)-(1-(3-Acetyl-5-benzyl-2-methyl-1*H*-pyrrol-1-yl)-3-methyl-1oxobutan2yl)carbamate ((*S*)-**3ra**)

Prepared following general procedure B using Boc-l-Val-l-Phe-H **1r** (50 mg, 0.143 mmol) in THF (0.4 mL), acetylacetone **2a** (0.015 mL, 0.143 mmol), ZrOCl_2_·8H_2_O (7.1 mg, 0.022 mmol), and H_2_O (0.2 mL), and the solution was allowed to stir at 50 °C (oil bath) for 6 h. After work-up, the residue was purified by column chromatography on basic alumina (40% EtOAc/Hexanes) to afford compound (*S*)-**3ra** as a yellow oil (17 mg, 28% yield). *Enantiomeric Ratio: 99.3:0.7*. ^**1**^**H NMR** (700 MHz, CDCl_3_) 7.29 (t, ^3^*J* = 7.6 Hz, 2H, Ar-H), 7.22 (t, ^3^*J* = 7.4 Hz, 1H, Ar-H), 7.17 (d, ^3^*J* = 7.5 Hz, 2H, Ar-H), 6.20 (s, 1H, Pyrrole-H), 5.05 (s, 2H, CH_2_), 4.63 (s, 3H, CH_3_), 2.73 (s, 3H, CH_3_), 2.38 (s, 3H, CH_3_), 2.10–1.94 (m, 1H, CH), 1.86–1.77 (m, 1H, CH), 1.45 (s, 9H, (CH_3_)_3_CO), 0.83 (d, ^3^*J* = 7.0 Hz, 3H, (CH_3_)_2_CH), 0.61 (d, ^3^*J* = 6.9 Hz, 3H, (CH_3_)_2_CH) ppm. ^**13**^**C**{^1^H} **NMR** (175 MHz, CDCl_3_) δ 207.1, 195.7,155.9, 138.3, 136.0, 132.7, 128.9, 128.7, 126.8, 123.3, 113.7, 80.3, 61.3, 34.6, 31.4, 31.1, 29.6, 28.4, 16.3, 14.4 ppm. **IR** (film) 3359 (m), 2925 (s), 2854 (m), 1716 (s), 1670 (m), 1522 (m), 1457(m), 1203 (m), 1165 (m) cm^–1^. **HRMS** (ESI) (*m*/*z*): [M + H]^+^ calcd for C_24_H_33_O_4_N_2_, 413.2434; found, 413.2429. **HPLC** (RegisReflect C-Amylose A, isopropanol/hexanes = 2/98, flow rate = 1.5 mL/min, *l* = 254 nm) *t*_R_ = 7.956 min (major), *t*_2_ = 9.160 min (minor). *Note*: Boc-d-Val-d-Phe-H gave the (*R*)-enantiomer with a similar yield and a 98.7:1.3 enantiomeric ratio.

##### 1-(*tert*-Butyl) 3-Ethyl 5-benzyl-2-methyl-1*H*-pyrrole-1,3-dicarboxylate (**3ab**)

Prepared following general procedure B using Boc-Phe-H **1a** (75 mg, 0.300 mmol) in THF (0.4 mL), ethyl acetoacetate **2b** (0.038 mL, 0.300 mmol), ZrOCl_2_·8H_2_O (14.5 mg, 0.045 mmol), and Amberlyst-15 (9.4 mg, 0.030 mmol) in THF (0.2 mL), and the solution was allowed to stir at 50 °C (oil bath) for 6 h. Extractive work-up afforded compound **3ab** as a yellow oil (60 mg, 58% yield) without further purification. ^**1**^**H NMR** (700 MHz, CDCl_3_) δ 7.32 (t, ^3^*J* = 7.0 Hz, 2H, Ar-H), 7.24 (d, ^3^*J* = 7.4 Hz, 1H, Ar-H), 7.15 (d, ^3^*J* = 7.4 Hz, 2H, Ar-H), 6.25 (s, 1H, Pyrrole-H), 4.29 (q, ^3^*J* = 7.1 Hz, 2H, CH_2_), 4.17 (s, 2H, CH_2_), 2.74 (s, 3H, CH_3_), 1.46 (s, 9H, (CH_3_)_3_CO), 1.36 (t, ^3^*J* = 7.1 Hz, 3H, CH_3_) ppm. ^**13**^**C**{^1^H} **NMR** (175 MHz, CDCl_3_) δ 165.2, 149.8, 139.4, 138.7, 132.5, 128.6, 128.5, 126.3, 114.4, 112.4, 85.0, 59.9, 35.0, 27.8, 14.6, 14.0 ppm. **IR** (film) 2980 (m), 1750 (s), 1707 (s), 1546 (m), 1215 (s), 1163 (m), 1064 (s) cm^–1^. **HRMS (ESI)***m*/*z*: [M + H]^+^ calcd for C_20_H_26_O_4_N, 344.1856; found, 344.1854.

##### *tert*-Butyl 5-Benzyl-2-ethyl-3-propionyl-1*H*-pyrrole-1-carboxylate (**3ac**)

Prepared following general procedure B using Boc-Phe-H **1a** (75 mg, 0.300 mmol) in THF (0.4 mL), 3,5-heptadione **2c** (35.5 mg, 0.300 mmol), ZrOCl_2_·8H_2_O (14.5 mg, 0.045 mmol), and Amberlyst-15 (9.4 mg, 0.030 mmol) in THF (0.2 mL), and the solution was allowed to stir at 50 °C (oil bath) for 6 h. Extractive work-up afforded compound **3ac** as a dark yellow oil (60 mg, 59% yield) without further purification. ^**1**^**H NMR** (700 MHz, CDCl_3_) δ 7.29 (t, ^3^*J* = 7.7 Hz, 2H, Ar-H), 7.21 (t, ^3^*J* = 7.4 Hz, 1H, Ar-H), 7.11 (d, ^3^*J* = 6.3 Hz, 2H, Ar-H), 6.12 (s, 1H, Pyrrole-H), 4.12 (s, 2H, CH_2_), 3.19 (q, ^3^*J* = 7.3 Hz, 2H, CH_2_), 2.70 (q, ^3^*J* = 7.3 Hz, 2H, CH_2_), 1.42 (s, 9H, (CH_3_)_3_CO), 1.20 (t, ^3^*J* = 7.3 Hz, 3H, CH_3_), 1.12 (t, ^3^*J* = 7.3 Hz, 3H, CH_3_) ppm. ^**13**^**C**{^1^H} **NMR** (175 MHz, CDCl_3_) δ 198.2, 149.8,143.5, 139.3, 132.4, 128.7, 128.6, 126.5, 120.9, 111.7, 85.2, 35.0, 34.4, 27.7, 20.5, 14.4, 8.3 ppm. **IR** (film) 2976 (m), 1749 (s), 1670 (s), 1525 (m), 1315 (m), 1292 (m), 1144 (s) cm^–1^. **HRMS (ESI)***m*/*z*: [M + H]^+^ calcd for C_21_H_28_O_3_N, 342.2063; found, 342.2060.

##### *tert*-Butyl 2-Benzyl-4-oxo-4,5,6,7-tetrahydro-1*H*-indole-1-carboxylate (**3ad**)

Prepared following general procedure B using Boc-Phe-H **1a** (75 mg, 0.300 mmol) in THF (0.4 mL), cyclohexa-1,3-dione **2d** (33.7 mg, 0.300 mmol), ZrOCl_2_·8H_2_O (14.5 mg, 0.045 mmol), and Amberlyst-15 (9.4 mg, 0.030 mmol) in THF (0.2 mL), and the solution was allowed to stir at 70 °C (oil bath) for 6 h. After work-up, the residue was purified by column chromatography on silica gel (20% EtOAc/Hexanes) to afford compound **3ad** as a yellow oil (16 mg, 16% yield). ^**1**^**H NMR** (700 MHz, CDCl_3_) δ 7.33 (t, ^3^*J* = 7.5 Hz, 2H, Ar-H), 7.17 (d, ^3^*J* = 7.3 Hz, 1H, Ar-H), 7.12 (d, ^3^*J* = 7.3 Hz, 2H, Ar-H), 6.16 (s, 1H, Pyrrole-H), 4.15 (s, 2H, CH_2_), 3.06 (t, ^3^*J* = 6.2 Hz, 2H, CH_2_), 2.46 (t, ^3^*J* = 6.6 Hz, 2H, CH_2_), 2.13 (p, ^3^*J* = 6.3 Hz, 2H, CH_2_), 1.48 (s, 9H, (CH_3_)_3_CO) ppm. ^**13**^**C**{^1^H} **NMR** (175 MHz, CDCl_3_) δ 195.1, 146.2,139.0, 135.5, 129.1, 128.6, 127.6, 126.5, 122.5, 108.7, 85.4, 35.5, 28.1, 27.9, 25.5, 23.8 ppm. **IR** (film) 2975 (m), 2928 (w), 1746 (s), 1710 (m), 1671 (m), 1258 (m), 1165 (s), 1134 (s) cm^–1^. **HRMS (ESI)***m*/*z*: [M + H]^+^ calcd for C_20_H_24_O_3_N, 326.1751; found, 326.1749.

##### *tert*-Butyl 5-Benzyl-2-methyl-3-(phenylcarbamoyl)-1*H*-pyrrole-1-carboxylate (**3ae**)

Prepared following general procedure A using Boc-Phe-H **1a** (75 mg, 0.300 mmol) in THF (0.4 mL), 3-oxo-*N*-phenylbutanamide **2e** (0.53 mg, 0.300 mmol), ZrOCl_2_·8H_2_O (14.5 mg, 0.045 mmol), and THF (0.2 mL), and the solution was allowed to stir at room temperature for 6 h. After work-up, the residue was purified by column chromatography on silica gel (20% EtOAc/Hexanes) to afford compound **3ae** as a yellow solid (55 mg, 47% yield). **Mp** 123–124 °C. ^**1**^**H NMR** (700 MHz, CDCl_3_) δ 7.54 (d, ^3^*J* = 7.7 Hz, 2H, Ar-H), 7.34–7.30 (m, 5H, Ar-H, NH), 7.22 (t, ^3^*J* = 7.3 Hz 1H, Ar-H), 7.14 (d, ^3^*J* = 7.2 Hz, 2H, Ar-H), 7.10–7.08 (m, 1H, Ar-H), 5.96 (s, 1H, Pyrrole-H), 4.16 (s, 2H, CH_2_Ph), 2.72 (s, 3H, CH_3_), 1.56 (s, 3H, CH_3_), 1.45 (s, 9H, (CH_3_)_3_CO) ppm. ^**13**^**C**{^1^H} **NMR** (175 MHz, CDCl_3_) δ 163.4, 149.8, 139.0, 138.1, 136.7, 133.4,129.0, 128.7, 128.5, 126.4, 124.0, 120.0, 117.3, 109.2, 85.0, 35.1, 27.7, 14.3, 13.9 ppm. **IR** (film) 3063 (w), 2977 (w), 1749 (s), 1669 (s), 1533 (m), 1280 (m), 1216 (m), 1122 (s) cm^–1^. **HRMS (ESI)***m*/*z*: [M]^+^ calcd for C_24_H_26_O_3_N_2_, 390.1751; found, 390.1745.

##### Reaction of Boc-Glucosamine (**1t**) and Acetylacetone (**2a**)

A solution of Boc-glucosamine **1t** (105 mg, 0.300 mmol) in H_2_O (0.6 mL) was added to a solution of acetylacetone **2a** (0.031 mL, 0.300 mmol), ZrCl_4_ (10.5 mg, 0.045 mmol), and Amberlyst-15 (9.4 mg, 0.030 mmol) in H_2_O (0.3 mL), and the solution was allowed to stir at 80 °C for 6 h. After work-up, a 1.56:1 mixture of **3ta**:**4ta** was obtained as a yellow solid (65 mg, 67% yield). ^**1**^**H NMR** (700 MHz, D_2_O) δ 6.80 (s, 1H), 6.68 (s, 1H), 5.53 (s, 1H), 5.32 (s, 1H), 5.10 (m, 1H), 4.99 (m, 1H), 4.51 (s, 1H), 4.44–4.40 (m, 1H), 4.38 (dd, *J* = 10.3, 3.9 Hz, 1H), 4.02–3.96 (m, 3H), 3.87 (m, 3H), 3.78 (m, 1H), 3.69 (d, *J* = 9.9 Hz, 1H), 3.55 (s, 1H), 3.43–3.37 (m, 1H), 2.60 (s, 3H), 2.56 (s, 3H) 1.56 (s, 9H) ppm. ^**13**^**C**{^1^H} **NMR** (101 MHz, D_2_O) δ 208.6, 199.6, 138.9, 127.5, 120.1, 110.0, 91.3,75.9, 75.6, 75.2, 73.9, 72.5, 71.5, 71.0, 70.8, 69.7, 60.7, 60.6, 30.4, 29.6, 27.6, 13.2 ppm. **HRMS (ESI)***m*/*z*: [M + H]^+^ calcd for C_16_H_24_NO_6_, 326.1598; found, 326.1593.

### Synthesis of Pyrrolizidin-3-one (**6**)

#### 5-Oxopyrrolidine-2-carbaldehyde (**1u**)

Following the procedure outlined in previous literature,^10^ a solution of 5-oxopyrrolidine-2-carboxylic acid (1.18 g, 8.95 mmol) in a mixture of anhydrous CH_2_Cl_2_ (9.38 mL) and anhydrous DMF (5 mL) was cooled to 0 °C. 4-Dimethylaminopyridine (DMAP) (110 mg. 0.9 mmol), ethanethiol (880 mg, 10.7 mmol), and dicyclohexylcarbodiimide (DCC, 2.20 g, 10.7 mmol) were added sequentially. The reaction mixture was allowed to warm to room temperature and stirred for 16 h. After work-up, the residue was purified by column chromatography on silica gel (90% EtOAc/Hexanes) to afford ethyl 5-oxopyrrolidine-2-carbothioate **7** as a yellow oil (347 mg, 22% yield). ^**1**^**H NMR** (500 MHz, CDCl_3_) δ 6.44 (bs, 1H, NH), 4.30–4.27 (m, 1H, CHNH), 2.94–2.89 (m, 2H, CH_2_), 2.46 (m, 2H, CH_2_), 2.32 (m, 1H, CH_2_), 2.19 (m, 1H, CH_2_), 1.28–1.26 (m, 3H, CH_3_). ^**13**^**C**{^1^H} **NMR** (125 MHz, CDCl_3_) δ 201.2,178.7, 62.6, 28.9, 26.1, 23.4, 14.6 ppm. All spectroscopic data were consistent with those previously reported.^37^ A solution of resulting ethyl 5-oxopyrrolidine-2-carbothioate (347 mg, 2.00 mmol) in MeCN (5 mL) was cooled to 0 °C. Boc_2_O (524 mg, 2.40 mmol) and DMAP (25 mg, 0.200 mmol) were sequentially added, and the solution was allowed to stir at room temperature for 1 h. After work-up, the residue was purified by column chromatography on silica gel (20% EtOAc/Hexanes) to afford *tert*-butyl 2-((ethylthio)carbonyl)-5-oxopyrrolidine-1-carboxylate **5** (318 mg, 58% yield) as a colorless oil that solidified upon standing. ^**1**^**H NMR** (500 MHz, CDCl_3_) δ 4.75–4.73 (dd, ^3^*J* = 9.6, 2.5 Hz, 1H, CH), 3.01–2.95 (m, 2H, CH_2_), 2.73–2.66 (m, 1H, CH_2_), 2.54–2.48 (m, 1H, CH_2_), 2.39–2.32 (m, 1H, CH_2_), 2.09–2.03 (m, 1H, CH_2_), 1.49 (s, 9H, (CH_3_)_3_CO), 1.33–1.30 (t, ^3^*J* = 7.4 Hz, 3H, CH_3_) ppm. ^**13**^**C**{^1^H} **NMR** (125 MHz, CDCl_3_) δ 199.4, 173.6, 149.1, 84.0, 65.5, 31.0, 28.0, 23.4, 22.5, 14.8 ppm. All spectroscopic data were consistent with those previously reported.^37^

A solution of *tert*-Butyl 2-((ethylthio)carbonyl)-5-oxopyrrolidine-1-carboxylate (137 mg, 0.5 mmol) and 10% Pd/C (2.2 mg, 0.02 mmol) in acetone (1.5 mL) was cooled to 0 °C. Et_3_SiH (0.24 mL, 1.5 mmol) was added dropwise, and the reaction was allowed to warm to room temperature. After 20 min, the mixture was filtered through Celite and washed with EtOAc/Hexanes (1:1). Volatiles were removed *in vacuo* without heating to afford compound **7** as pale-yellow oil that was immediately used in the next step without further purification. Crude **7** was redissolved in CH_2_Cl_2_ (1.5 mL) and cooled to 0 °C. TFA (0.37 mL, 4.83 mmol) was added dropwise, and the reaction was allowed to warm to room temperature. After 1.5 h, the volatiles were removed *in vacuo* without heating to afford compound **1u** as a pale-yellow oil that was immediately used in the next step without further purification.

#### 6-Acetyl-5-methyl-1,2-dihydro-3*H*-pyrrolizin-3-one (**6**)

The prepared solution of 5-oxopyrrolidine-2-carbaldehyde **1u** in THF (0.4 mL) was added to a mixture of acetylacetone **2a** (0.031 mL, 0.300 mmol) and ZrOCl_2_·8H_2_O (9.7 mg, 0.030 mmol) in H_2_O (0.2 mL), and the solution was allowed to stir at room temperature for 6 h. Extractive work-up afforded compound **6** as a yellow oil (28% yield over three steps) without further purification. ^**1**^**H NMR** (700 MHz, CDCl_3_) δ 6.20 (s, 1H, Pyrrole-H), 3.02 (m, 2H, CH_2_CH_2_), 2.97 (m, 2H, CH_2_CH_2_), 2.76 (s, 3H, CH_3_), 2.40 (s, 3H, CH_3_) ppm. ^**13**^**C**{^1^H} **NMR** (175 MHz, CDCl_3_) δ 196.0, 174.1, 137.2, 130.6, 129.0, 104.2, 35.1, 29.4, 18.5, 11.3 ppm. **IR** (film) 3107, 2940 w, 1754 s, 1660 s, 1531 m, 1400 s, 1204 s cm^–1^. **HRMS (ESI)***m*/*z*: [M + H]^+^ calcd for C_10_H_12_O_2_N, 178.0862; found, 178.0861.

### Attempted Synthesis and Conversion of Knoevenagel Intermediate

Following a modified version of the procedure outlined in previous literature,^38^ a stirred solution of Boc-Phe-H **1a** (50 mg, 200 mmol) and acetylacetone **2a** (22 mg, 0.220 mmol) in anhydrous CH_2_Cl_2_ (0.4 mL) was treated with piperidine (0.016 mmol) and acetic acid (0.016 mmol) at 0 °C. After 45 min, a small amount of 3 Å molecular sieves was added. The reaction was then stirred at room temperature. Extractive work-up and purification on a neutral alumina column (20% Ethyl Acetate/Hexanes) gave 25 mg of crude aldol addition product **8** (as a diastereomeric mixture). The product was treated with ZrOCl_2_·8H_2_O (2.6 mg, 0.008 mmol) in 0.2 mL of THF and 0.1 mL of H_2_O and stirred at room temperature for 6 h to yield crude pyrrole product, *tert*-butyl 3-acetyl-2-methyl-5-phenyl-1*H*-pyrrole-1-carboxylate (**3aa**).

## Data Availability

The data underlying this study are available in the published article and its Supporting Information.

## Supporting Information Available

Optimization tables for the various approaches, ^1^H NMR spectra for known compounds, NMR (^1^H and ^13^C) spectra for all new compounds, and HPLC data for **3ra** (PDF)
