# Supplementary material for: Zr-Catalyzed Synthesis of Tetrasubstituted 1,3-Diacylpyrroles from N-Acyl α-Aminoaldehydes and 1,3-Dicarbonyls
Source: J Org Chem. 2023 Jun 9;88(13):8915–28. doi: 10.1021/acs.joc.3c00675 (PMC10337043; doi:10.1021/acs.joc.3c00675)
Supplement: Supplementary file 1 — jo3c00675_si_001.pdf [file jo3c00675_si_001.pdf]

Supporting Information for

Zr-Catalyzed Synthesis of Tetrasubstituted 1,3-Diacylpyrroles from N-Acyl α-Aminoaldehydes and 1,3-Dicarbonyls

Caria Evans[a,b], William J. Berkey[a,b], Christopher W. Jones[b,c,]* and Stefan France[a,b,]*

[a] School of Chemistry and Biochemistry, Georgia Institute of Technology, Atlanta, Georgia 30332, United States

[b] Renewable Bioproducts Institute, Georgie Institute of Technology, Atlanta, Georgia 30332, United States

[c] School of Chemical & Biomolecular Engineering, Georgia Institute of Technology, Atlanta, Georgia 30332, United States

christopher.jones@chbe.gatech.edu
stefan.france@chemistry.gatech.edu

Table of Contents

1. Reaction Optimization Tables

A. Optimization of Conditions for Zr-Catalyzed Pyrrole Synthesis....S-4
B. Optimization of Conditions for Boc-Trp(Boc)-H....S-6

2. Copies of NMR Spectra

^{1}H NMR of Compound **1a**....S-7
^{1}H NMR of Compound **1b**....S-8
^{1}H NMR of Compound **1c**....S-9
^{1}H NMR of Compound **1d**....S-10
^{1}H NMR of Compound **1e**....S-11
^{1}H NMR of Compound **1f**....S-12
^{1}H NMR of Compound **1g**....S-13
^{1}H NMR of Compound **1h**....S-14
^{1}H NMR of Compound **1i**....S-15
^{1}H NMR of Compound **1j**....S-16
^{1}H NMR of Compound **1k**....S-17
^{1}H NMR of Compound **1l**....S-18
^{1}H NMR of Compound **1m**....S-19
^{1}H NMR of Compound **1n**....S-20
^{1}H NMR of Compound **1o**....S-21
^{1}H NMR of Compound **1p**....S-22
^{1}H NMR of Compound **1q**....S-23
^{1}H NMR of Compound **N-Boc-Val-Phe-OMe**....S-24

^{1}H NMR of Compound **N-Boc-Val-Phe-OH**……S-25
^{1}H NMR of Compound **1r**……S-26
^{1}H NMR of Compound **3aa**……S-27
^{13}C NMR of Compound **3aa**……S-28
^{1}H NMR of Compound **3ba**……S-29
^{13}C NMR of Compound **3ba**……S-30
^{1}H NMR of Compound **3ca**……S-31
^{13}C NMR of Compound **3ca**……S-32
^{1}H NMR of Compound **3da**……S-33
^{13}C NMR of Compound **3da**……S-34
^{1}H NMR of Compound **3ea**……S-35
^{13}C NMR of Compound **3ea**……S-36
^{1}H NMR of Compound **3fa**……S-37
^{13}C NMR of Compound **3fa**……S-38
^{1}H NMR of Compound **3ga**……S-39
^{13}C NMR of Compound **3ga**……S-40
^{1}H NMR of Compound **3ha**……S-41
^{13}C NMR of Compound **3ha**……S-42
^{1}H NMR of Compound **3ia**……S-43
^{13}C NMR of Compound **3ia**……S-44
^{1}H NMR of Compound **3ja**……S-45
^{13}C NMR of Compound **3ja**……S-46
^{1}H NMR of Compound **3la**……S-47
^{13}C NMR of Compound **3la**……S-48
^{1}H NMR of Compound **3ma**……S-49
^{13}C NMR of Compound **3ma**……S-50
^{1}H NMR of Compound **3na**……S-51
^{13}C NMR of Compound **3na**……S-52
^{1}H NMR of Compound **3oa**……S-53
^{13}C NMR of Compound **3oa**……S-54
^{1}H NMR of Compound **3pa**……S-55
^{13}C NMR of Compound **3pa**……S-56
^{1}H NMR of Compound **3ra**……S-57
^{13}C NMR of Compound **3ra**……S-58
^{1}H NMR of Compound **3ab**……S-59
^{13}C NMR of Compound **3ab**……S-60
^{1}H NMR of Compound **3ac**……S-61
^{13}C NMR of Compound **3ac**……S-62
^{1}H NMR of Compound **3ad**……S-63
^{13}C NMR of Compound **3ad**……S-64
^{1}H NMR of Compound **3ae**……S-65
^{13}C NMR of Compound **3ae**……S-66
^{1}H NMR of Mixture of **3ta and 4ta**……S-67
^{13}C NMR of Compound **3aa**……S-68

^{1}H NMR of Compound **7**……………………………………………………………………………………….S-69
^{1}H NMR of Compound **5**……………………………………………………………………………………….S-70
^{1}H NMR of Compound of **6**…………………………………………………………………………………….S-71
^{13}C NMR of Compound of **6**……………………………………………………………………………………S-72
Crude ^{1}H NMR of Compound **8**…………………………………………………………………………….S-73
Crude ^{1}H NMR of Reaction of Crude **8** with $ZrOCl_2•8H_2O$………………………………………….S-74

3. **HPLC data for 3ra**………………………………………………………………………………………………S-75

1. Reaction Optimization Tables

A. Optimization of Conditions for Zr-Catalyzed Pyrrole Synthesis

Table S1. Reaction Optimization of Boc-Phe-H (**1a**) and Acetylacetone (**2a**)

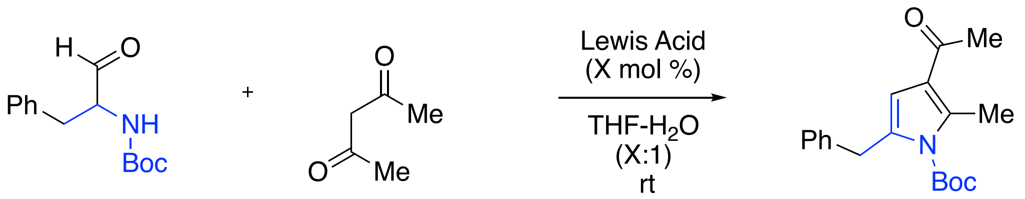

entry	Lewis acid	Lewis acid (mol %)	Time (hr.)	(1a:2a)[a]	Solvent (X M)	yield of 3aa (%)[b]
1	none	–	24	1:1	THF/Water (2:1)	8
2	$Sc(OTf)_3$	15	24	1:1	THF/Water (2:1)	79
3	$CeCl_3$	15	24	1:1	THF/Water (2:1)	36
4	$ZrCl_4$	15	24	1:1	THF/Water (2:1)	57
5	$ZrOCl_2$* $8H_2O$	15	24	1:1	THF/Water (2:1)	68
6	$ZrOCl_2$* $8H_2O$	2.5	6	1:1	THF/Water (2:1)	37
7	$ZrOCl_2$* $8H_2O$	5	6	1:1	THF/Water (2:1)	48
8	$ZrOCl_2$* $8H_2O$	10	6	1:1	THF/Water (2:1)	85
9	$ZrOCl_2$* $8H_2O$	15	6	1:1	THF/Water (2:1)	70
10	$ZrOCl_2$* $8H_2O$	10	3	1:1	THF/Water (2:1)	58
11	$ZrOCl_2$* $8H_2O$	15	3	1:1	THF/Water (2:1)	58
12	$Sc(OTf)_3$	15	6	1:1	THF/Water (2:1)	70
13	$ZrOCl_2$* $8H_2O$	15	6	1:1	THF/Water (2:1)	70
14	$Sc(OTf)_3$	10	6	1:1	THF/Water (2:1)	77
15	$ZrOCl_2$* $8H_2O$	10	6	1:1	THF/Water (2:1)	85
16	$CeCl_3$	10	6	1:1	THF/Water (2:1)	13
17	$Yb(OTf)_3$	10	6	1:1	THF/Water (2:1)	38

18	$Bi(OTf)_3$	10	6	1:1	THF/Water (2:1)	46
19	$Y(OTf)_3$	10	6	1:1	THF/Water (2:1)	62
20	$Ga(OTf)_3$	10	6	1:1	THF/Water (2:1)	33
21	$Zn(OTf)_2$	10	6	1:1	THF/Water (2:1)	33
22	$ZrCl_4$	10	6	1:1	THF/Water (2:1)	62
23	$ZrOCl_2$* $8H_2O$	10	6	1.5:1	THF/Water (2:1)	68
24	$ZrOCl_2$* $8H_2O$	10	6	2:1	THF/Water (2:1)	77
25	$ZrOCl_2$* $8H_2O$	10	6	1:1.5	THF/Water (2:1)	40
26	$ZrOCl_2$* $8H_2O$	10	6	1:1(dropwise)	THF/Water (2:1)	88[c]
27	$ZrOCl_2$* $8H_2O$	10	6	1:1(dropwise)	THF/Water (3:1) (0.75 M)	56[c]
28	$ZrOCl_2$* $8H_2O$	10	6	1:1(dropwise)	THF/Water (4:1) (1.25 M)	45[c]
29	$ZrOCl_2$* $8H_2O$	10	6	1:1(dropwise)	2-MeTHF/Water (2:1) (0.5 M)	5[c]
30	$ZrOCl_2$* $8H_2O$	10	6	1:1(dropwise)	Water only (0.5 M)	NR[d]

[a] Reactions performed on a 0.300 mmol scale with Boc-Phe-H **1a**, acetylacetone **2**, and Lewis acid (X mol %) in THF:H_2O (X:1 v/v) at the indicated ratio, temperature, and time. [b] Yields of **3aa** reported as quantitative NMR yields using dimethyl terephthalate as internal standard. [c] α-Aminoaldehyde was dissolved in THF and added dropwise to stirring solution of **2a**, $ZrOCl_2$*$8H_2O$ and H_2O. [d] NR = No Reaction. Only starting material was recovered.

B. Optimization of Conditions for Boc-Trp(Boc)-H

Table S2. Optimization of Boc-Trp(Boc)-H (**1e**) and Acetylacetone (**2a**)[a]

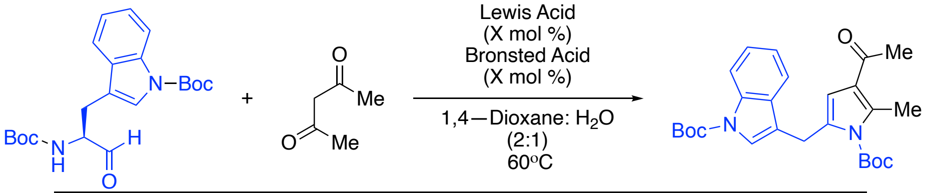

entry	Lewis Acid	Bronsted Acid	Yield [b]
1	$ZrOCl_2$*8H_2O (10 mol%)	Amberlyst-15 (10 mol%)	59%
2	$ZrCl_4$ (10 mol%)	Amberlyst-15 (10 mol%)	36%
3	-	Amberlyst-15 (10 mol%)	5%
4	$ZrOCl_2$*8H_2O (10 mol%)	Acetic Acid (10 mol%)	30%
5	$ZrOCl_2$*8H_2O (10 mol%)	TFA (10 mol%)	56%

[a] Reactions performed with Boc-Trp(Boc)-H (0.300 mmol) and acetylacetone (0.300 mmol) at the indicated temperature for 6 h. [b] Yields reported as quantitative NMR yields using dimethyl terephthalate as internal standard.

2. Copies of NMR Spectra

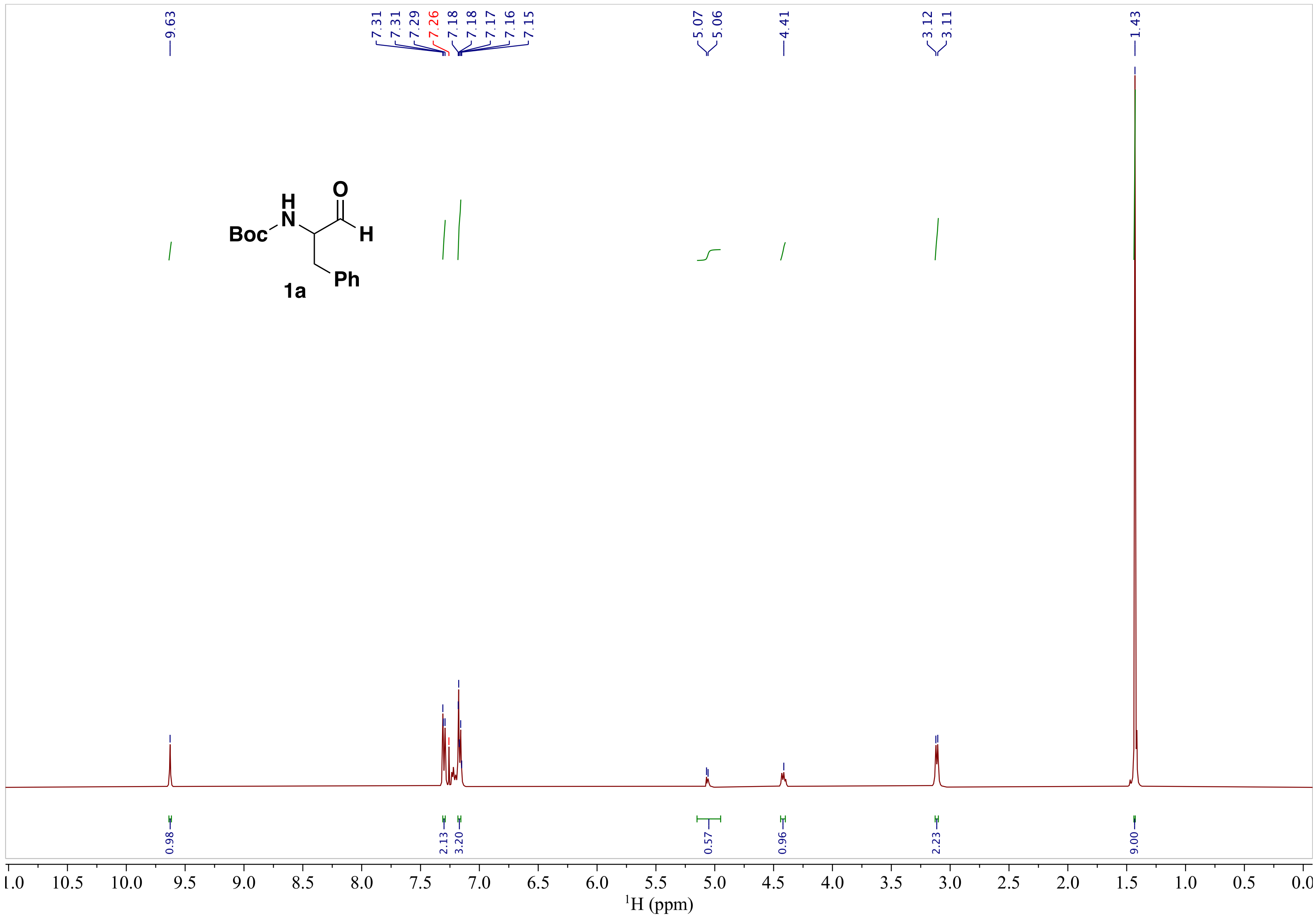

700 MHz, $CDCl_3$

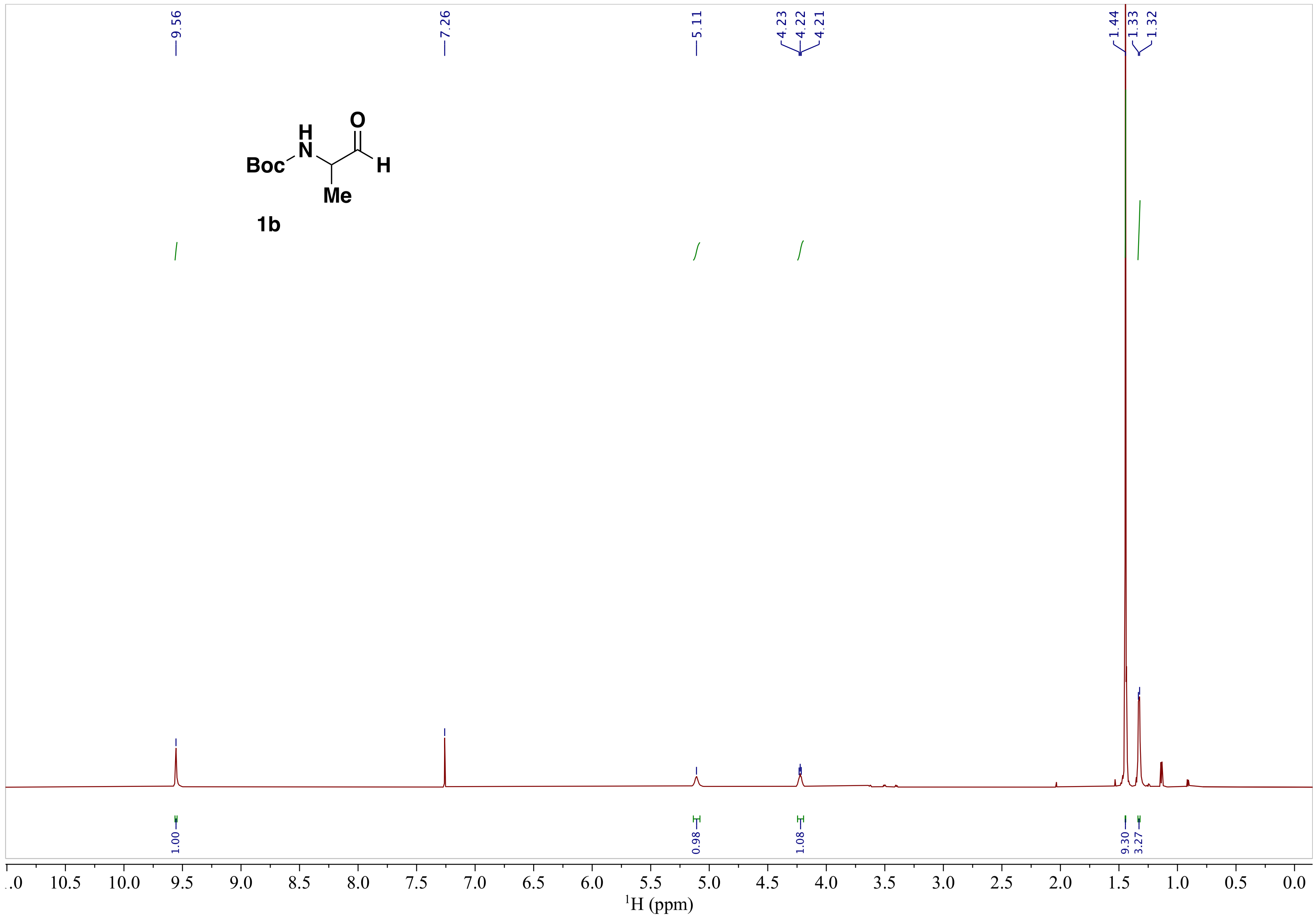

700 MHz, $CDCl_3$

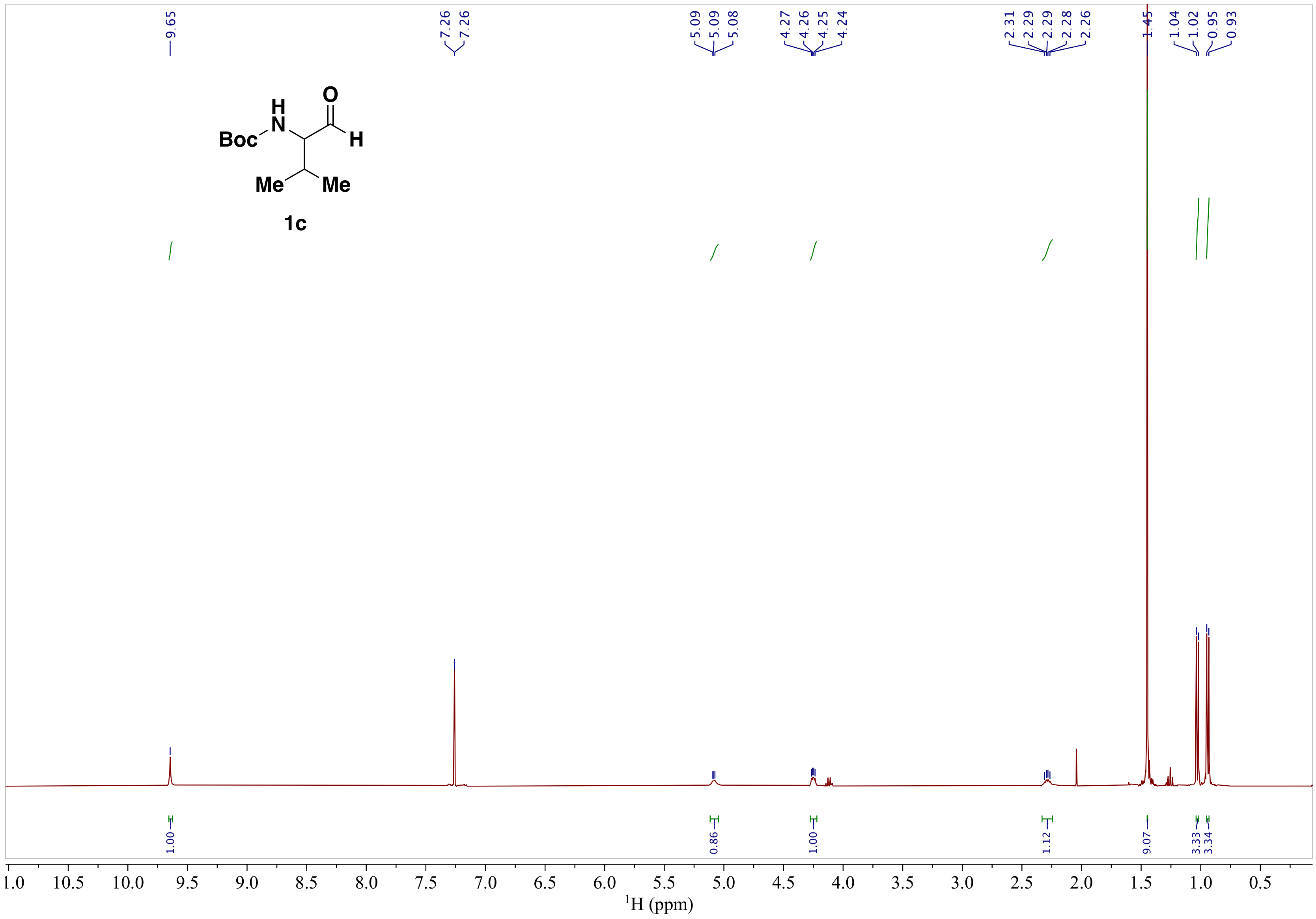

700 MHz, $CDCl_3$

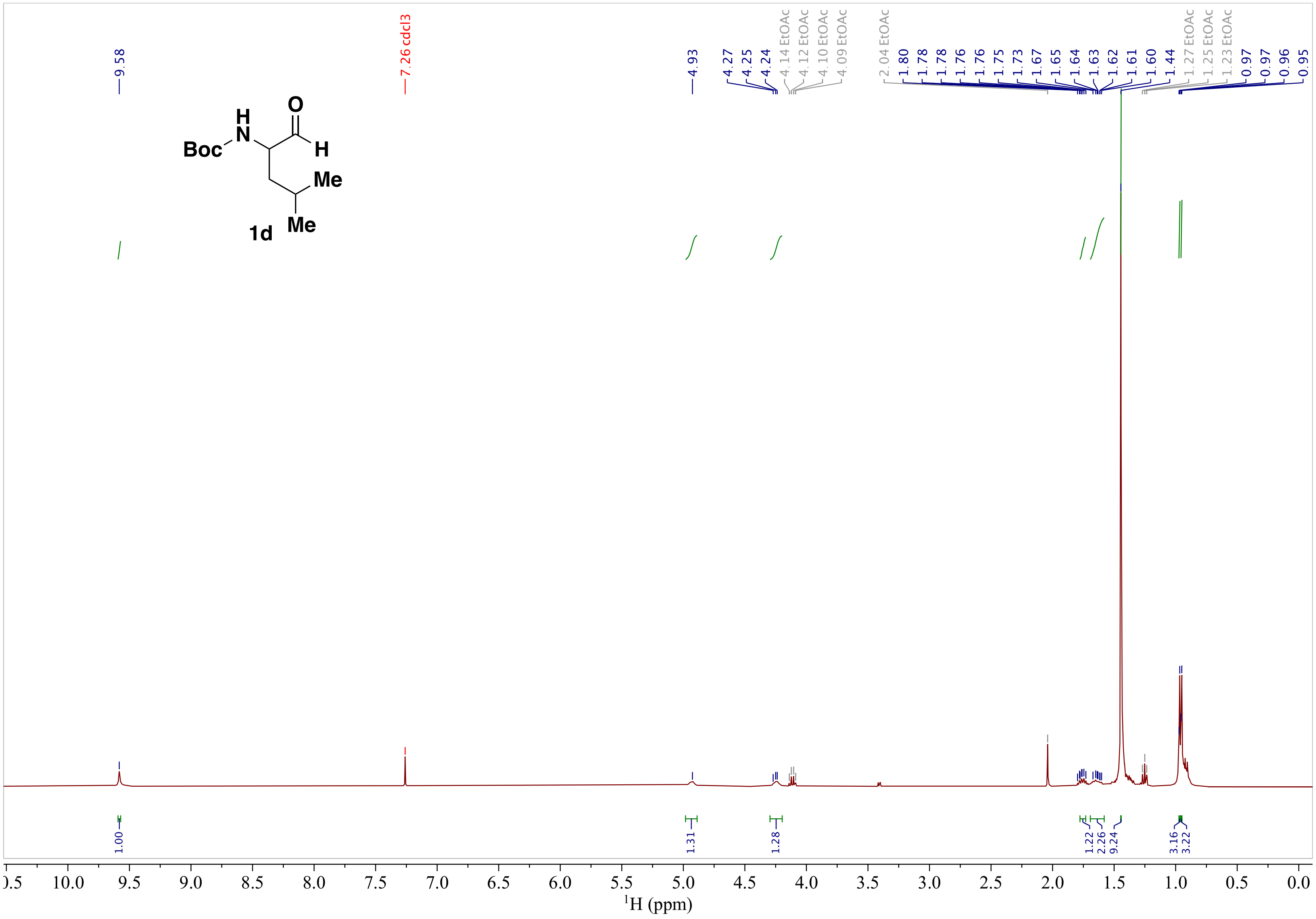

700 MHz, $CDCl_3$

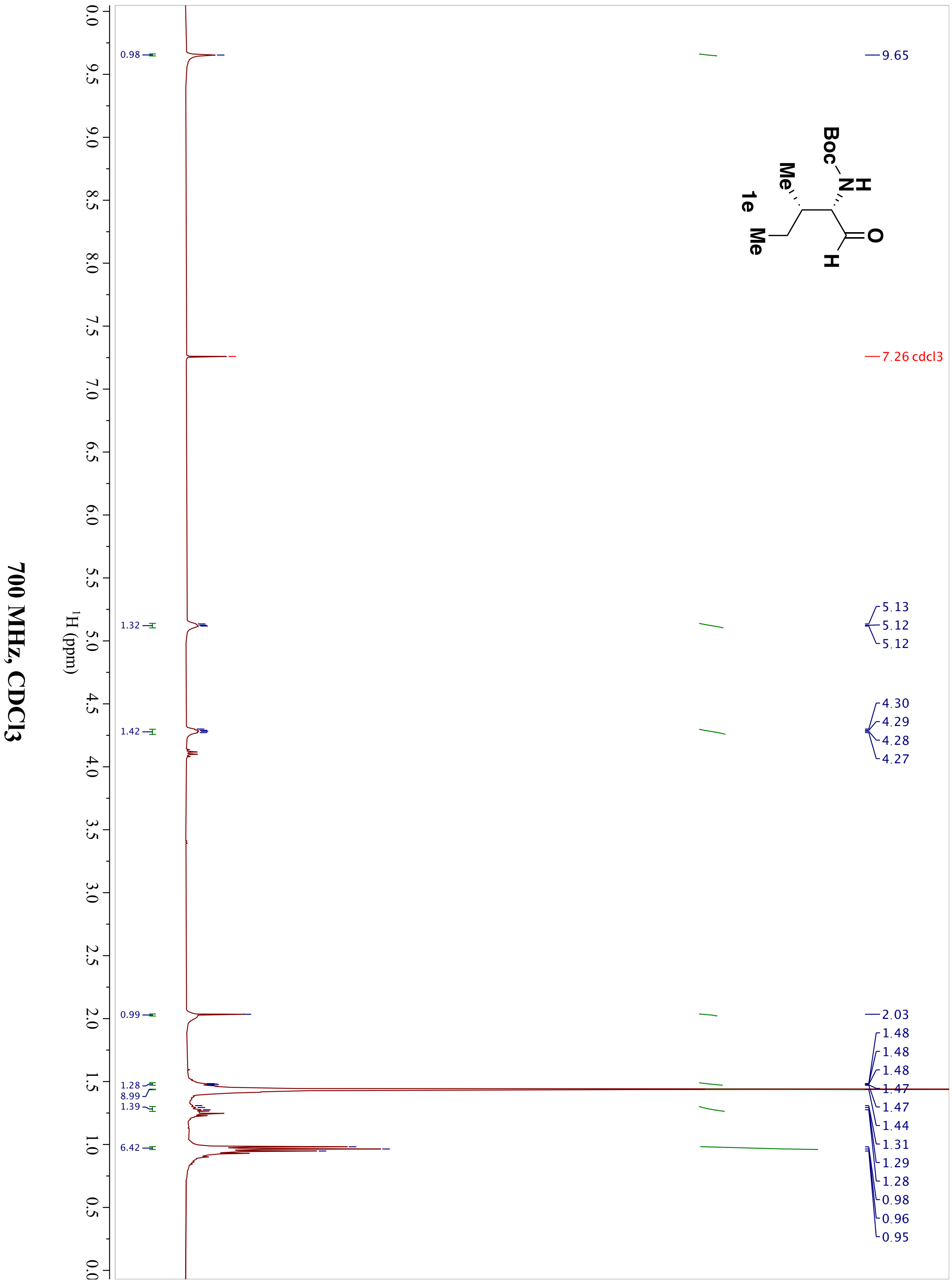

700 MHz, CDCl3
1e
Boc
H
N
O
H
Me
Me
7.26 cdcl3
¹H (ppm)

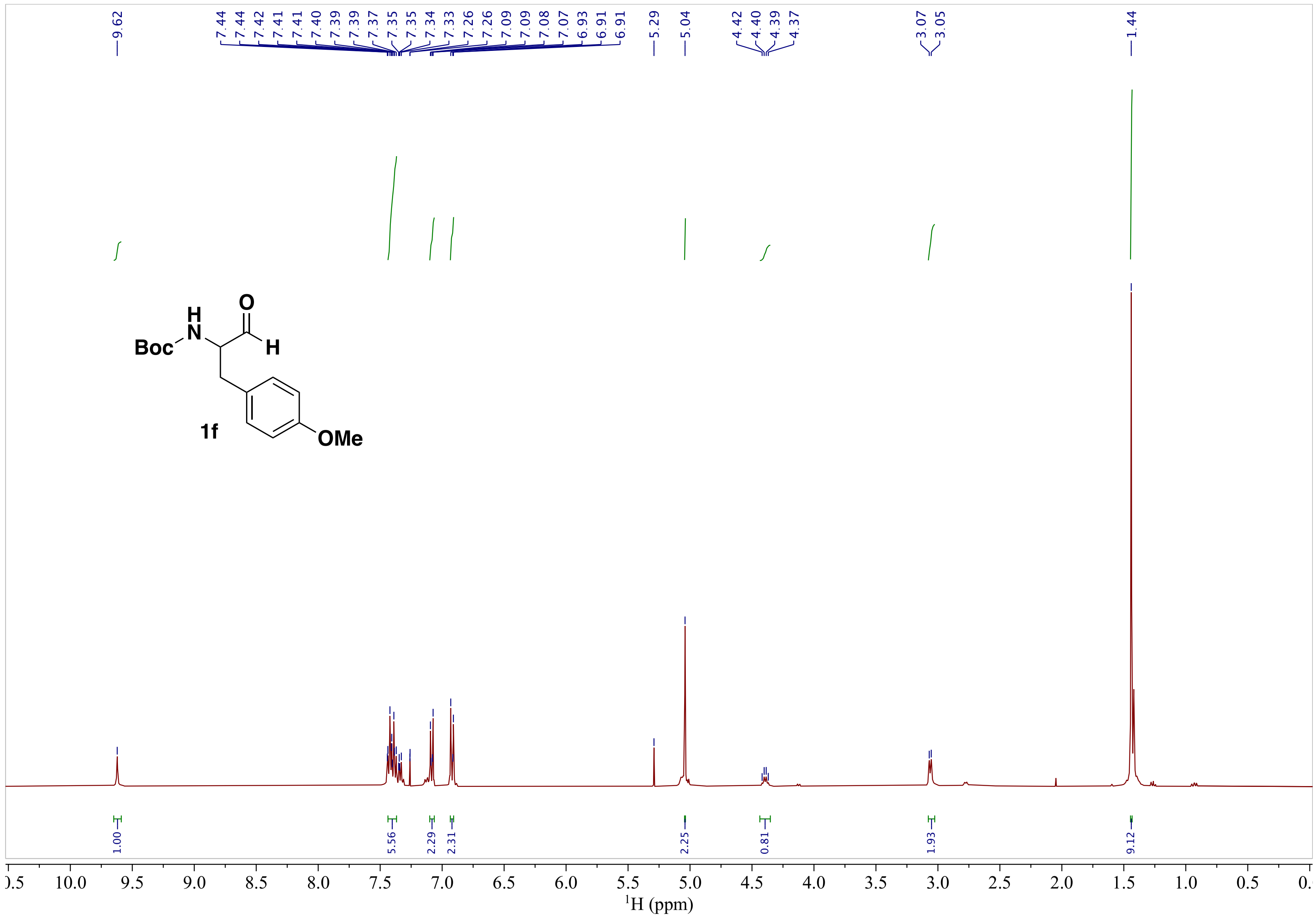

700 MHz, $CDCl_3$

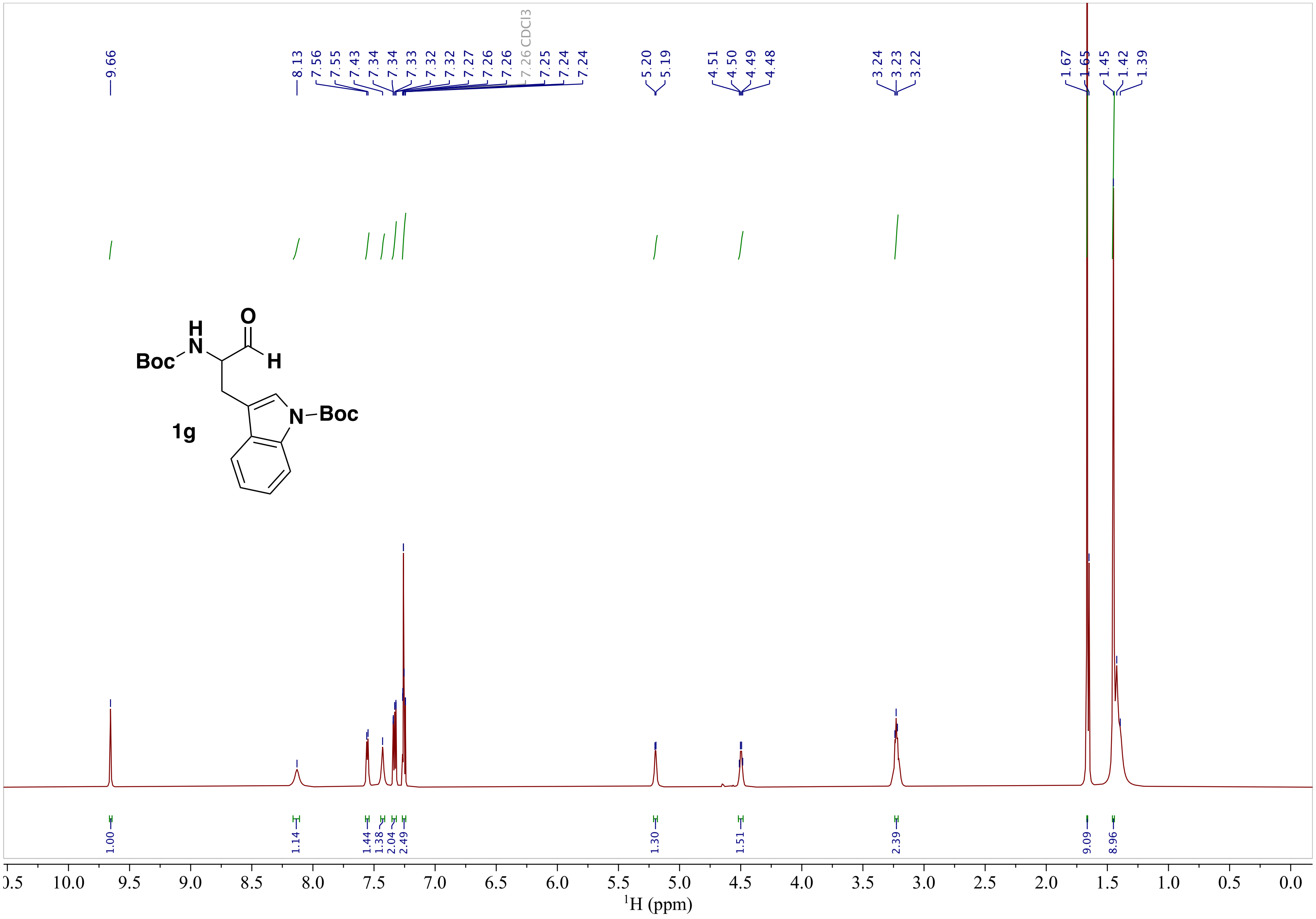

700 MHz, $CDCl_3$

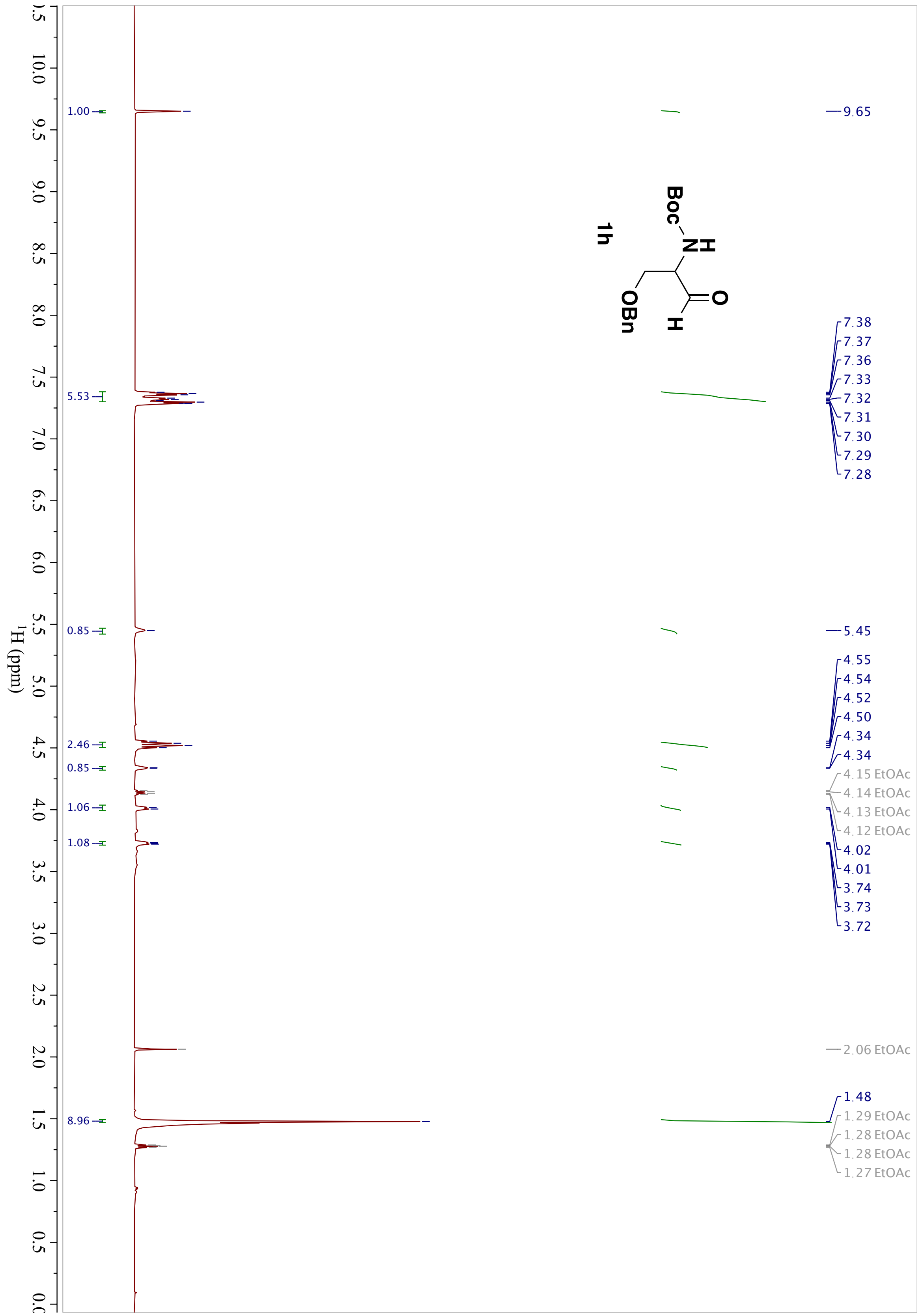

700 MHz, $CDCl_3$

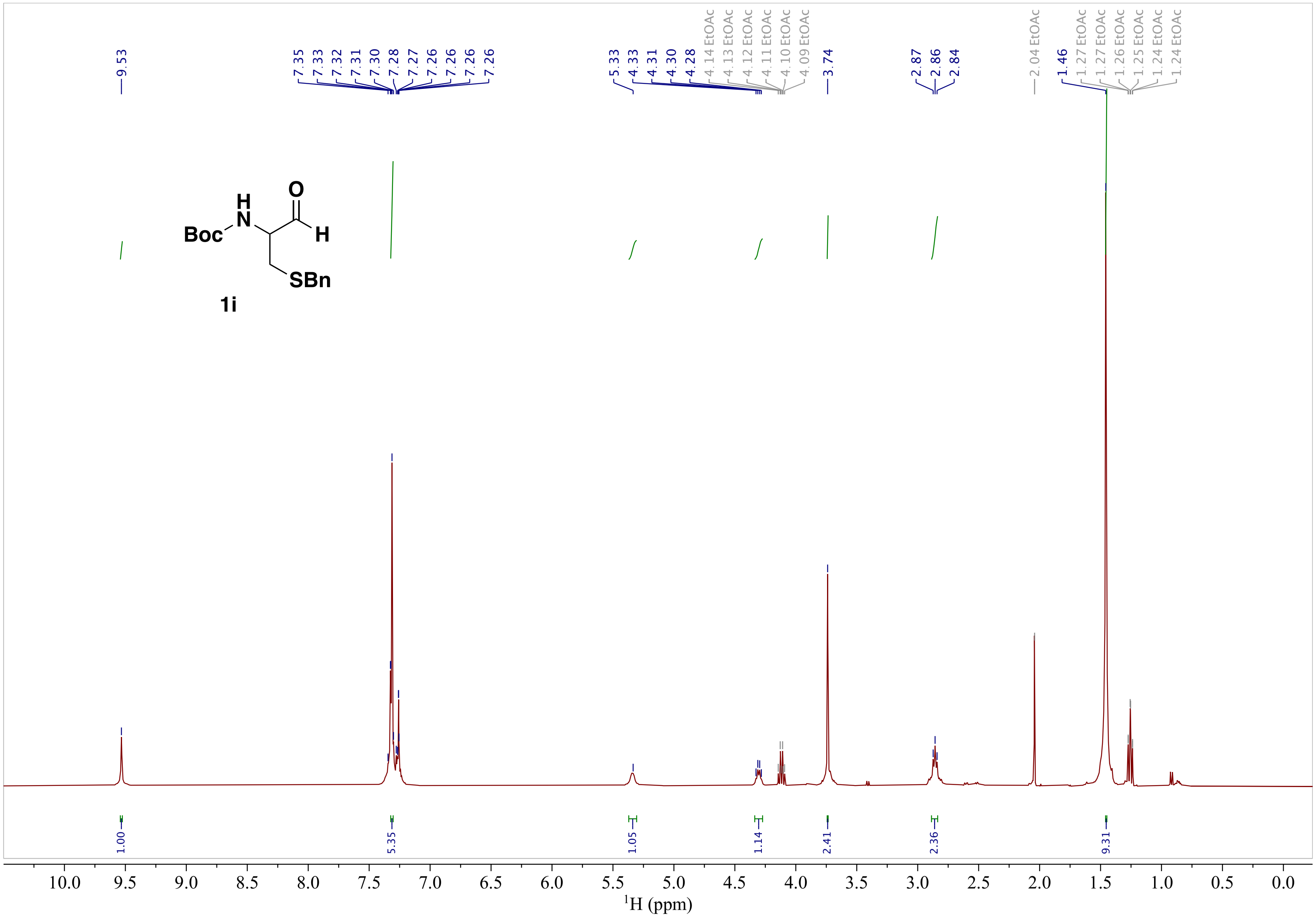

700 MHz, $CDCl_3$

700 MHz, CDCl3

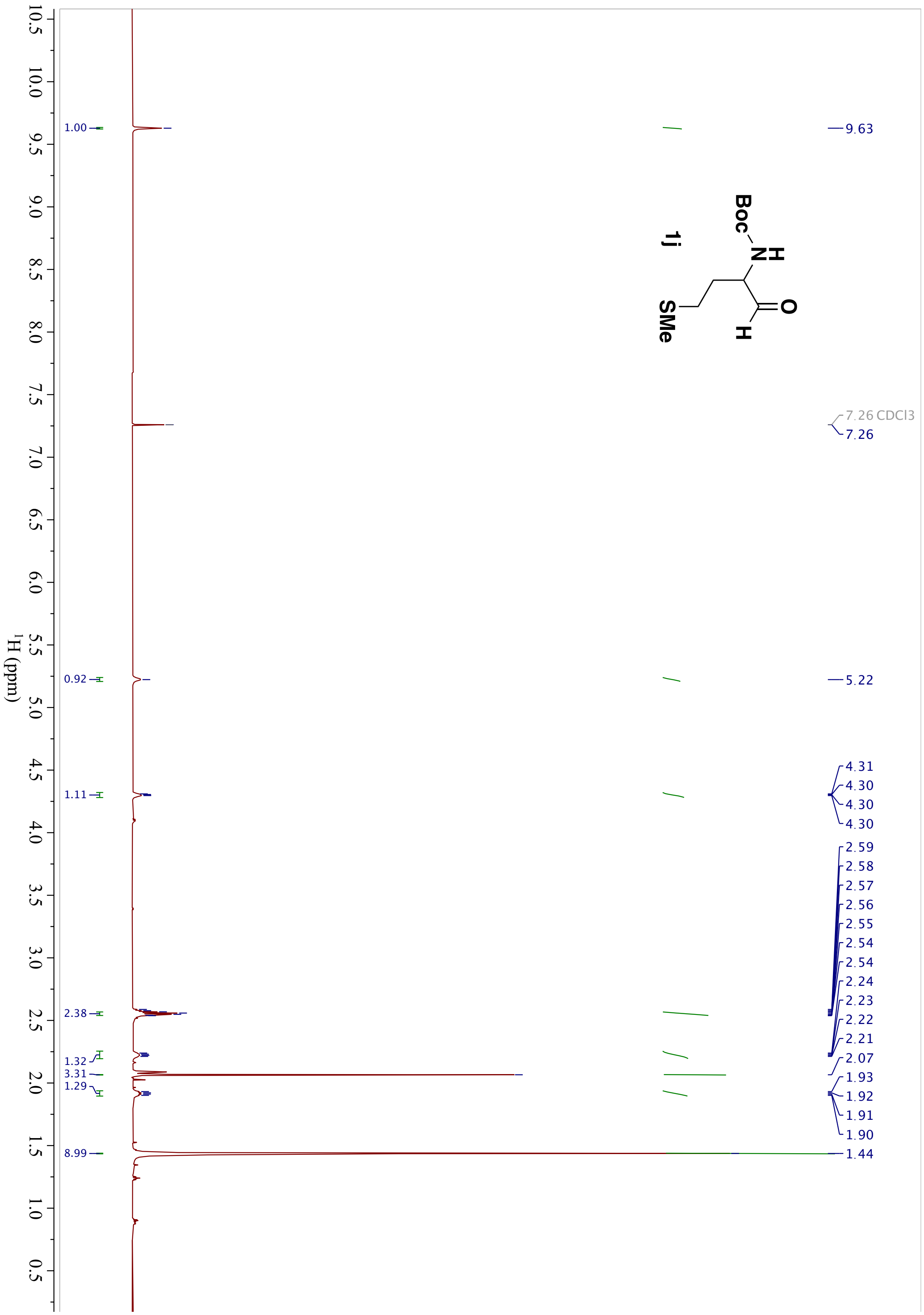

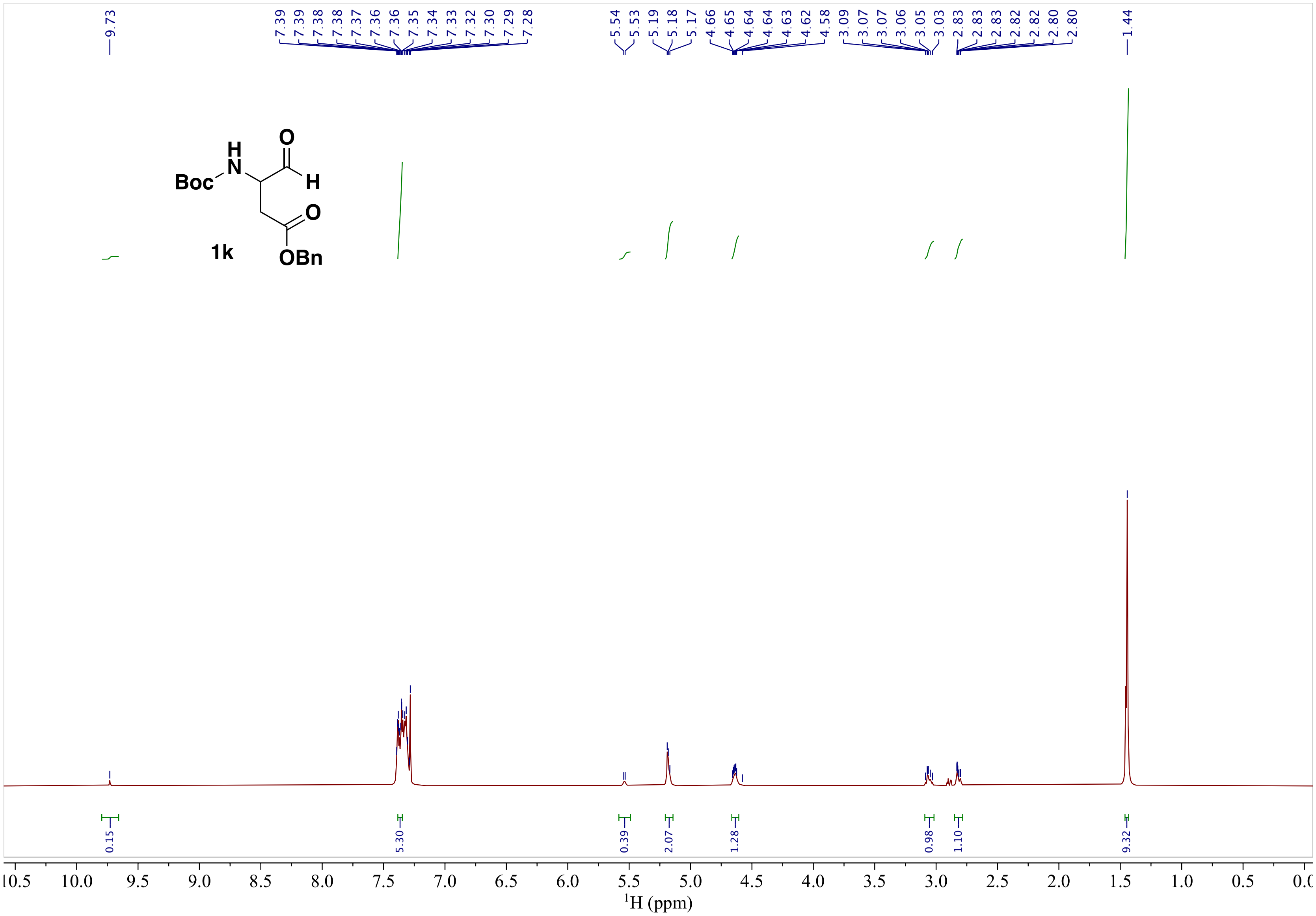

700 MHz, $CDCl_3$

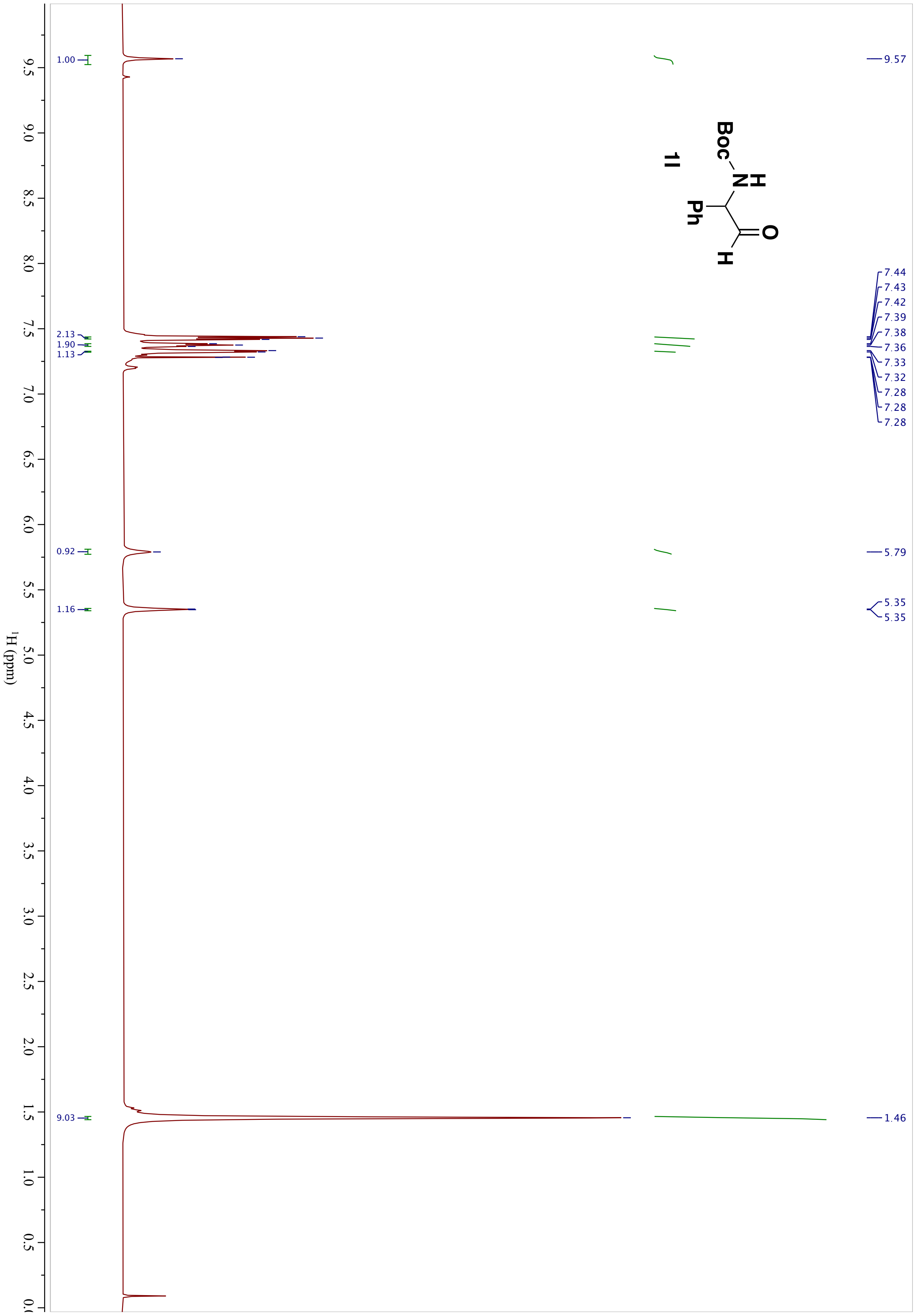

700 MHz, $CDCl_3$

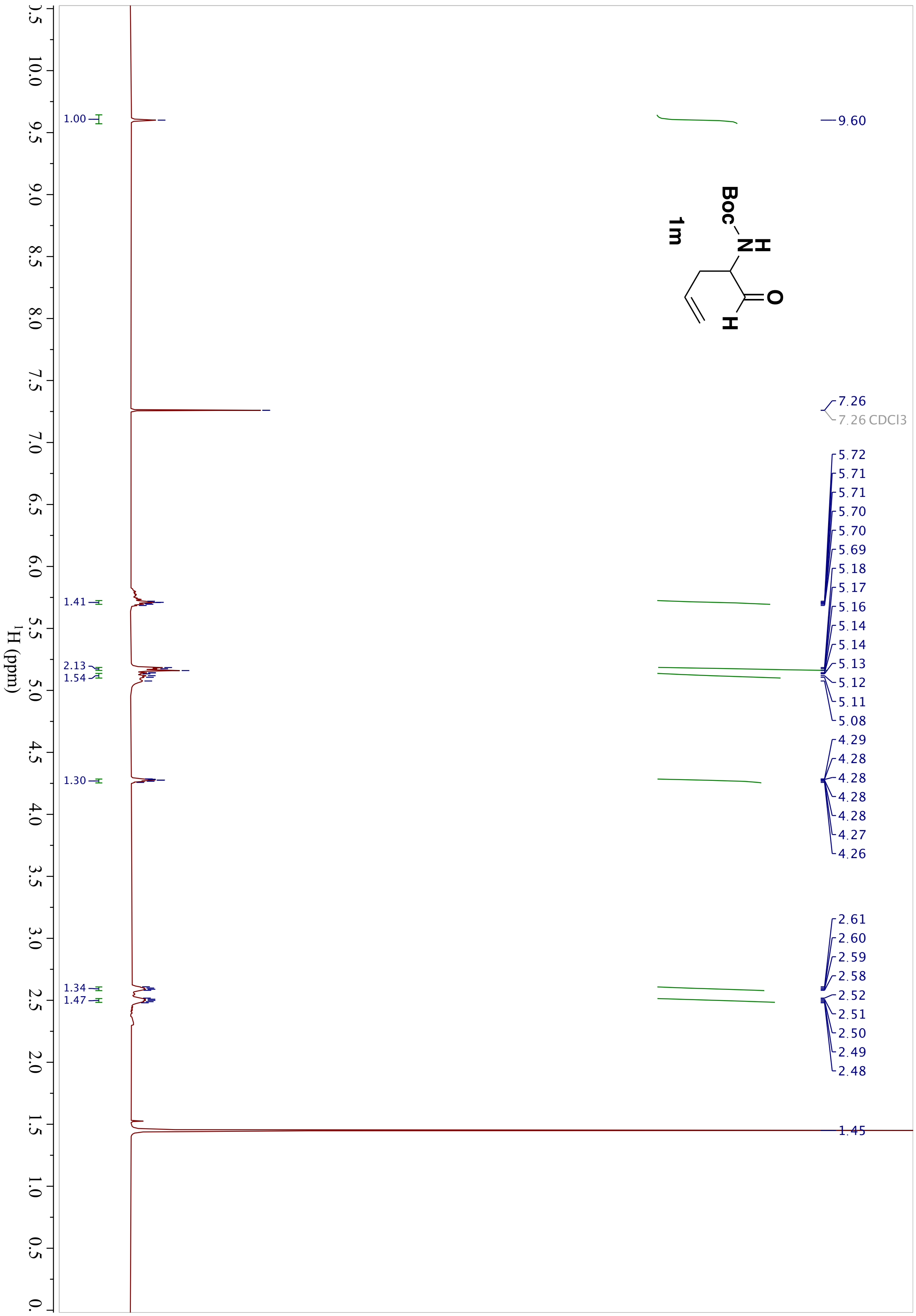

700 MHz, CDCl3

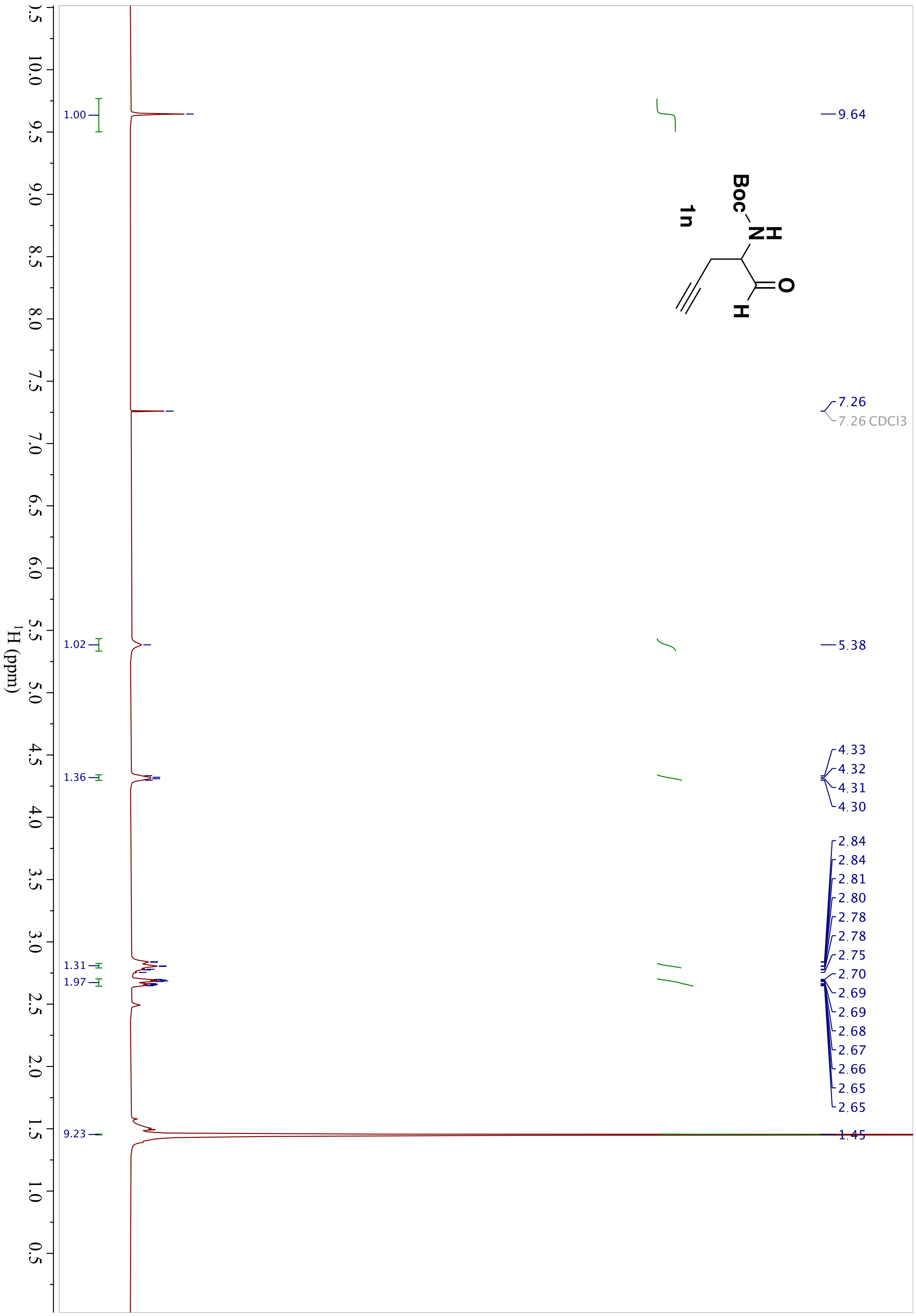

700 MHz, $CDCl_3$

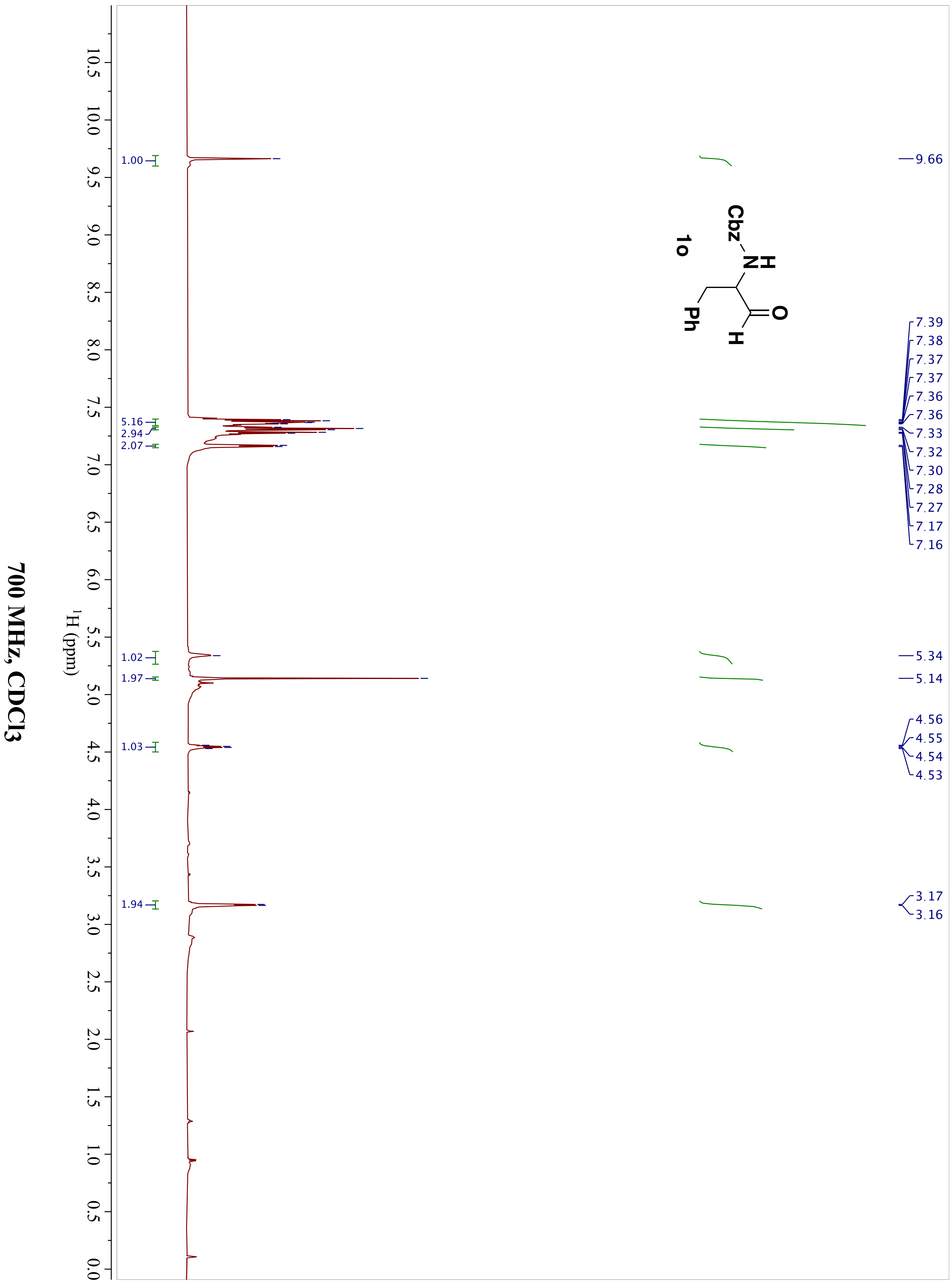
700 MHz, CDCl3
1H (ppm)
1o
Cbz
H
N
O
H
Ph
9.66
7.39
7.38
7.37
7.37
7.36
7.36
7.33
7.32
7.30
7.28
7.27
7.17
7.16
5.34
5.14
4.56
4.55
4.54
4.53
3.17
3.16
1.00
5.16
2.94
2.07
1.02
1.97
1.03
1.94

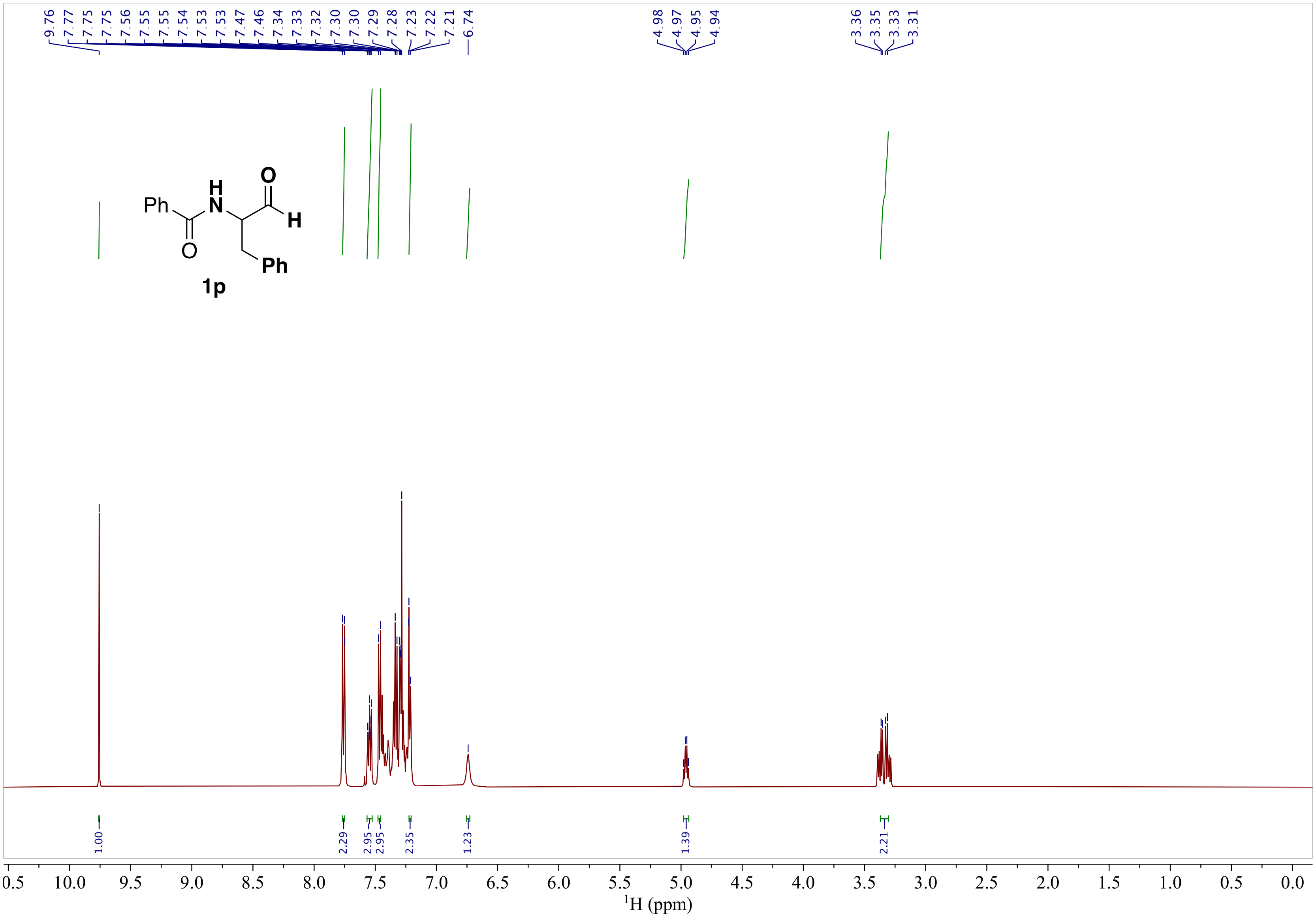

700 MHz, $CDCl_3$

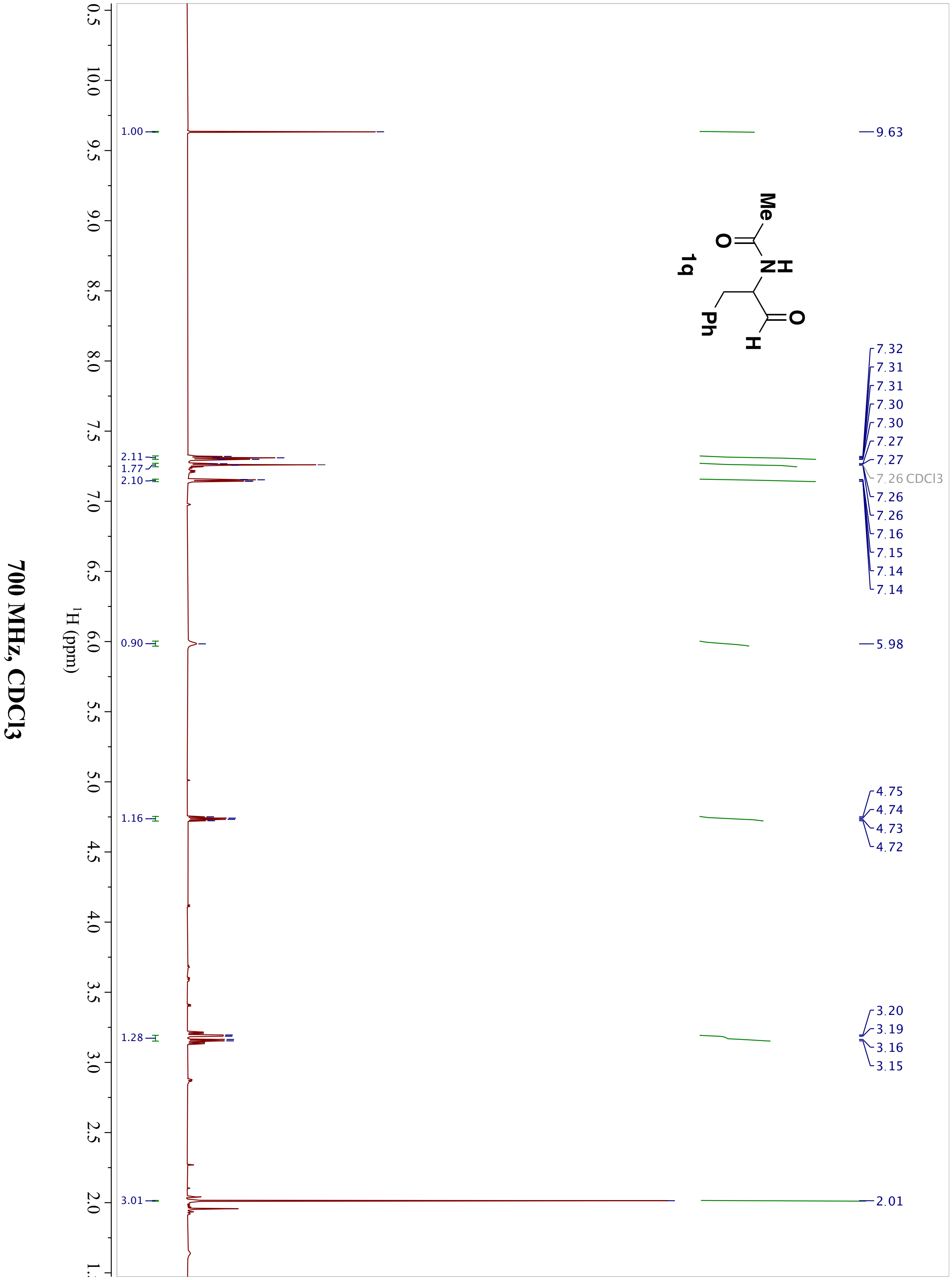

700 MHz, CDCl3
1q
Me
O
H
N
O
H
Ph
9.63
7.32
7.31
7.31
7.30
7.30
7.27
7.27
7.26 CDCl3
7.26
7.26
7.16
7.15
7.14
7.14
5.98
4.75
4.74
4.73
4.72
3.20
3.19
3.16
3.15
2.01
1.00
2.11
1.77
2.10
0.90
1.16
1.28
3.01
^{1}H (ppm)

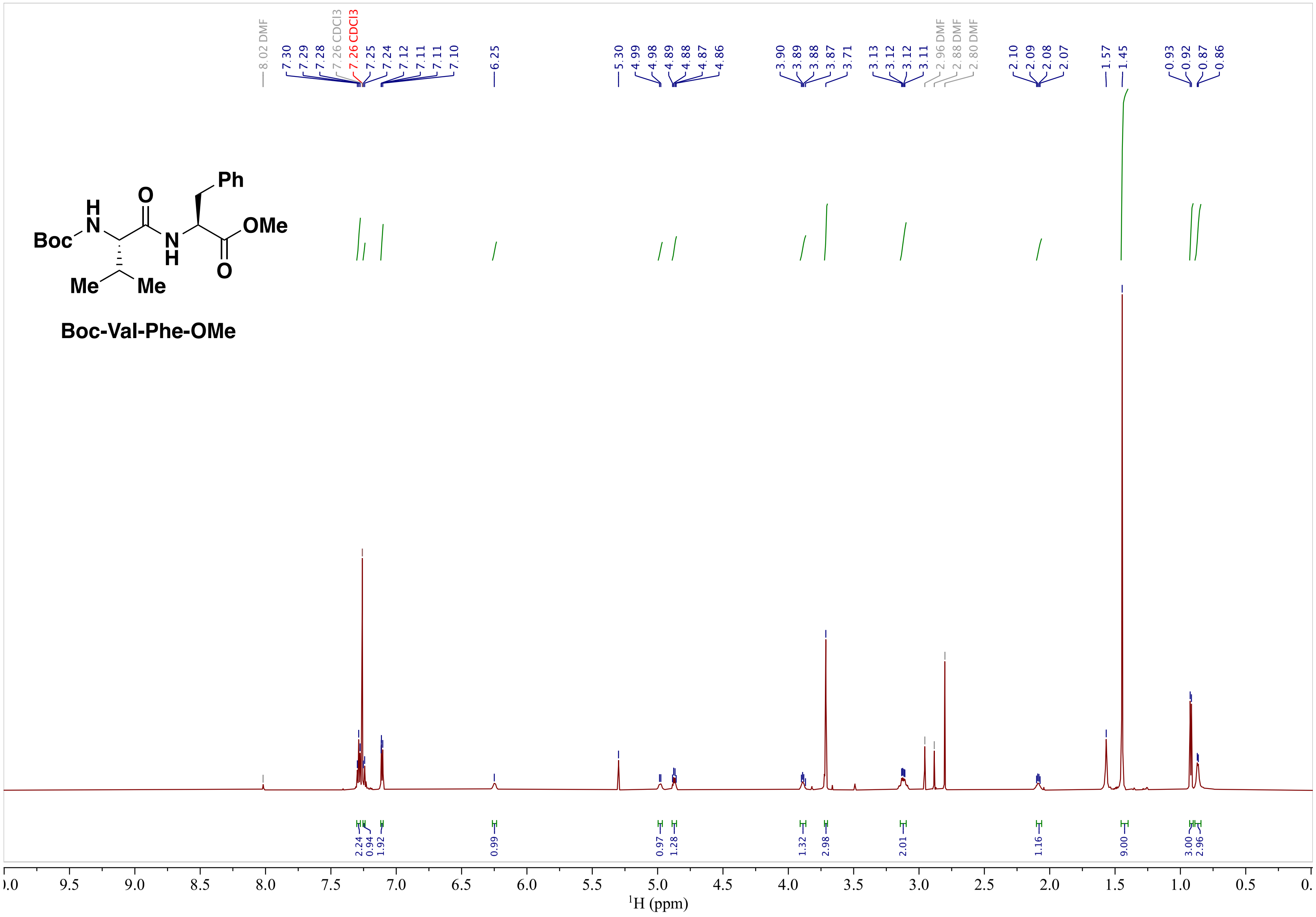

700 MHz, $CDCl_3$

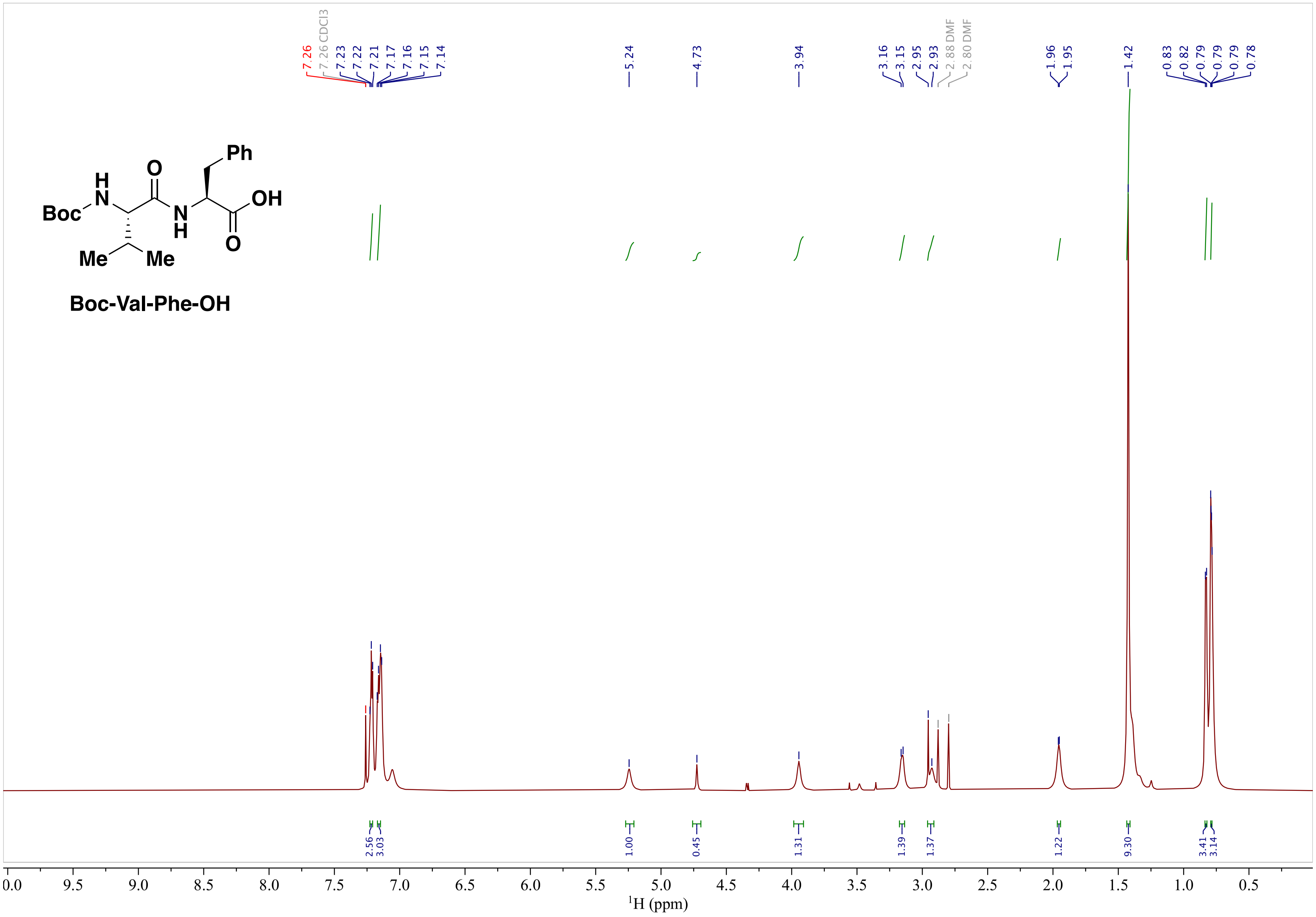

700 MHz, $CDCl_3$

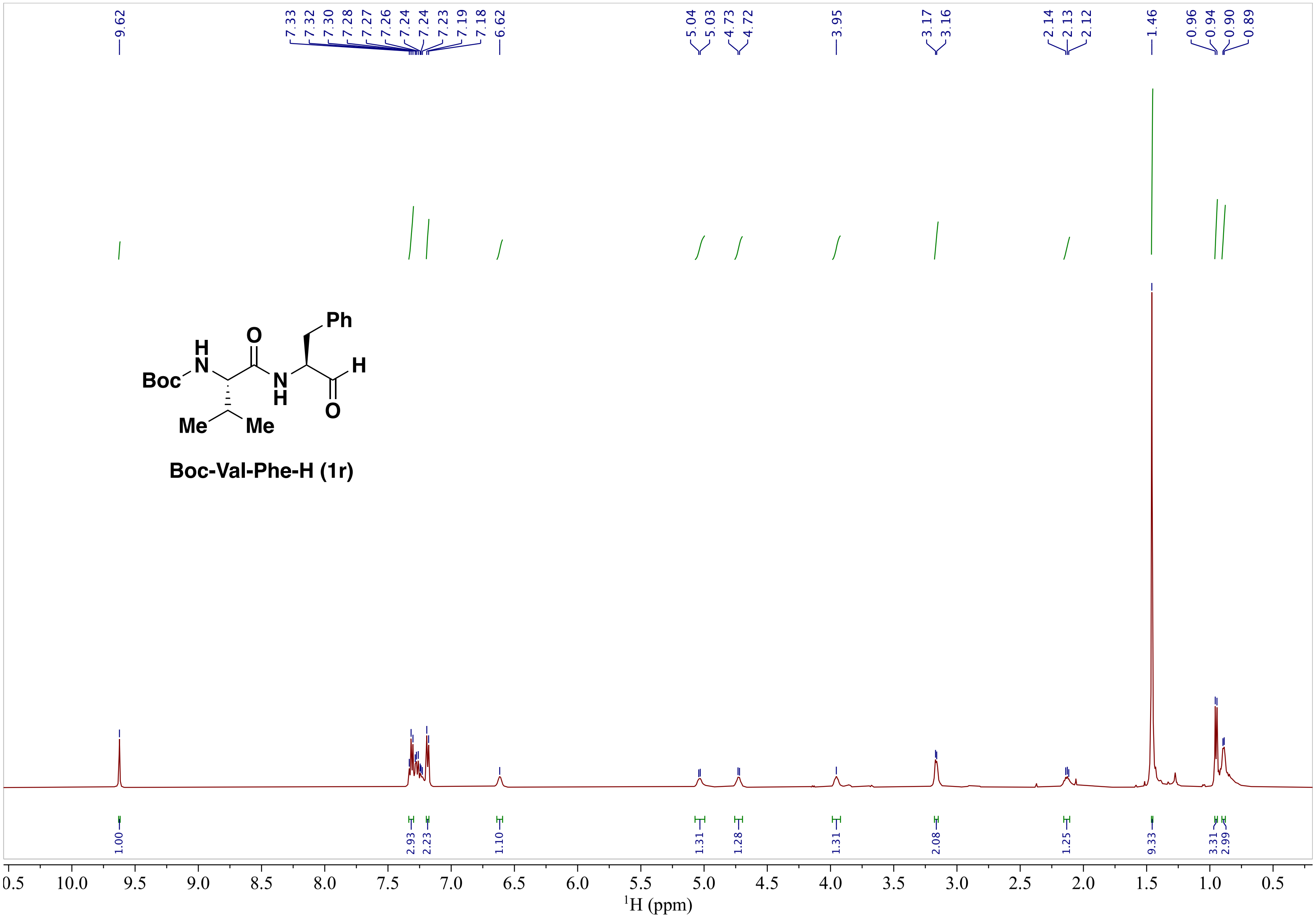

700 MHz, $CDCl_3$

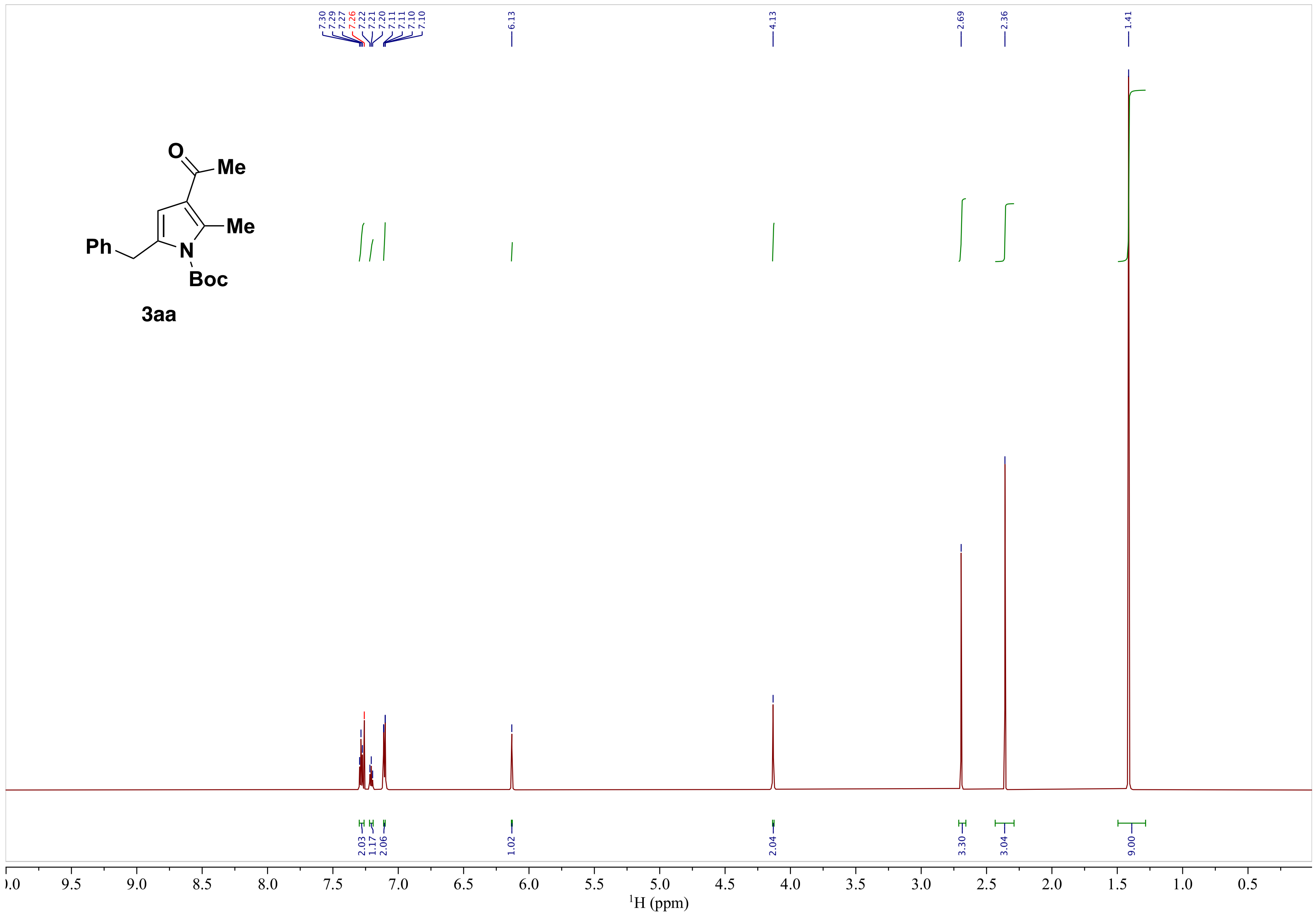

700 MHz, $CDCl_3$

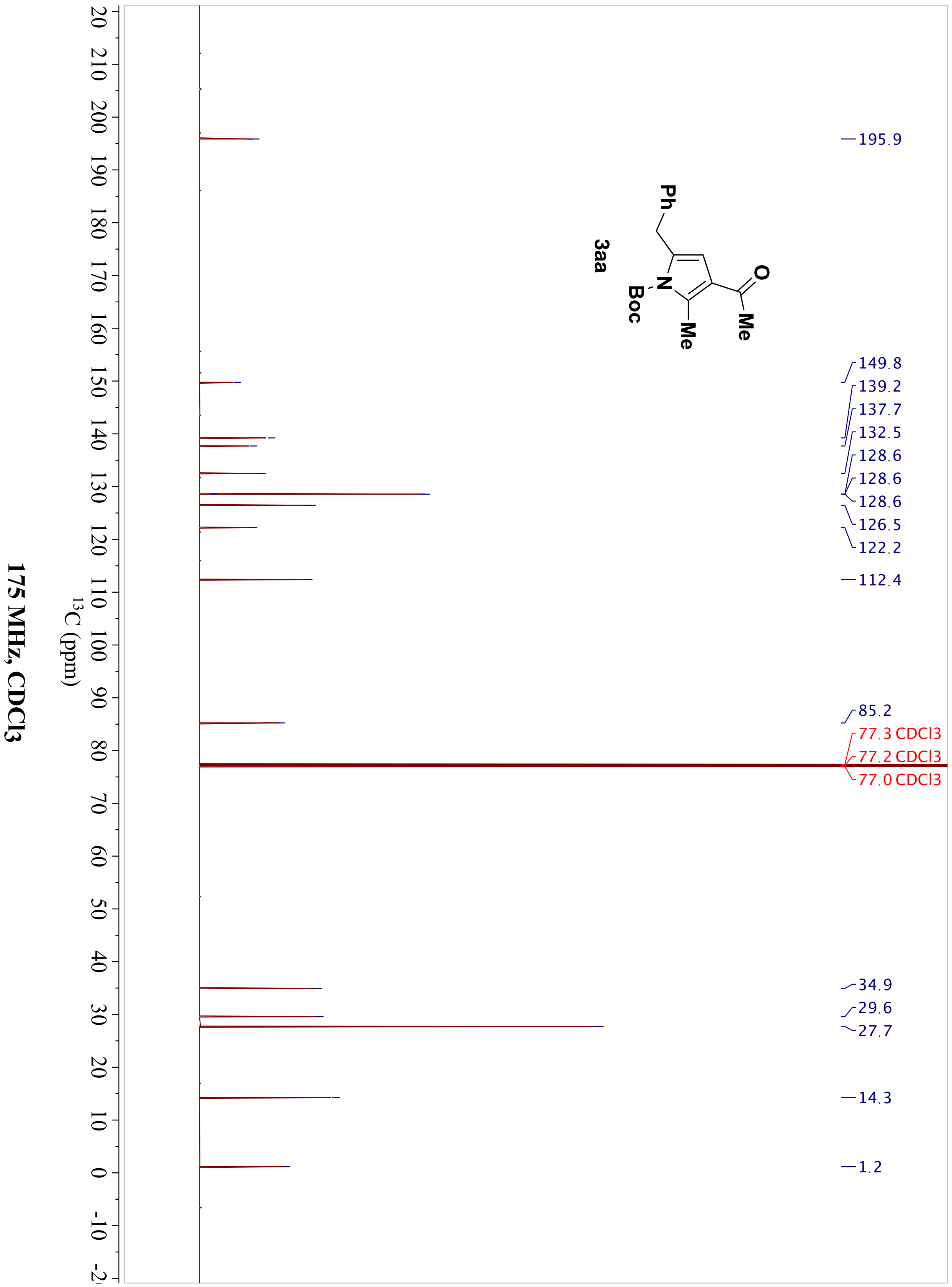
3aa
195.9
149.8
139.2
137.7
132.5
128.6
128.6
128.6
126.5
122.2
112.4
85.2
77.3 CDCl3
77.2 CDCl3
77.0 CDCl3
34.9
29.6
27.7
14.3
1.2
^{13}C (ppm)
175 MHz, CDCl3

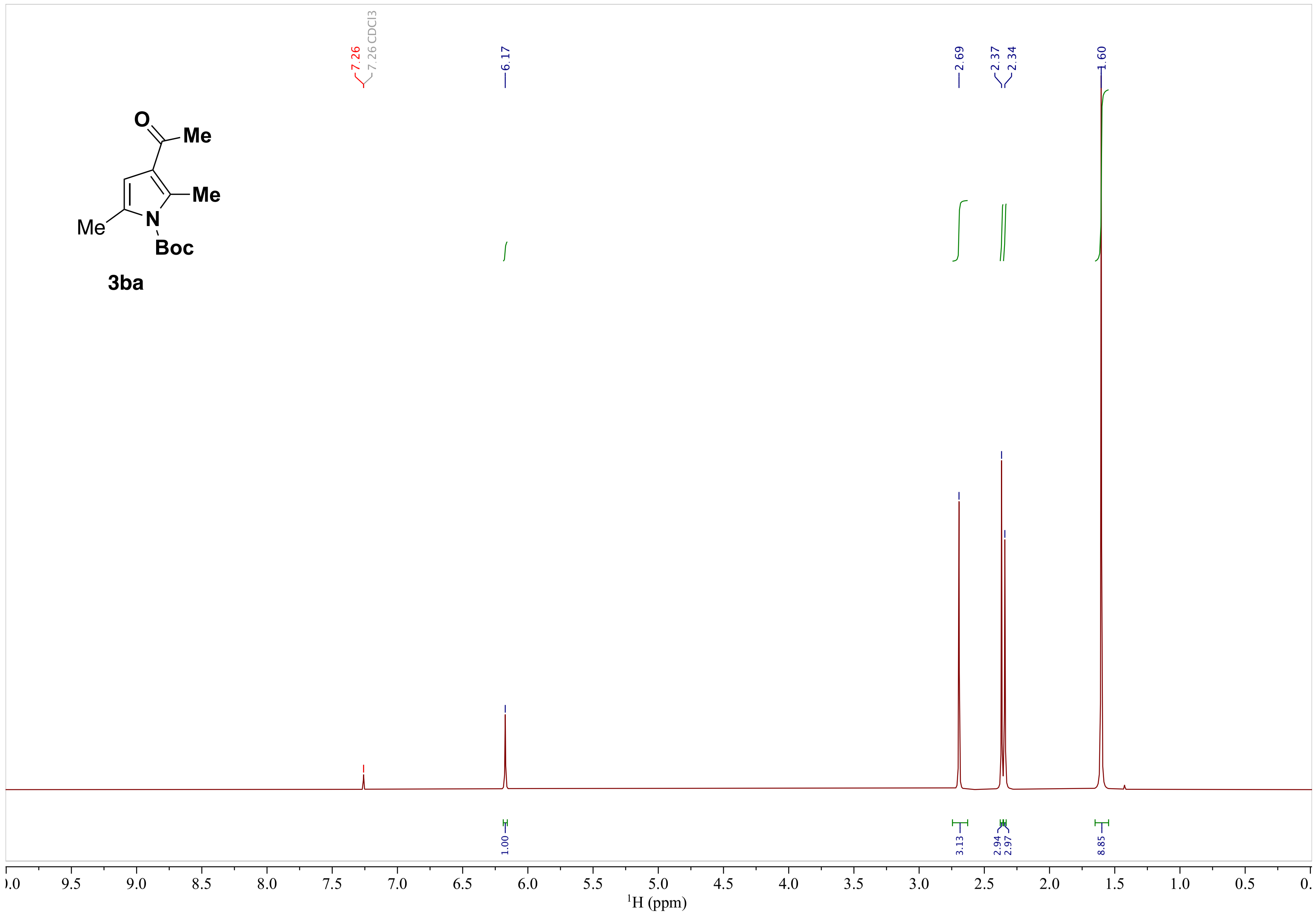

3ba

700 MHz, CDCl3

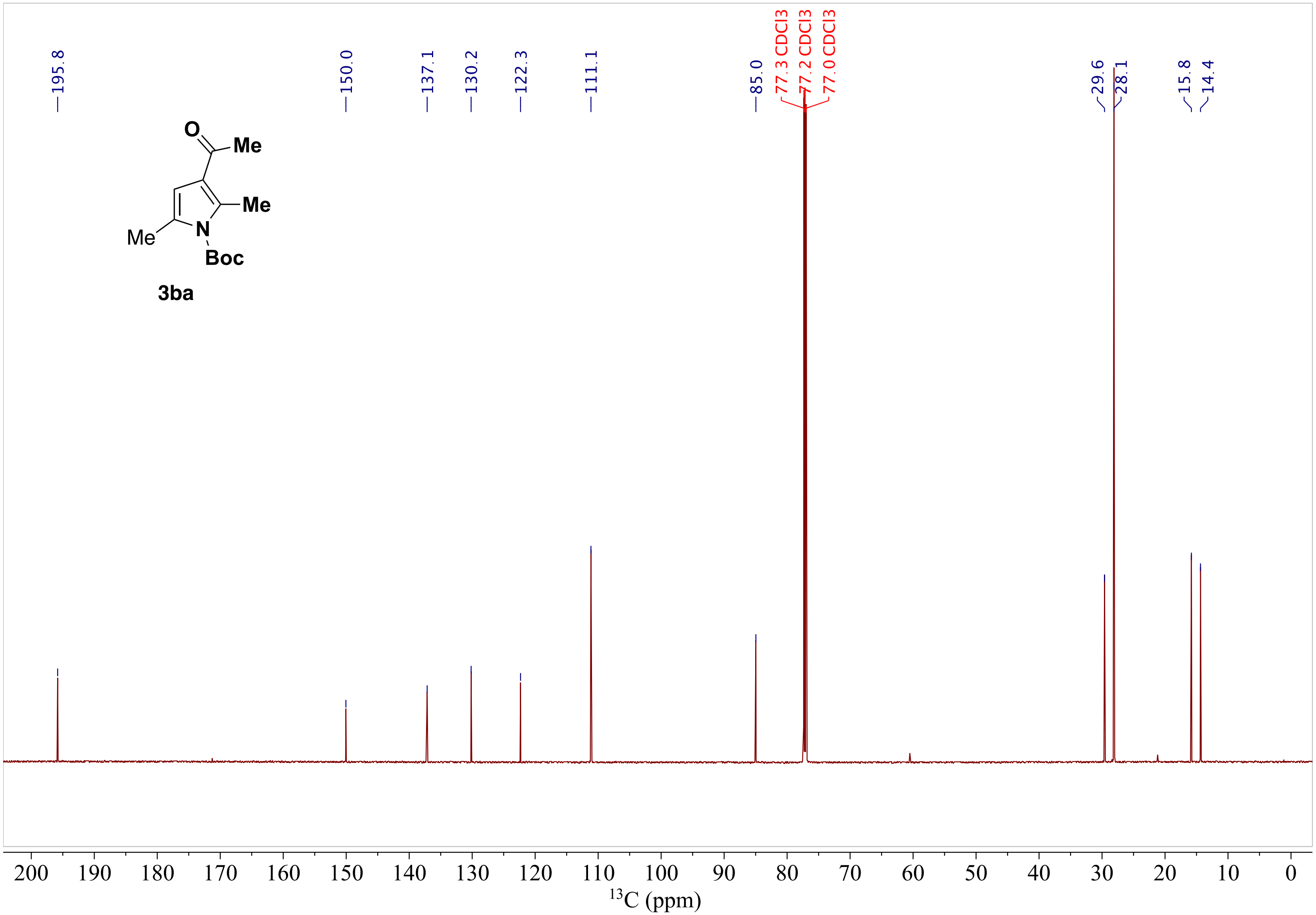

175 MHz, $CDCl_3$

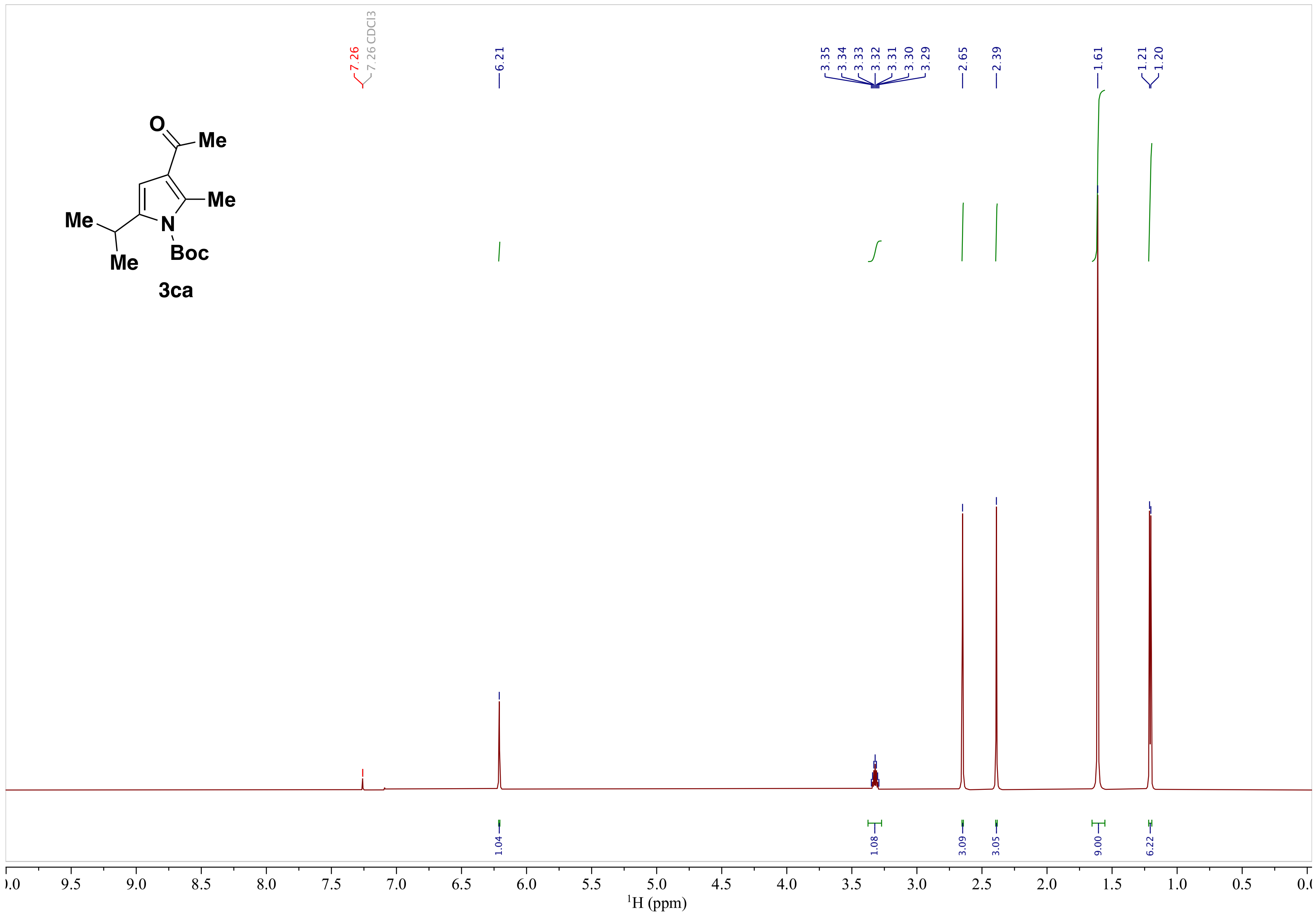

700 MHz, $CDCl_3$

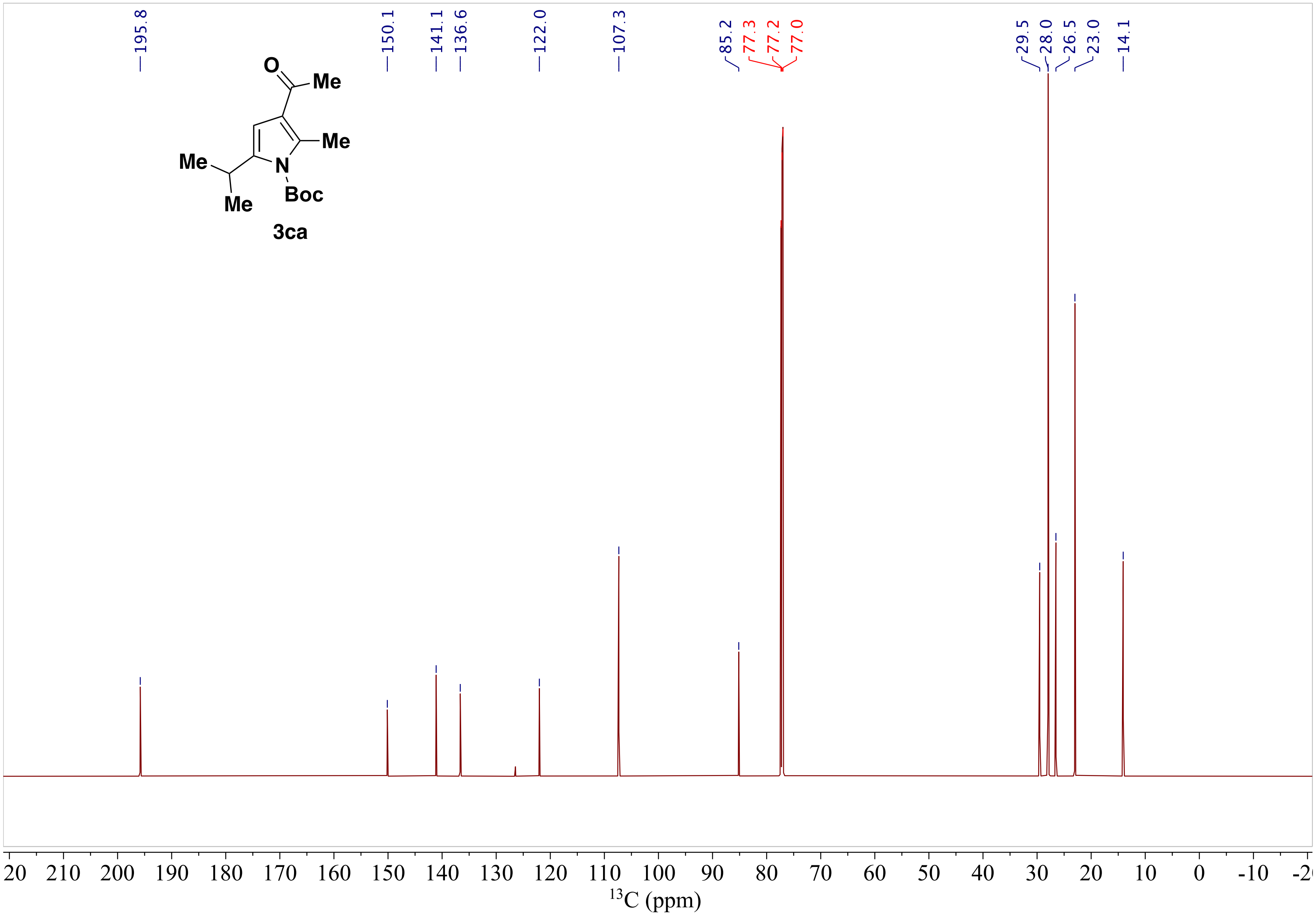

175 MHz, $CDCl_3$

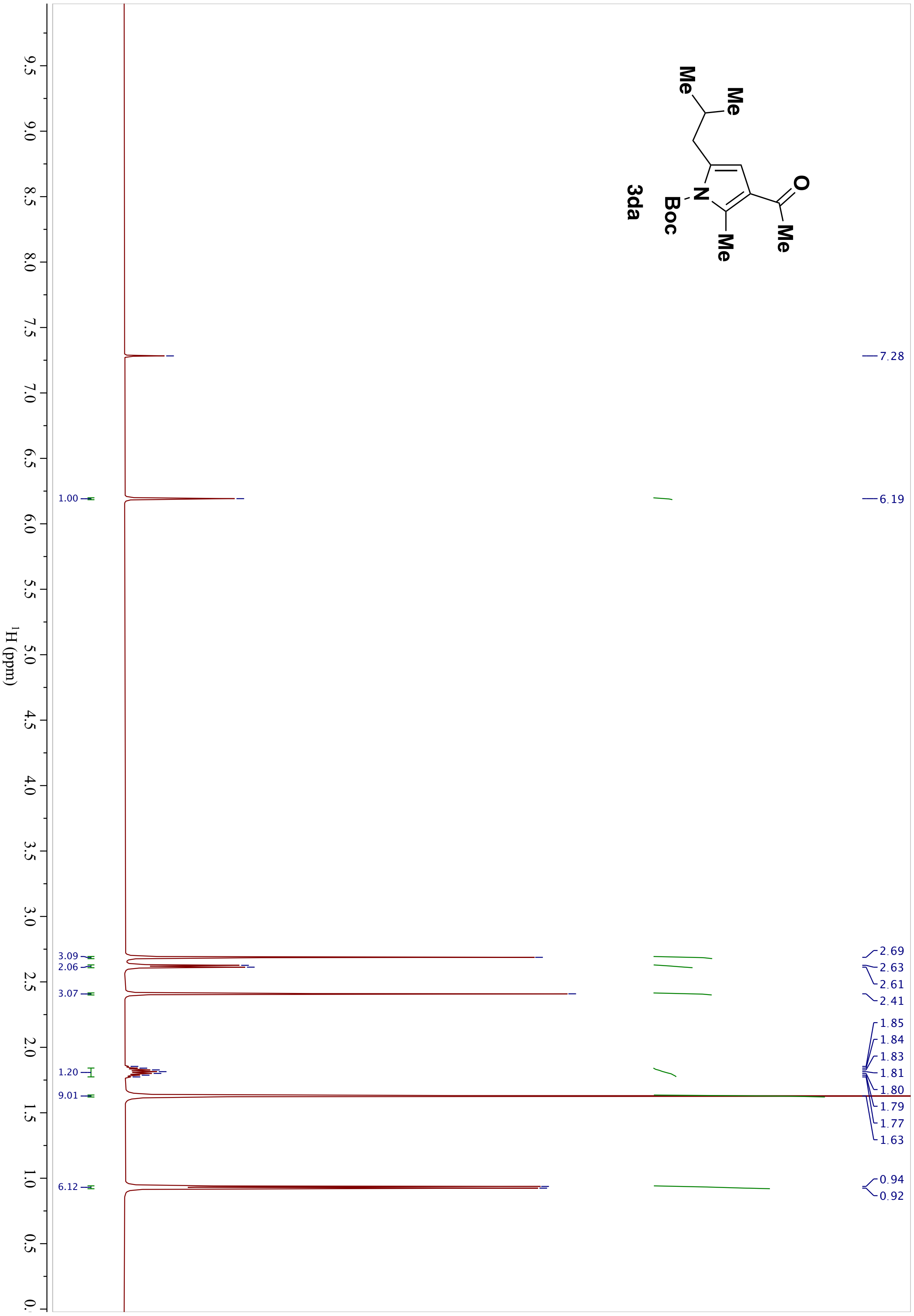

700 MHz, $CDCl_3$

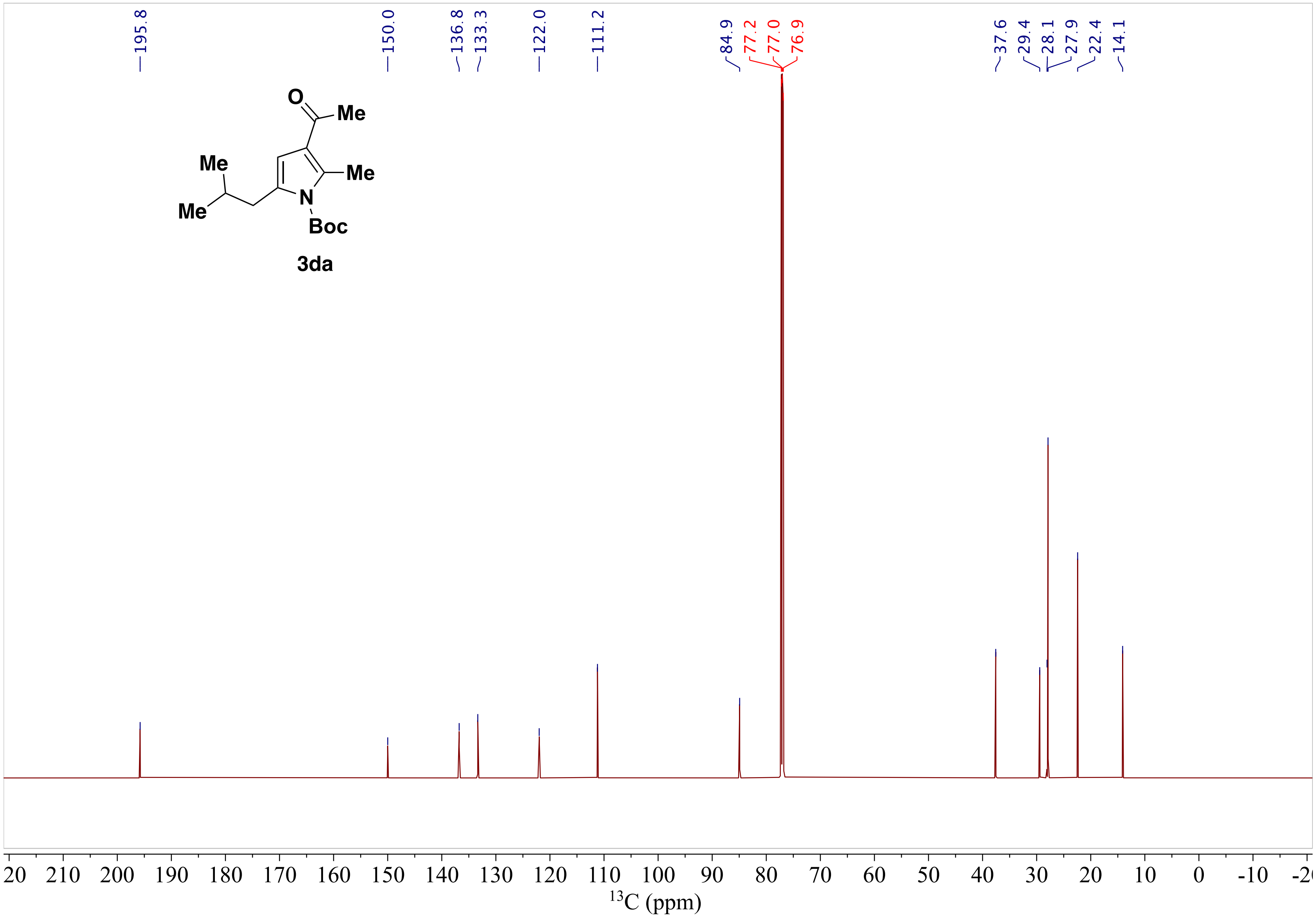

175 MHz, $CDCl_3$

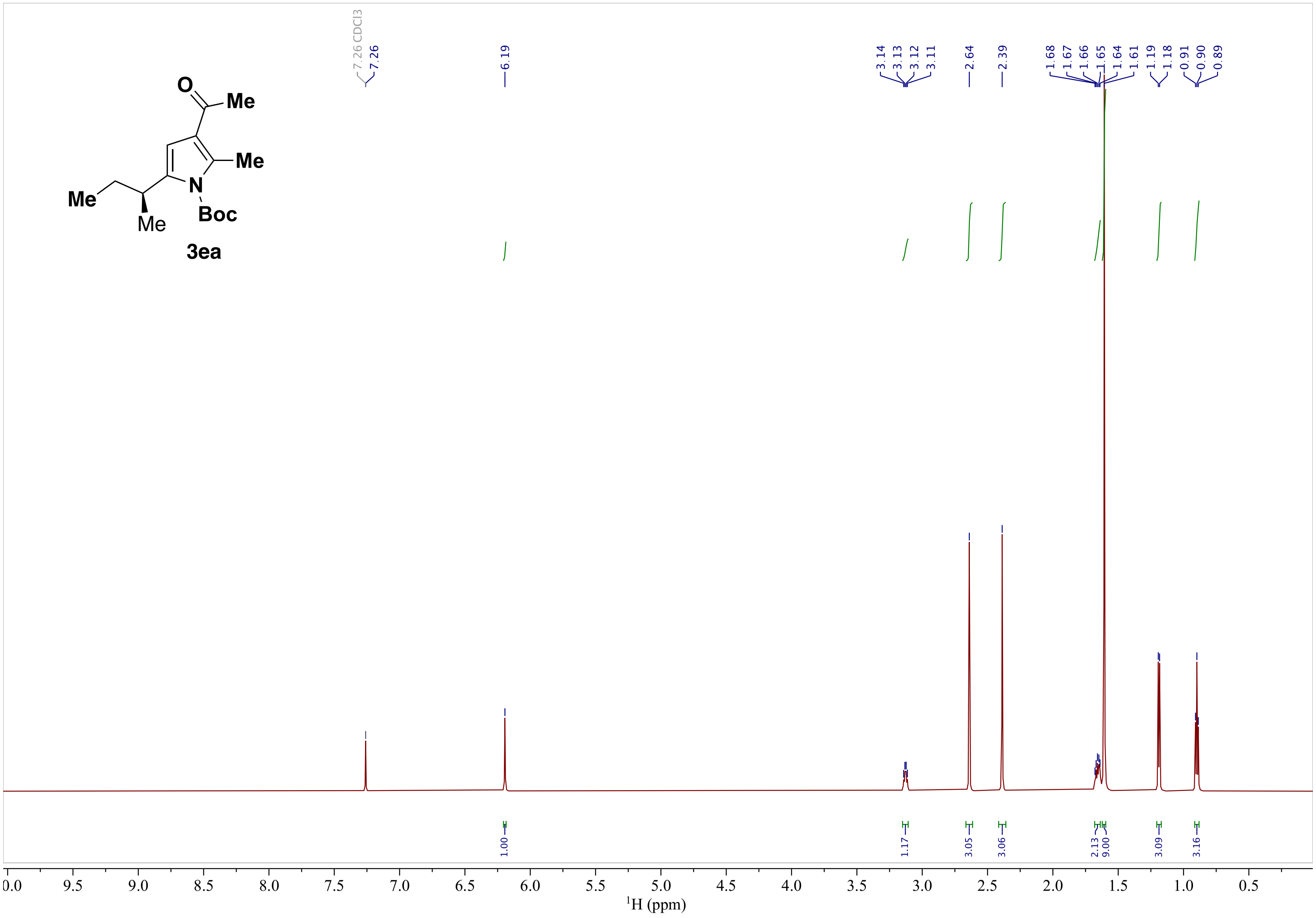

700 MHz, $CDCl_3$

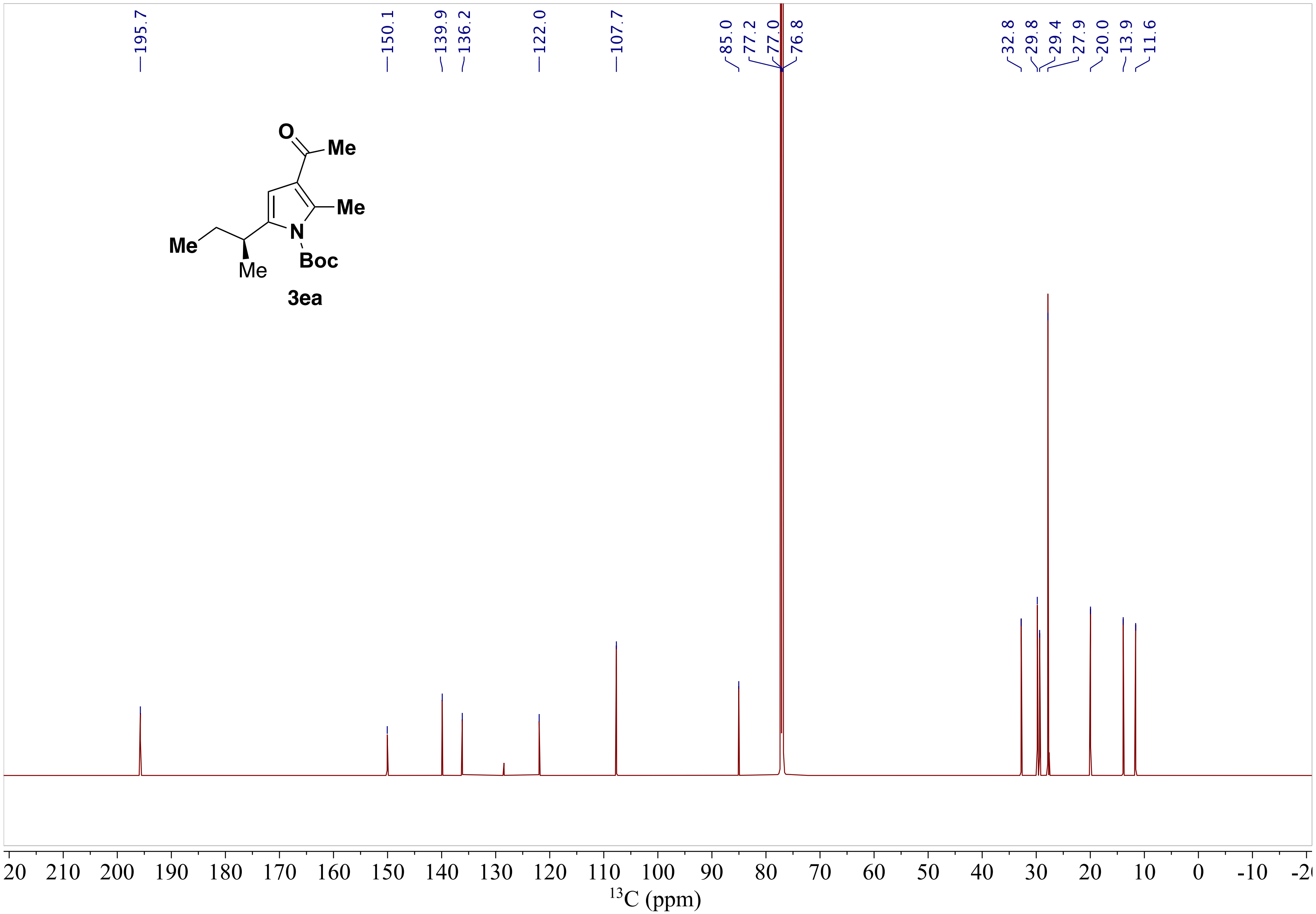

175 MHz, $CDCl_3$

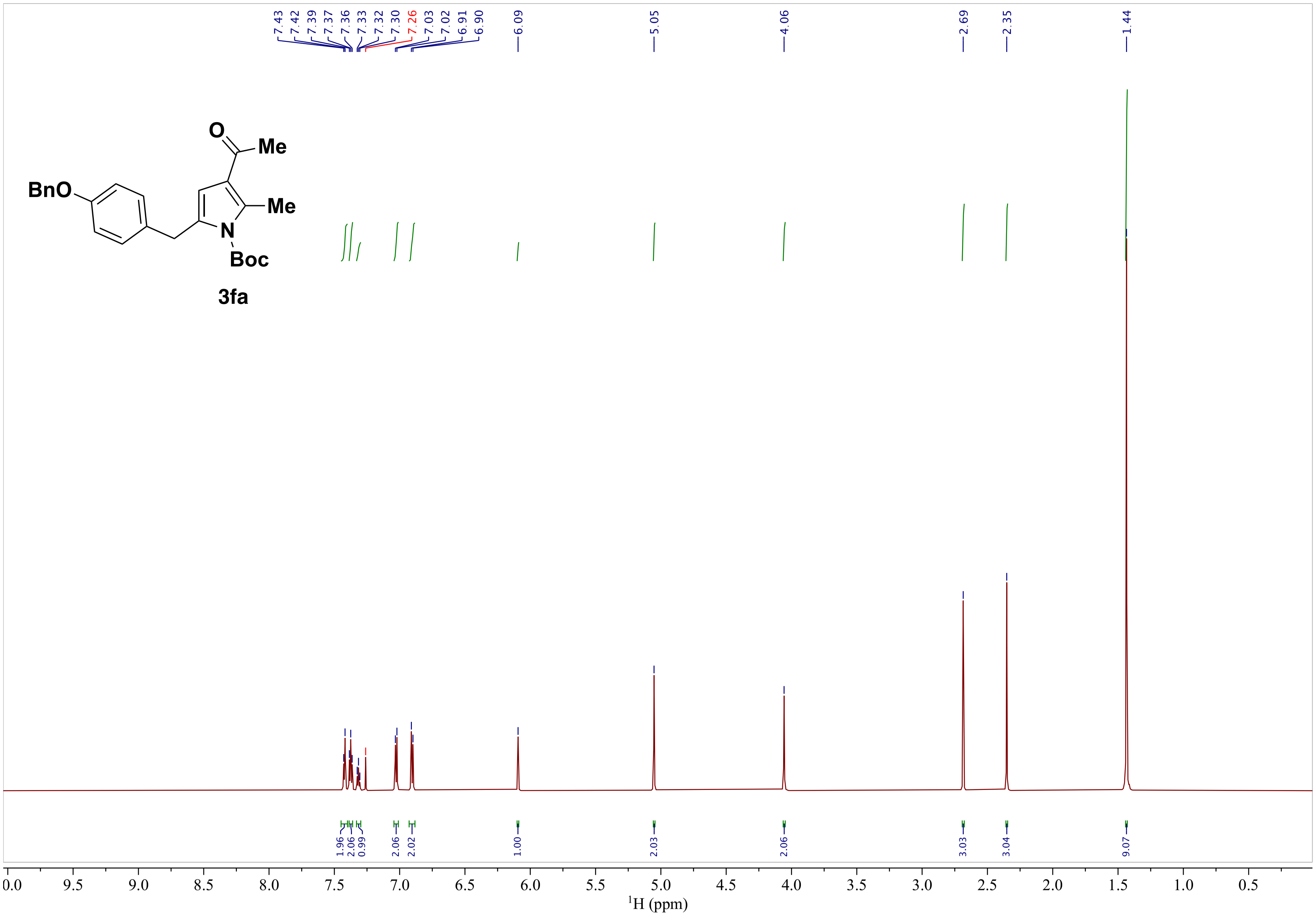

700 MHz, $CDCl_3$

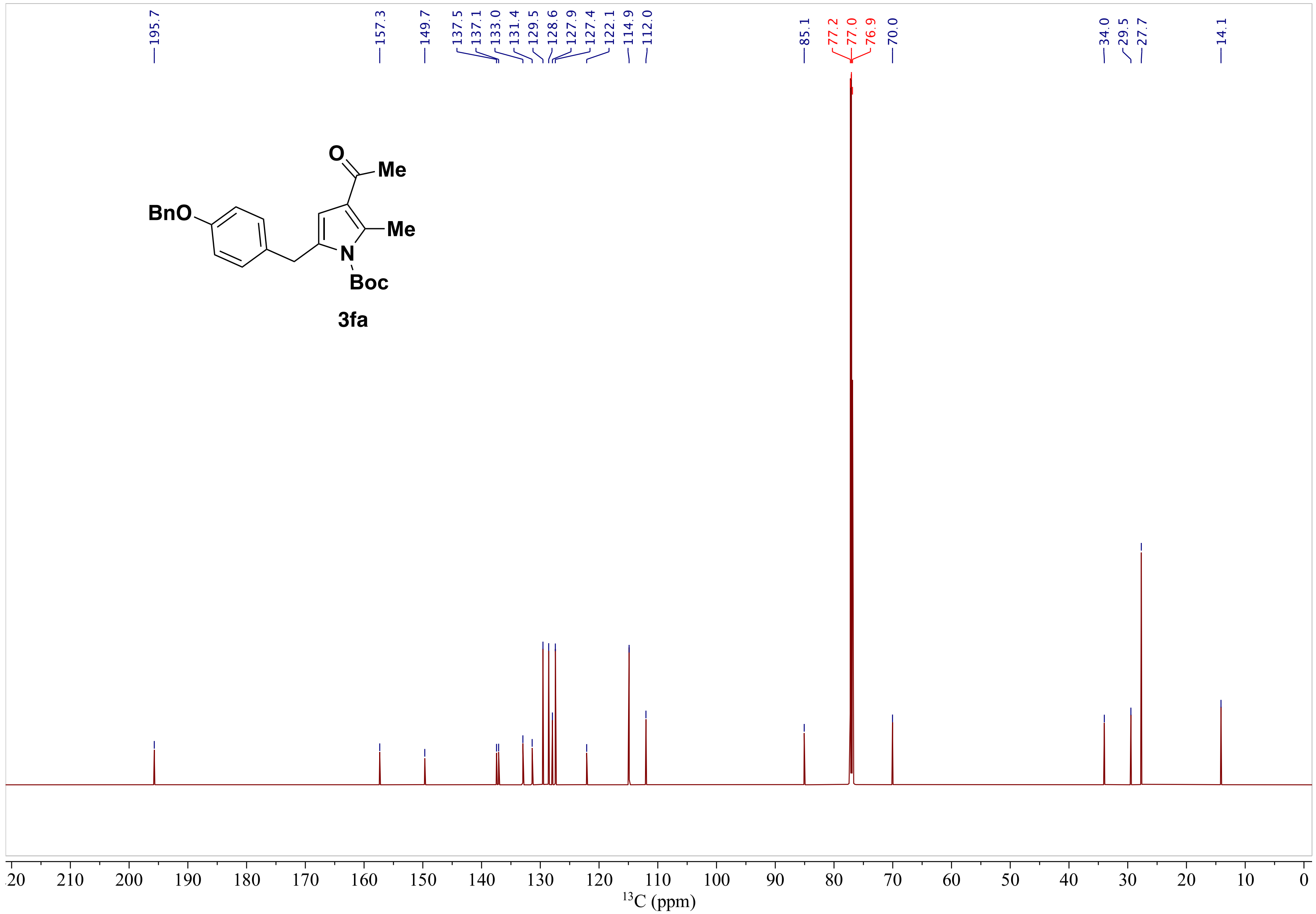

175 MHz, $CDCl_3$

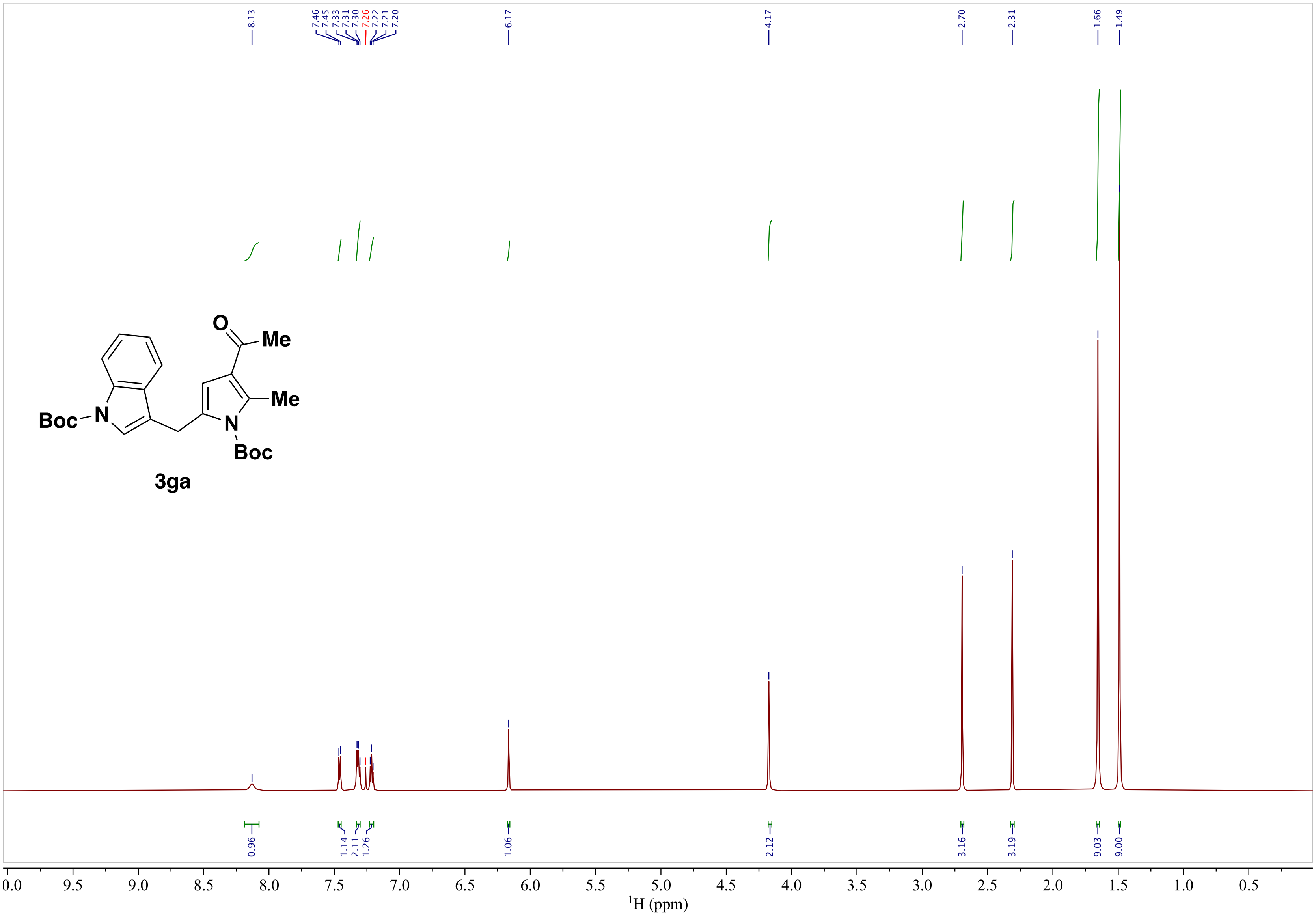

700 MHz, $CDCl_3$

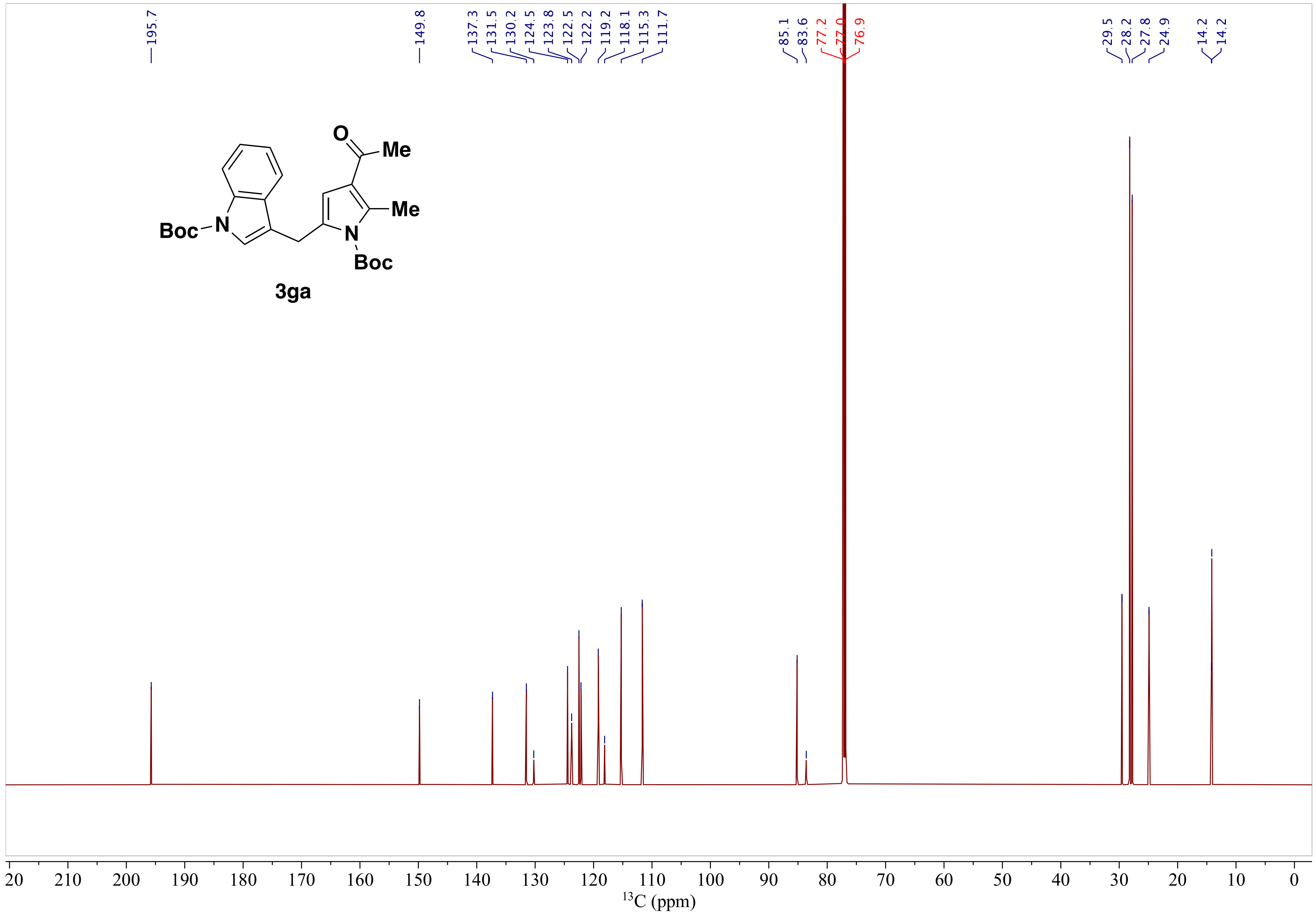

175 MHz, $CDCl_3$

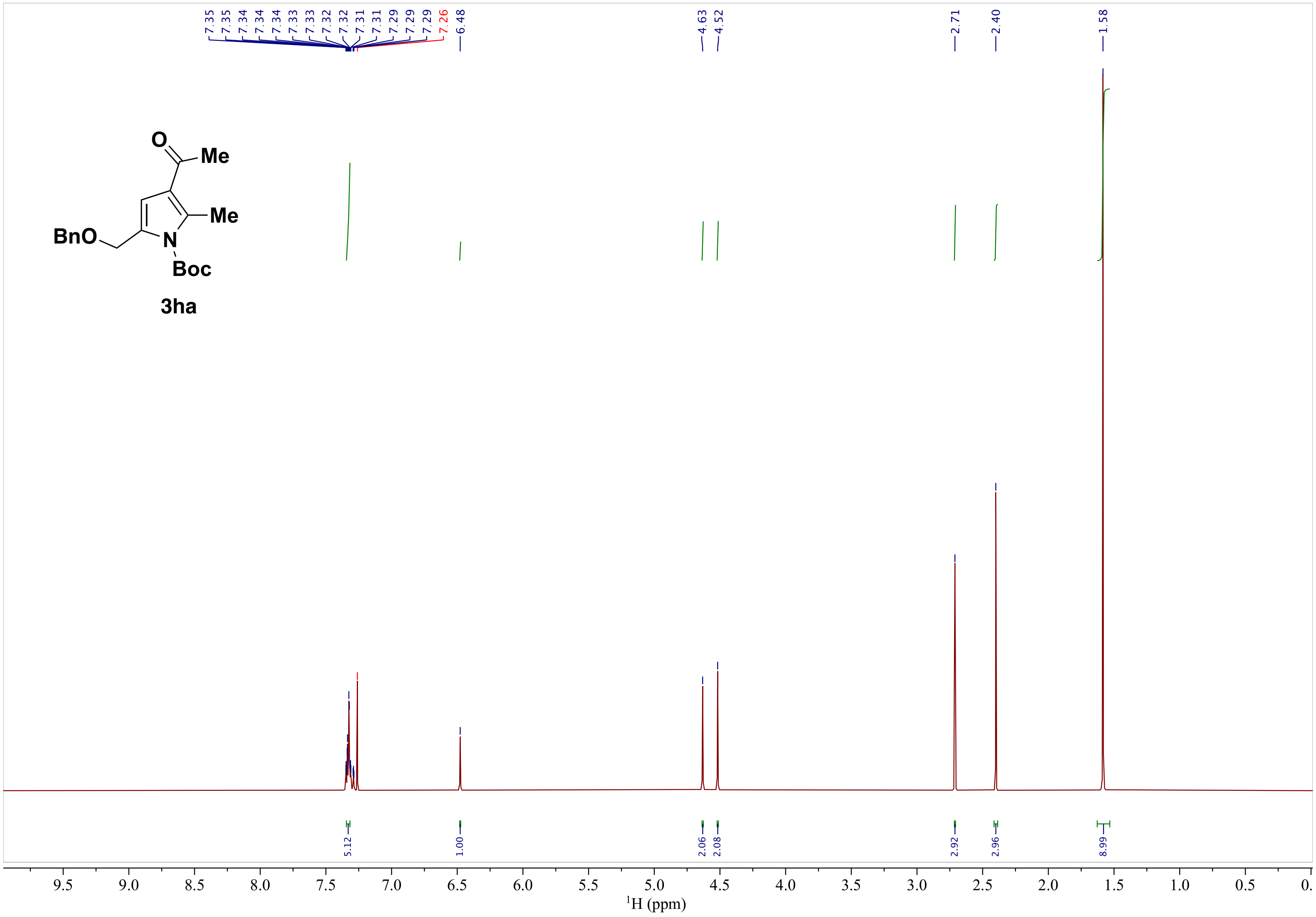

700 MHz, $CDCl_3$

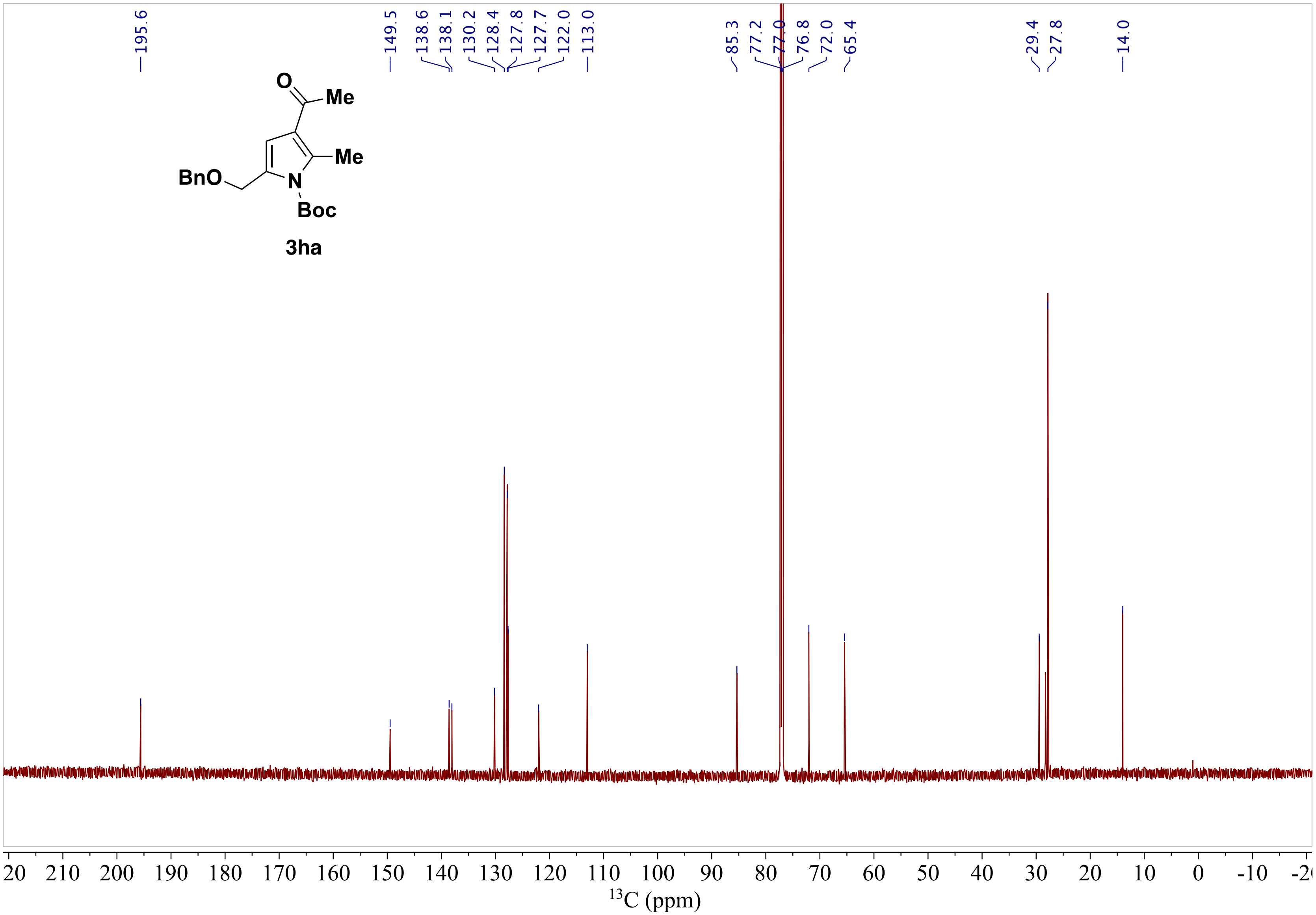

175 MHz, $CDCl_3$

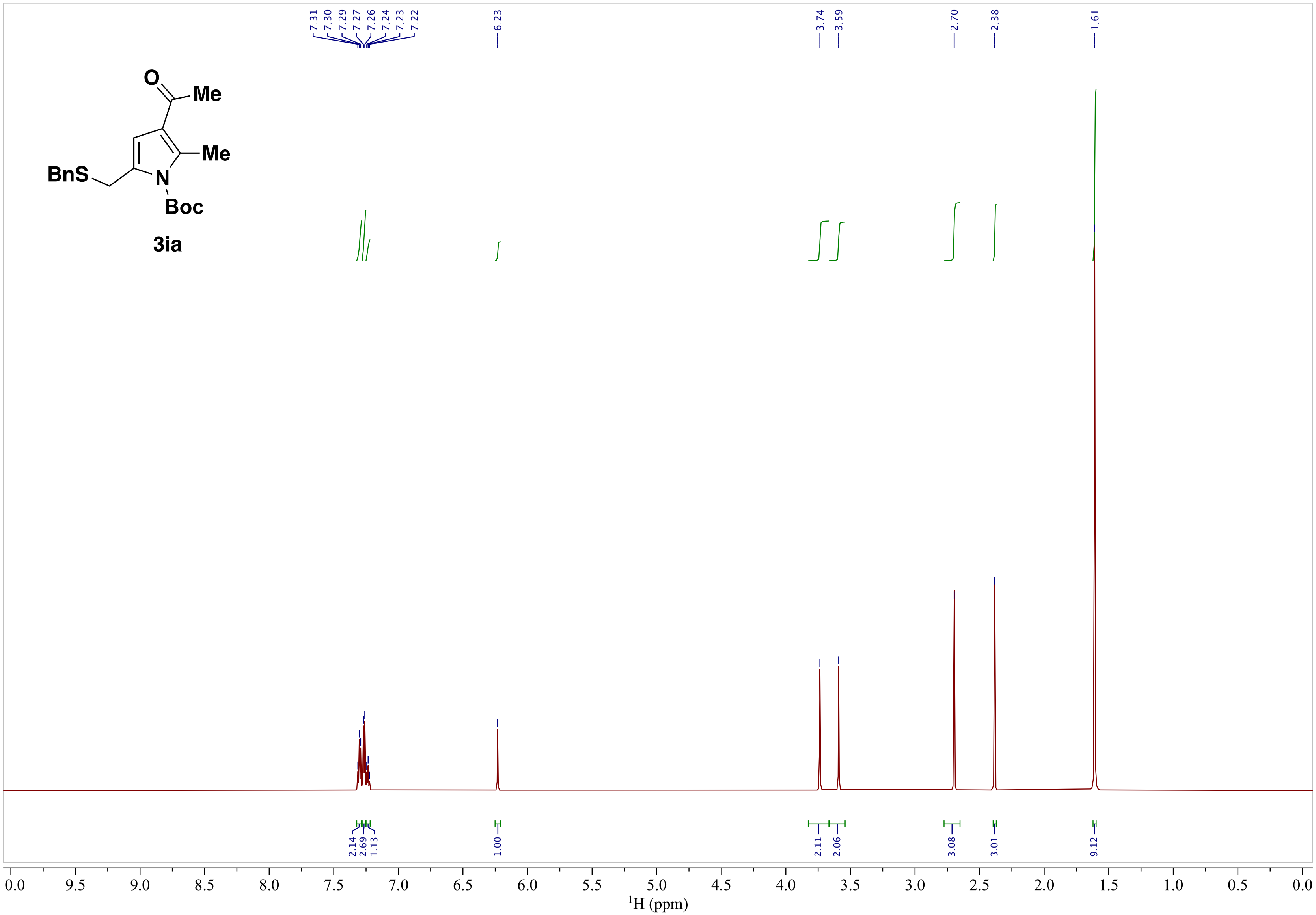

700 MHz, $CDCl_3$

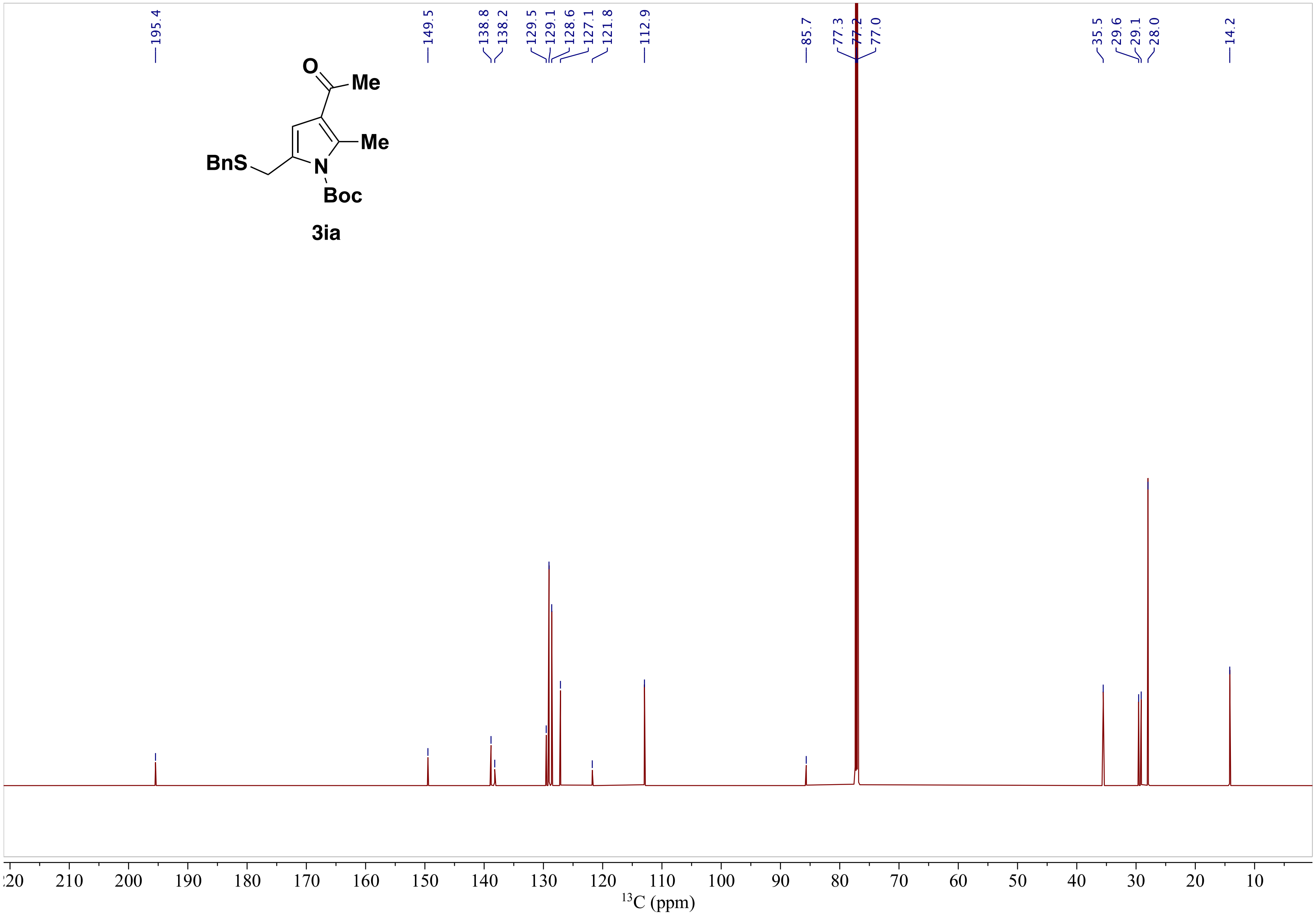

175 MHz, $CDCl_3$

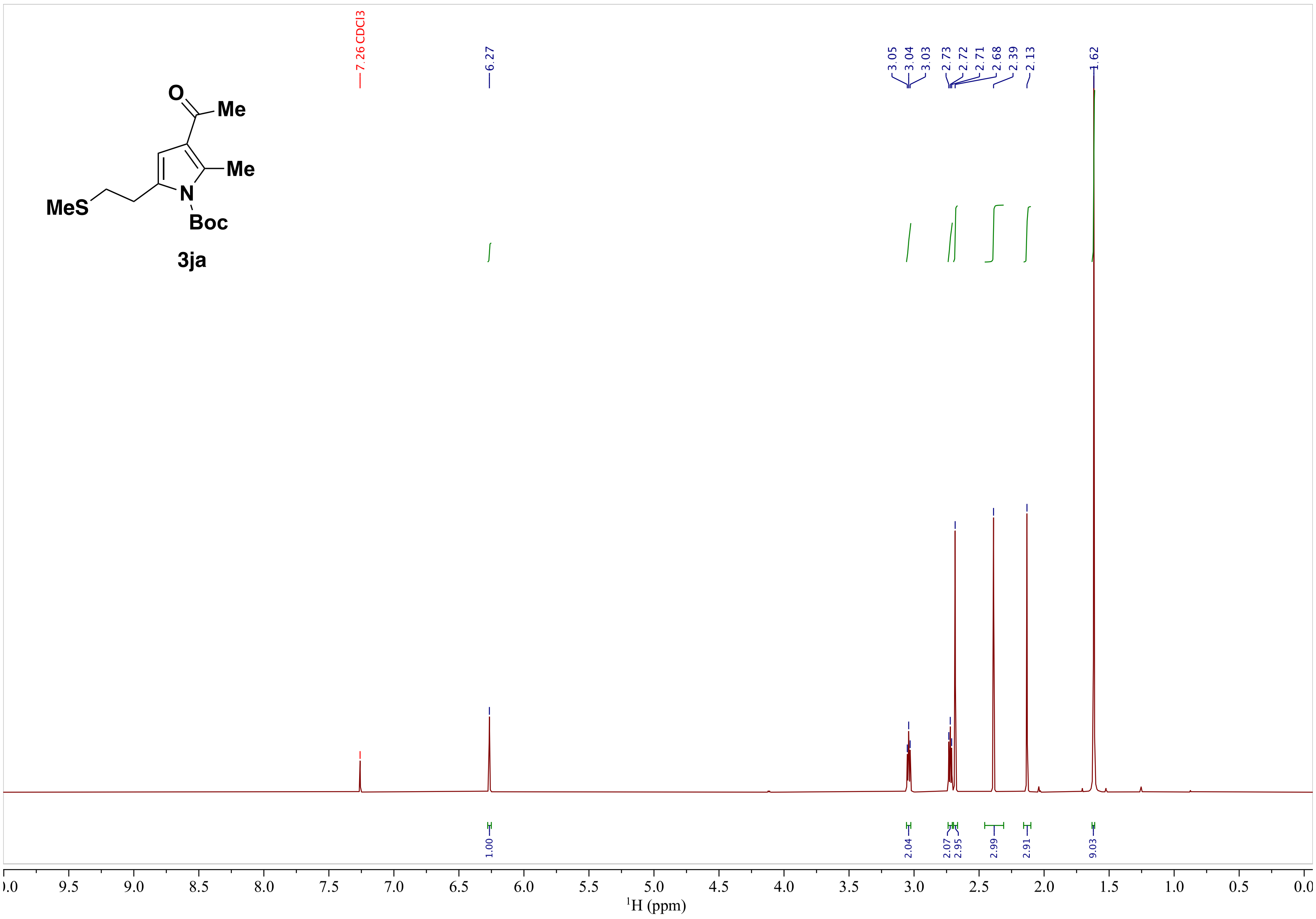

700 MHz, $CDCl_3$

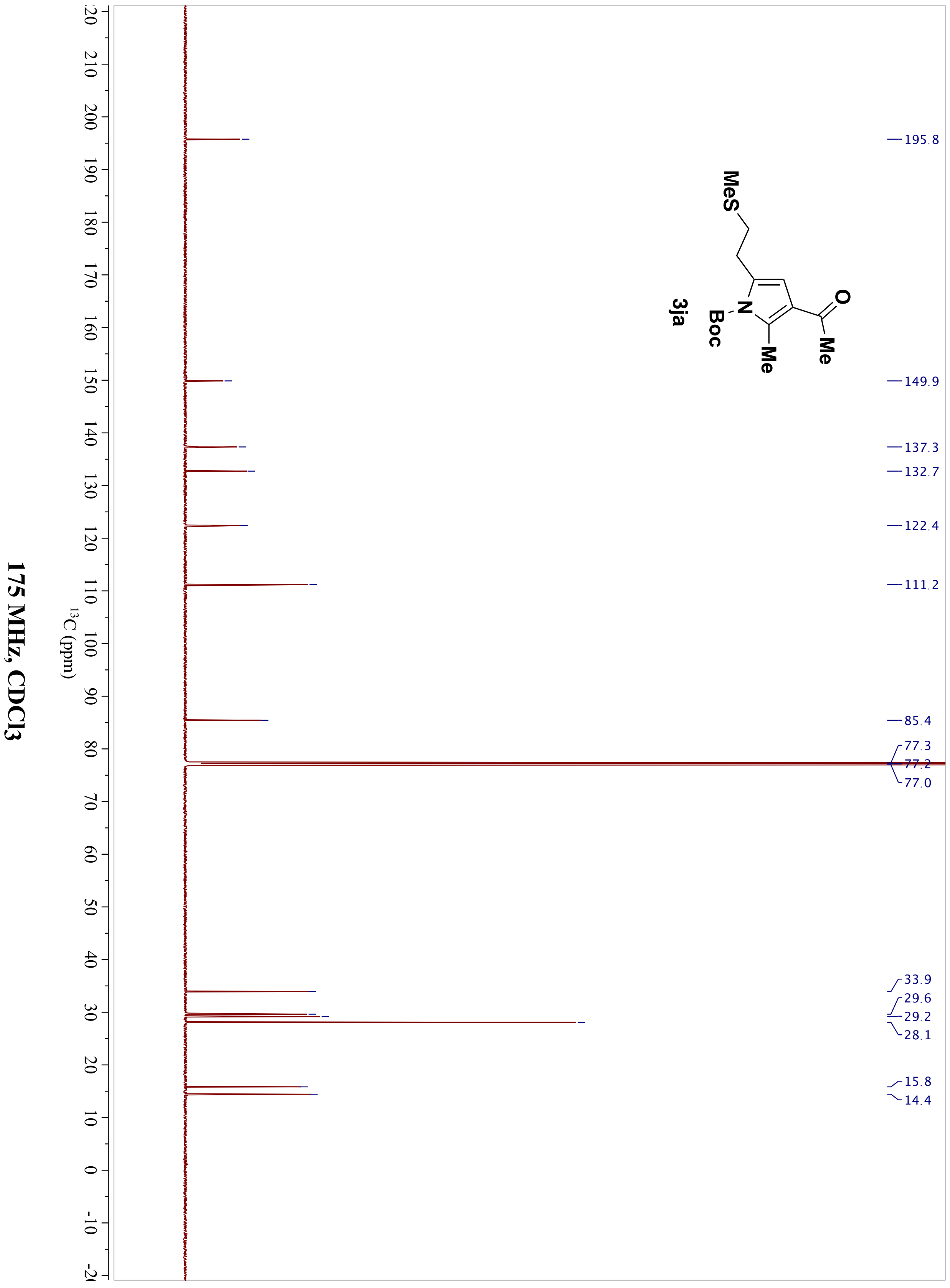

175 MHz, CDCl3
^{13}C (ppm)
3ja
MeS
O
Me
N
Boc
195.8
149.9
137.3
132.7
122.4
111.2
85.4
77.3
77.2
77.0
33.9
29.6
29.2
28.1
15.8
14.4

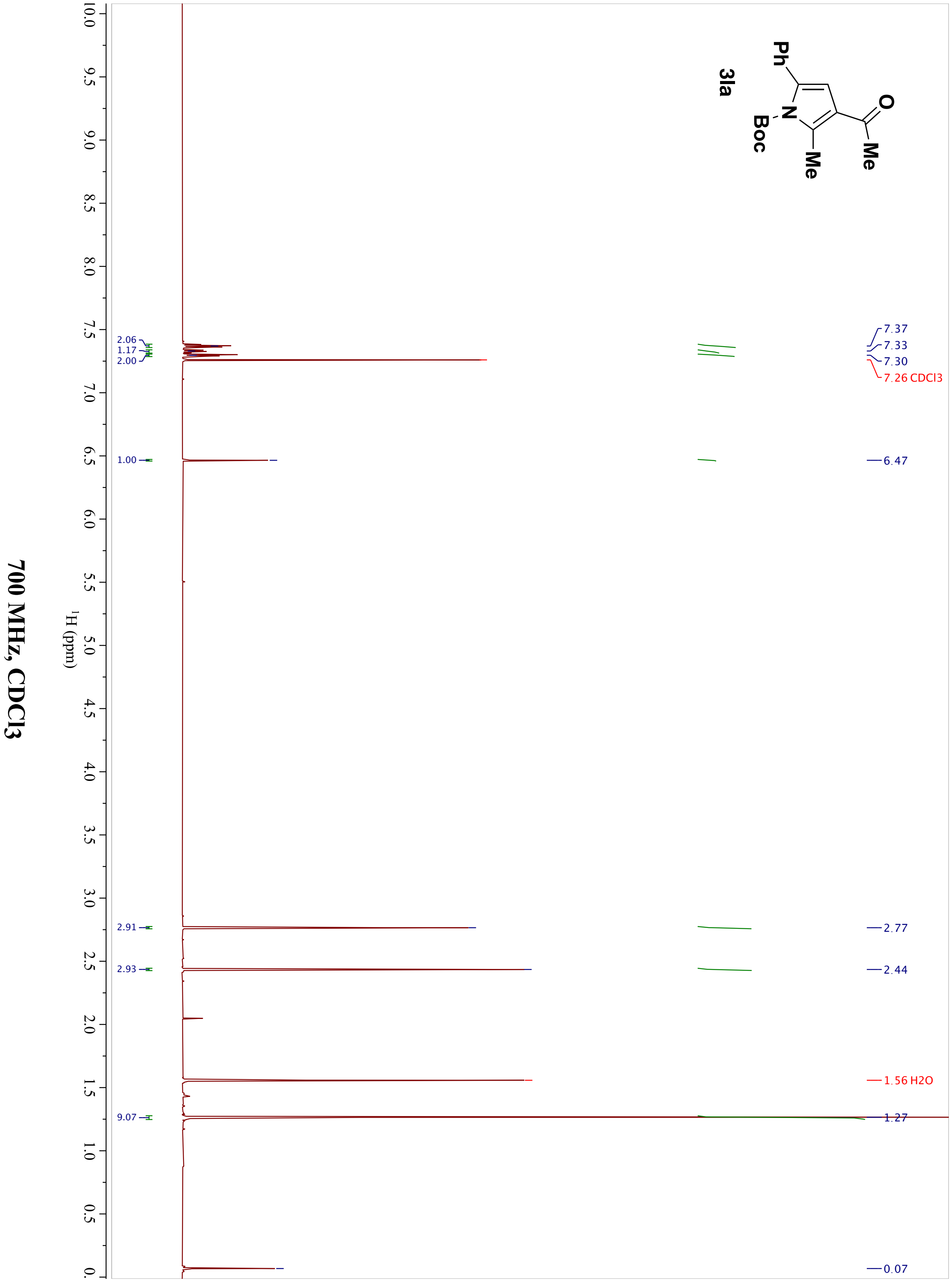

3la
Ph
N
Boc
Me
O
Me
700 MHz, CDCl3
1H (ppm)
7.37
7.33
7.30
7.26 CDCl3
6.47
2.77
2.44
1.56 H2O
1.27
0.07
2.06
1.17
2.00
1.00
2.91
2.93
9.07

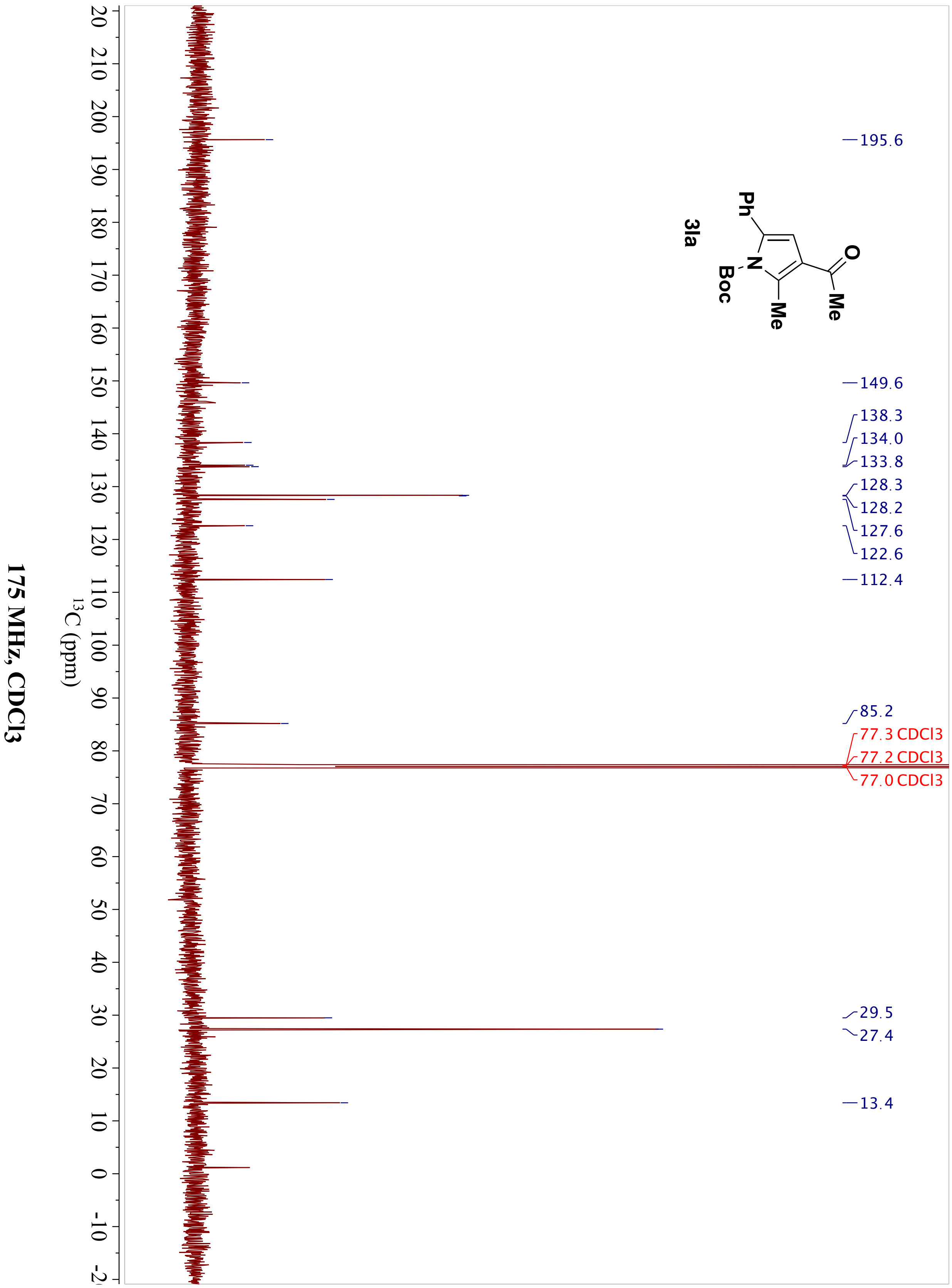
175 MHz, CDCl3
13C (ppm)
3la
Ph
N
Boc
Me
O
Me
195.6
149.6
138.3
134.0
133.8
128.3
128.2
127.6
122.6
112.4
85.2
77.3 CDCl3
77.2 CDCl3
77.0 CDCl3
29.5
27.4
13.4

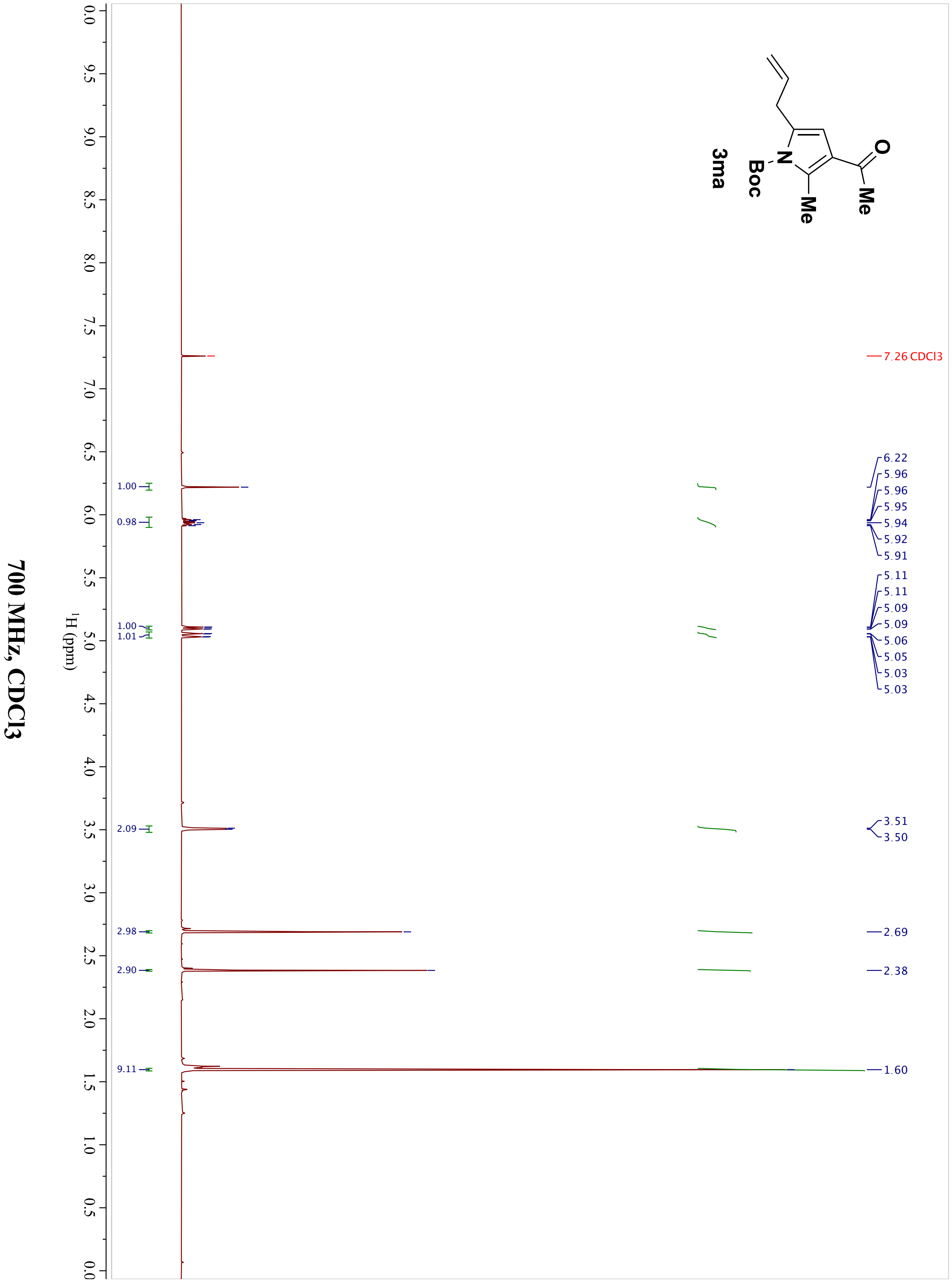
O
Me
Me
N
Boc
3ma
7.26 CDCl3
6.22
5.96
5.96
5.95
5.94
5.92
5.91
5.11
5.11
5.09
5.09
5.06
5.05
5.03
5.03
3.51
3.50
2.69
2.38
1.60
1.00
0.98
1.00
1.01
2.09
2.98
2.90
9.11
^{1}H (ppm)
700 MHz, CDCl3

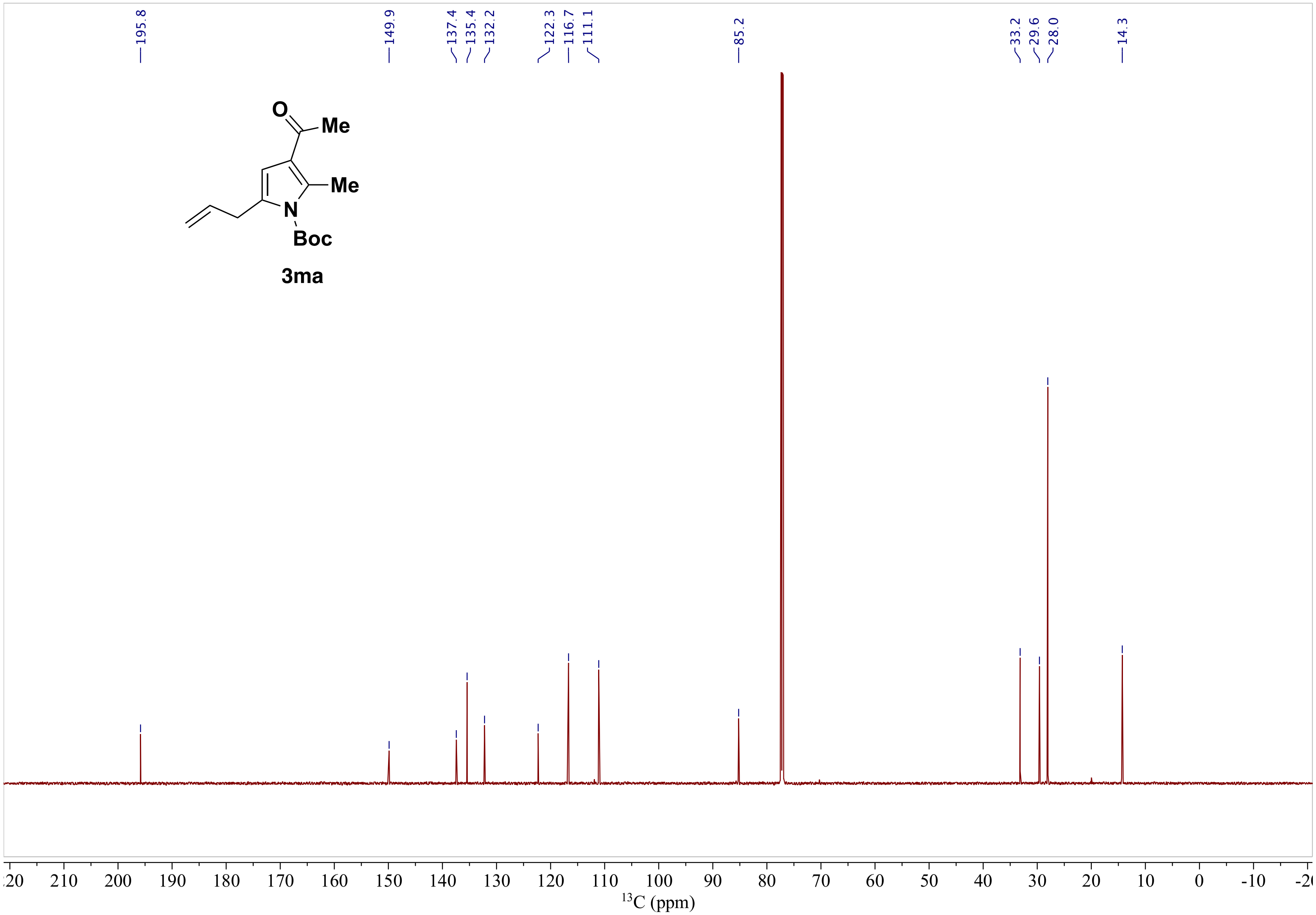

175 MHz, $CDCl_3$

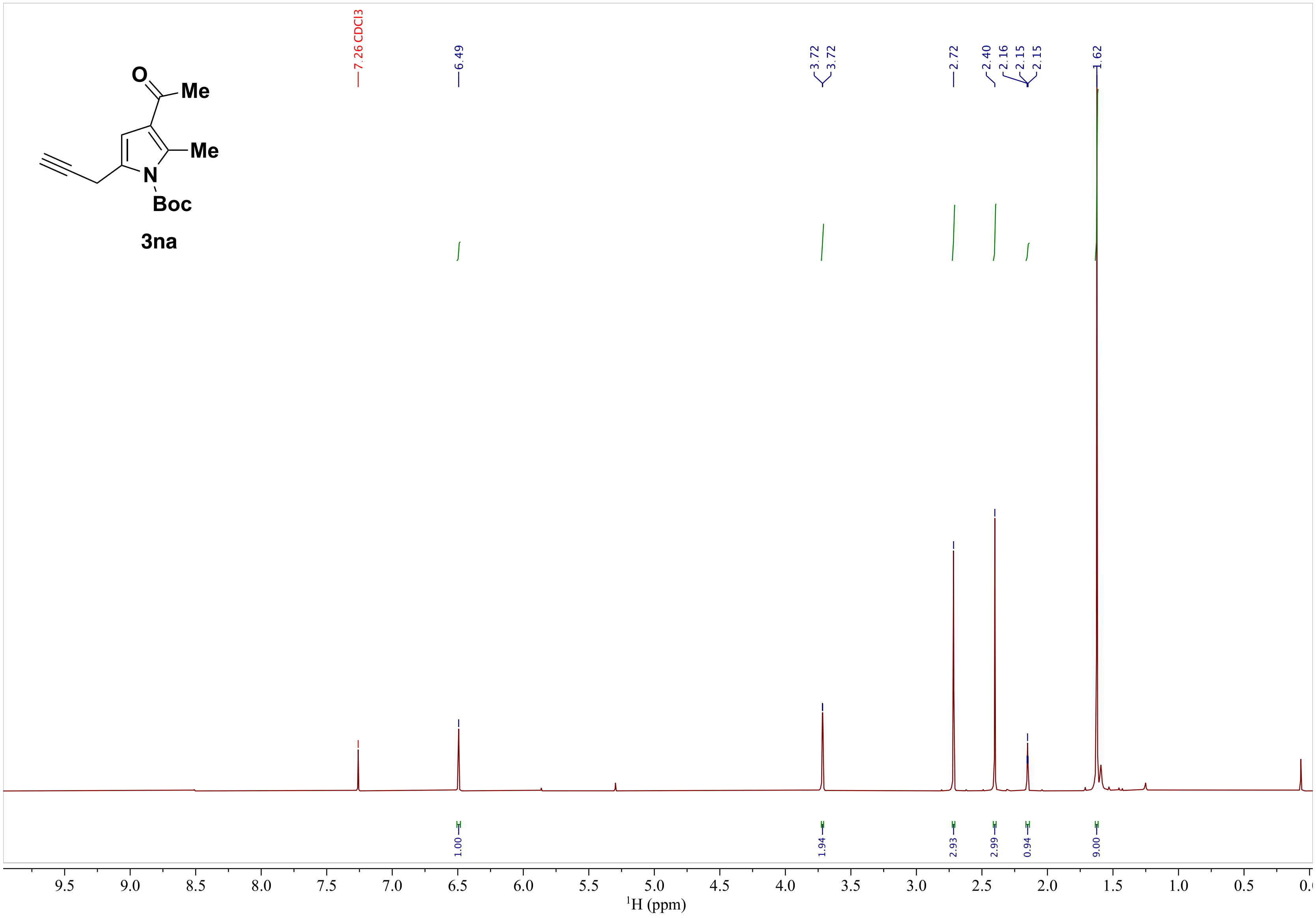

700 MHz, $CDCl_3$

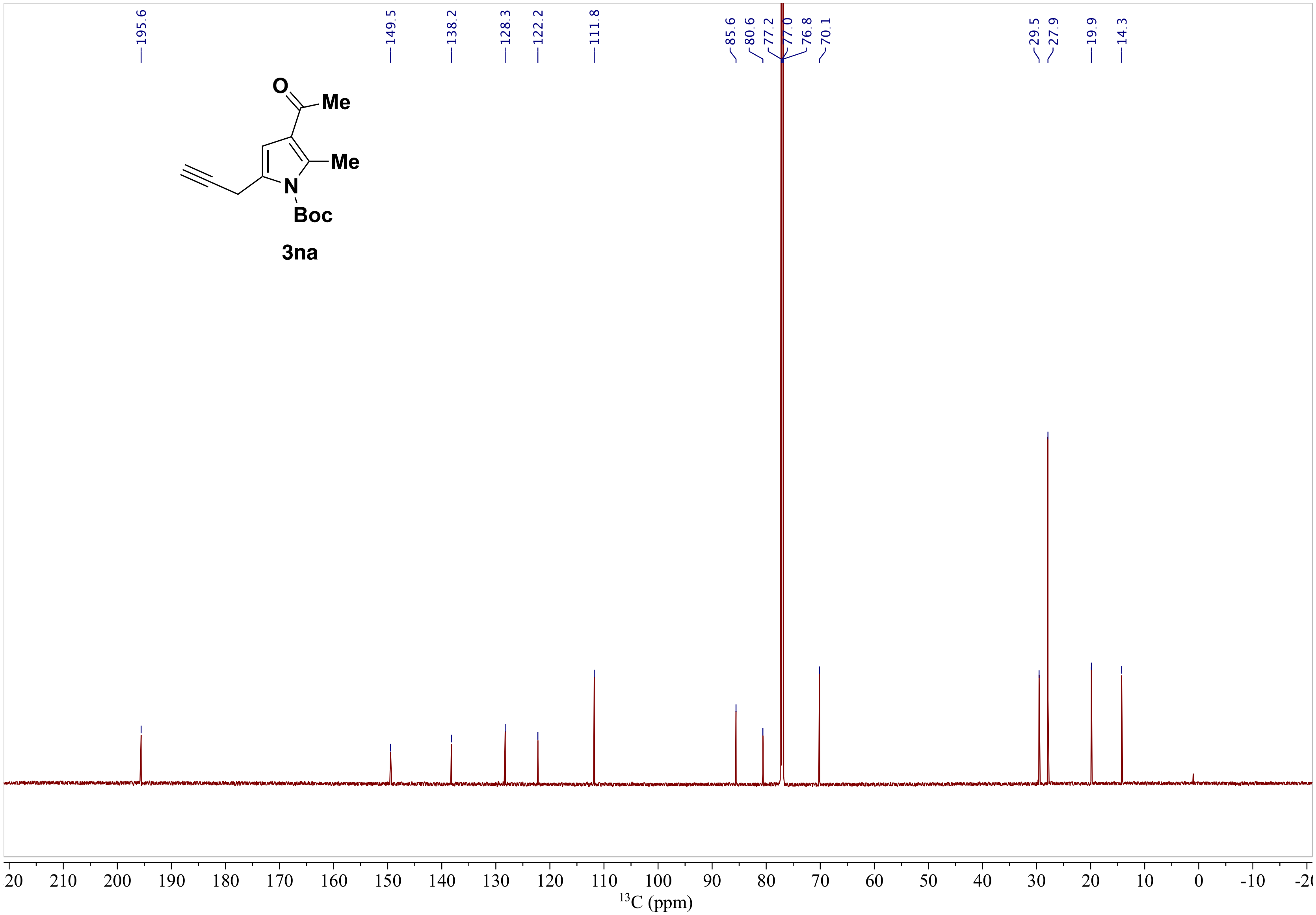

175 MHz, $CDCl_3$

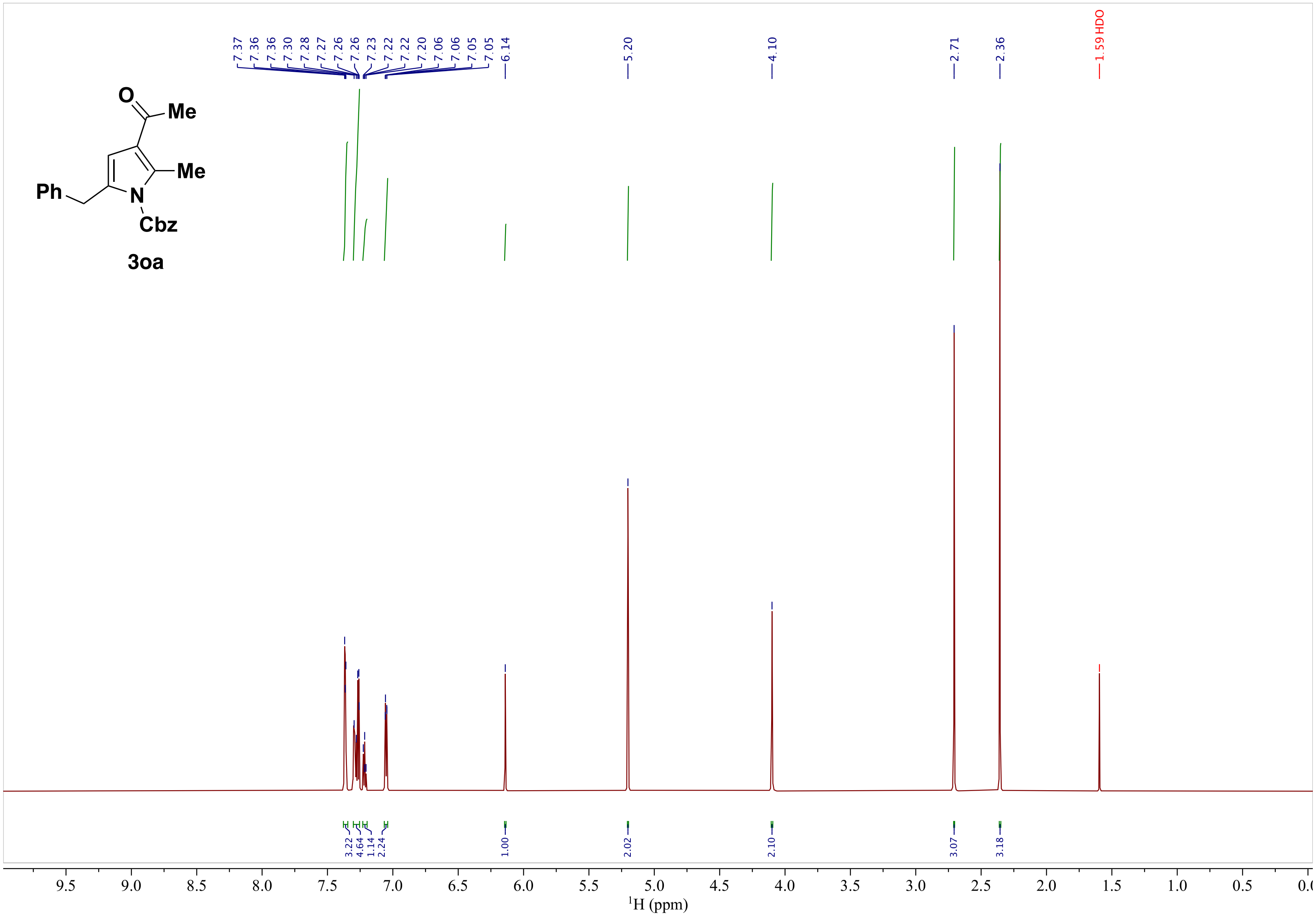

700 MHz, $CDCl_3$

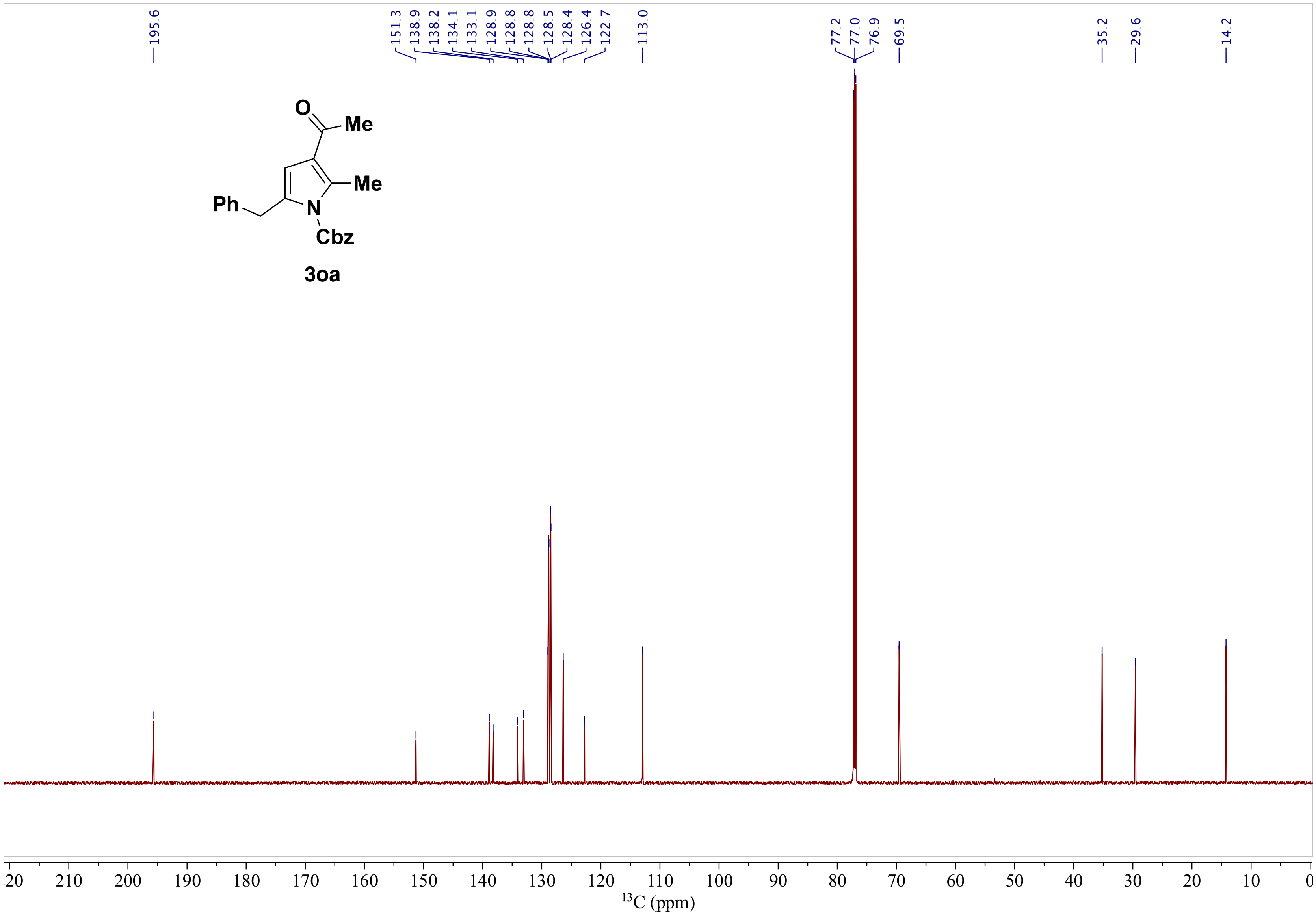

175 MHz, $CDCl_3$

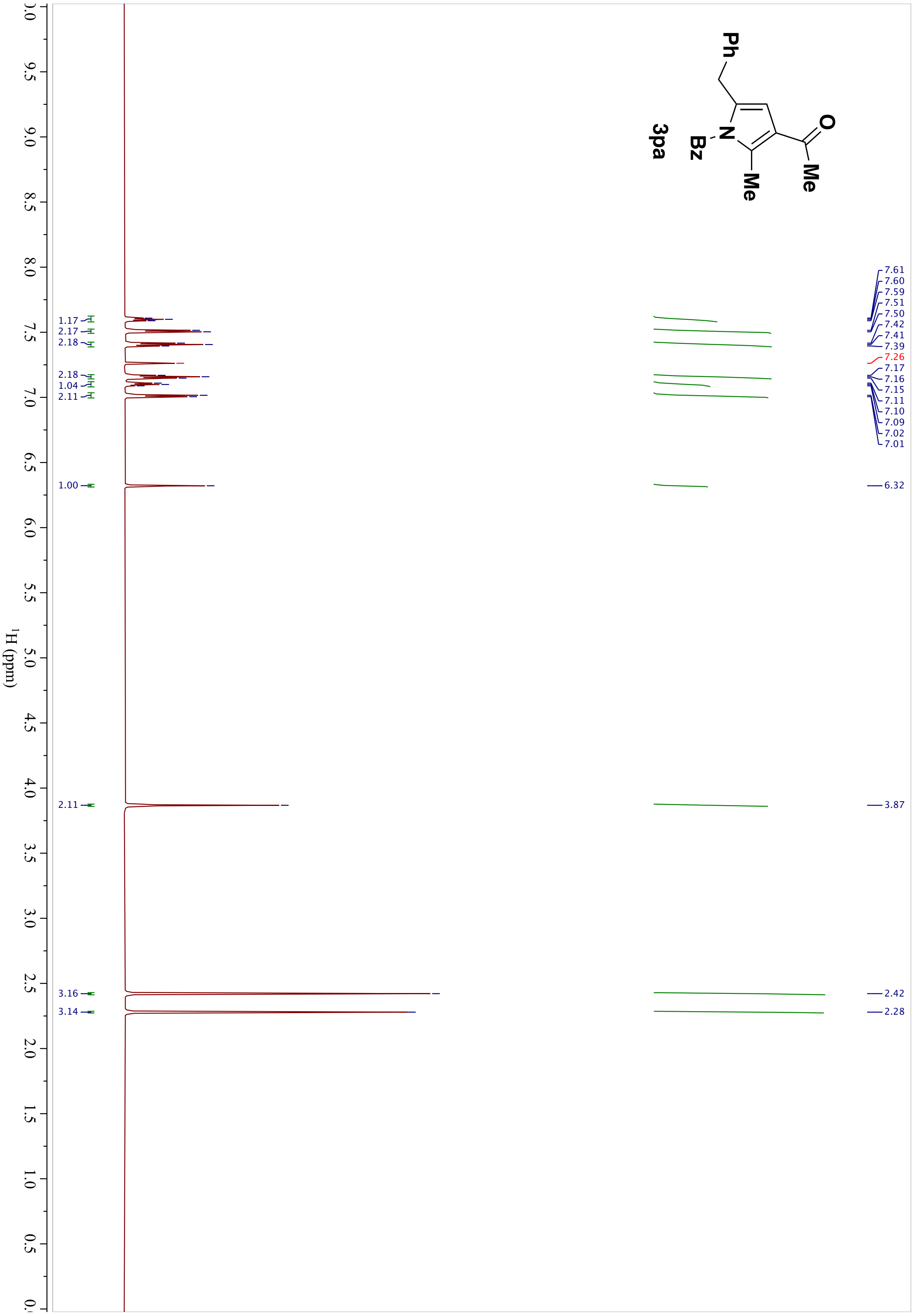

700 MHz, CDCl3

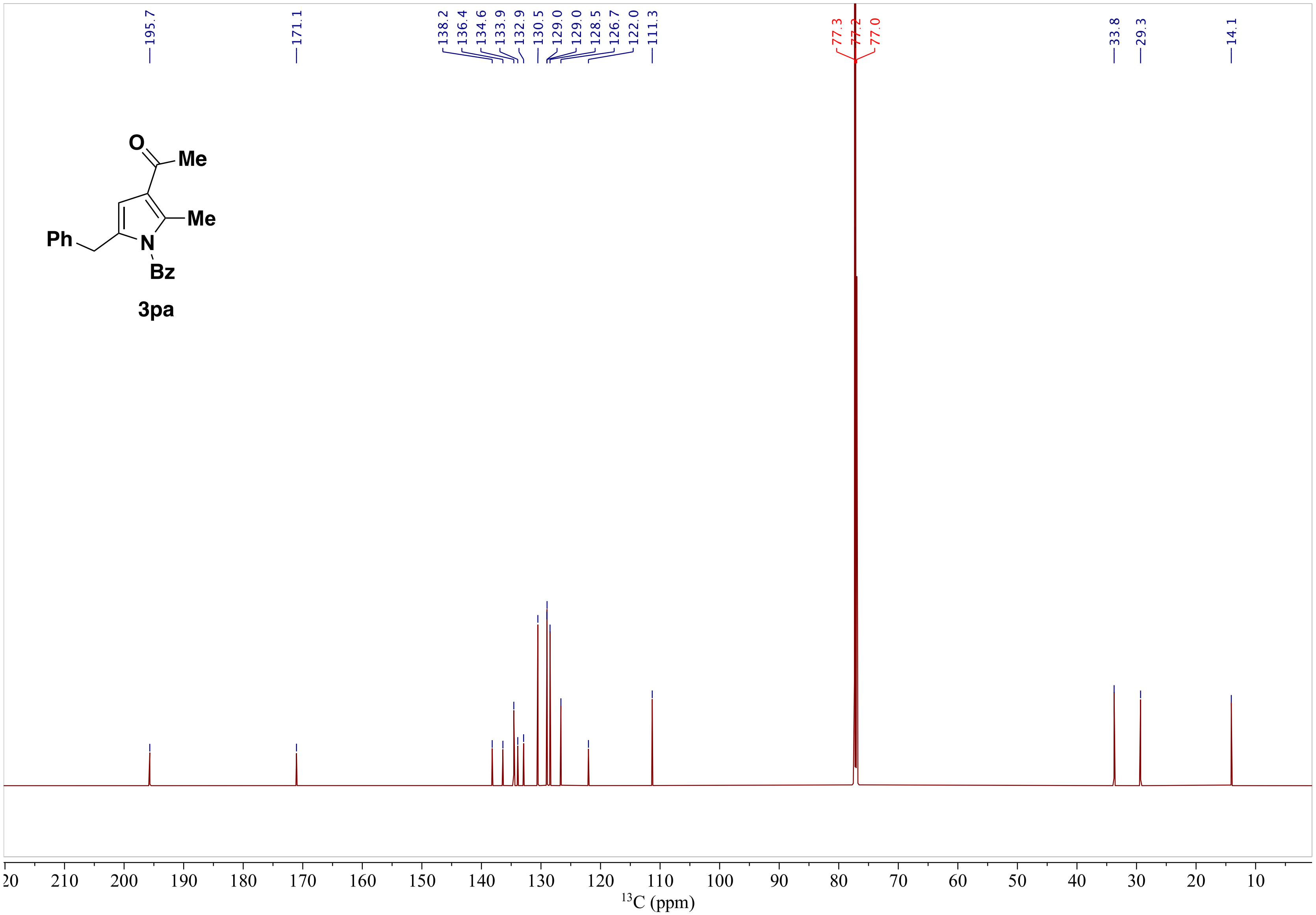

175 MHz, $CDCl_3$

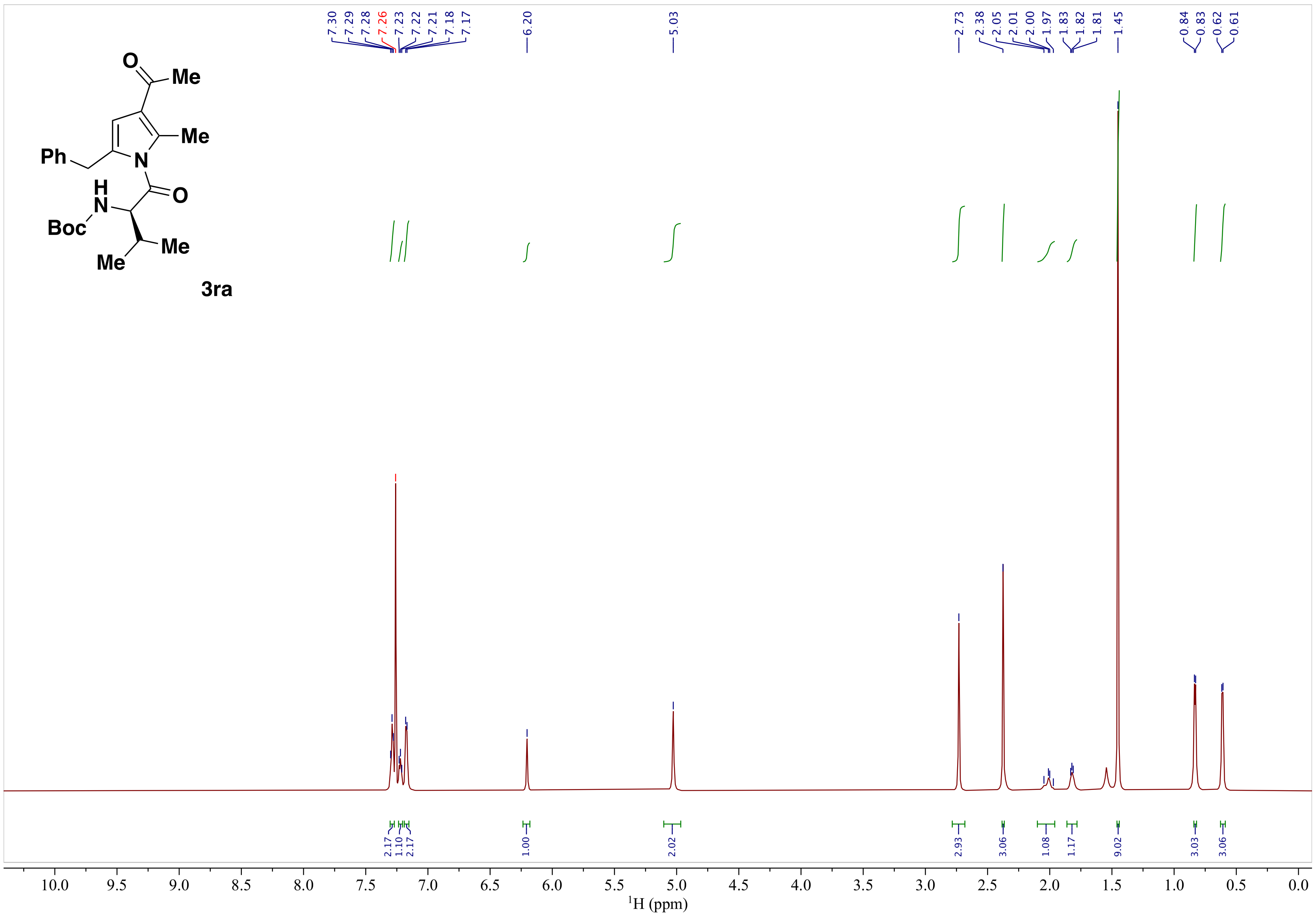

700 MHz, $CDCl_3$

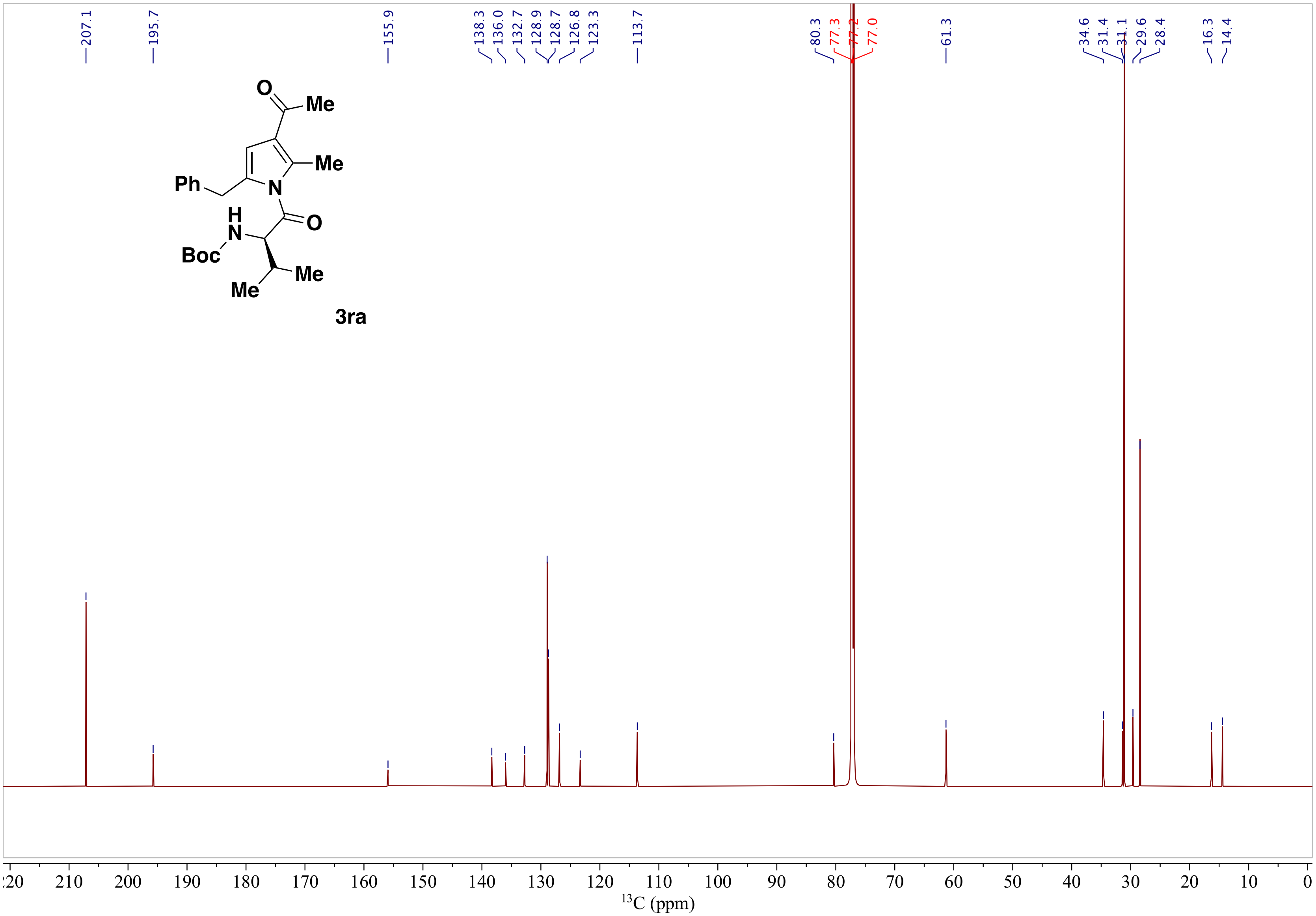

175 MHz, $CDCl_3$

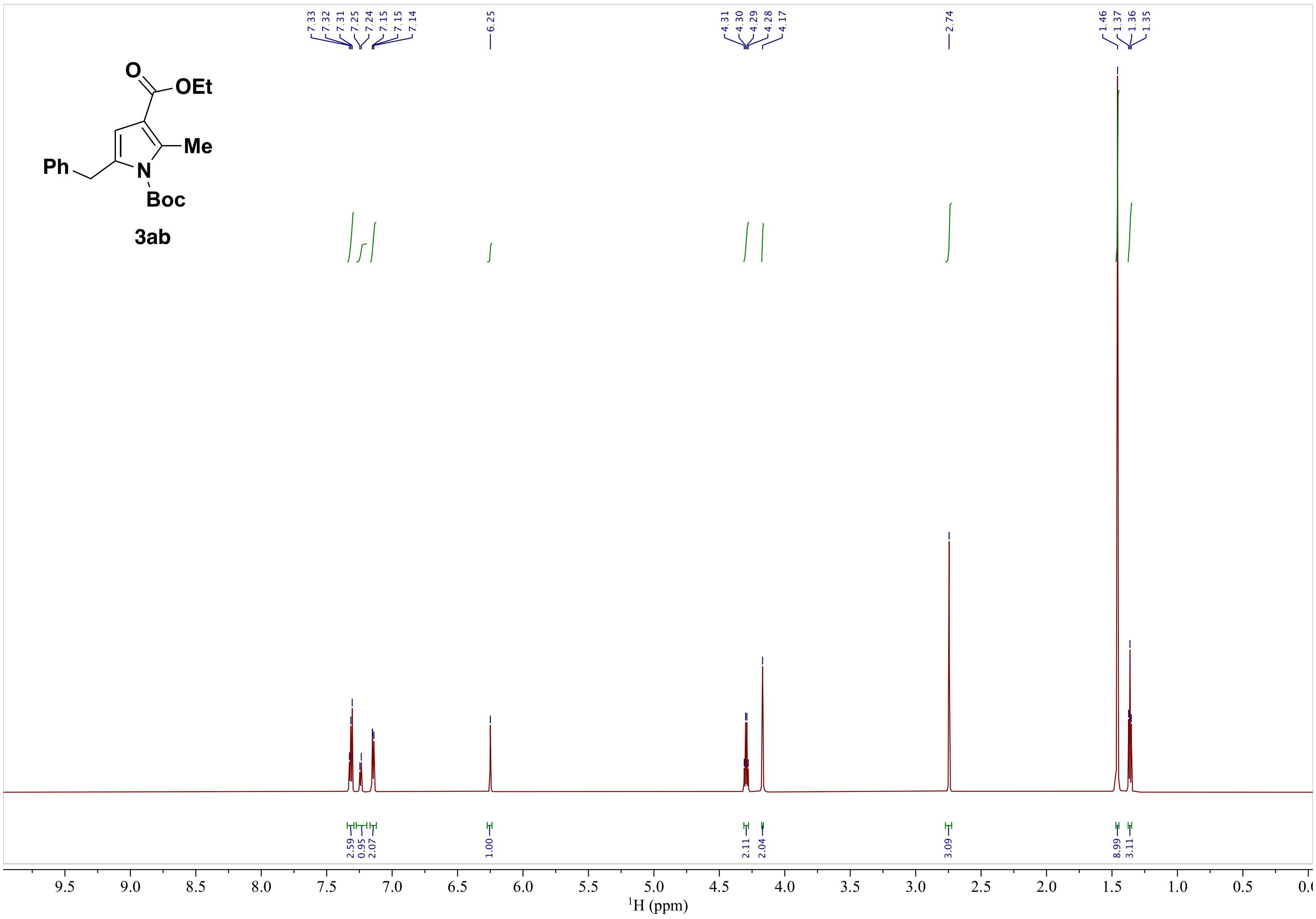

700 MHz, $CDCl_3$

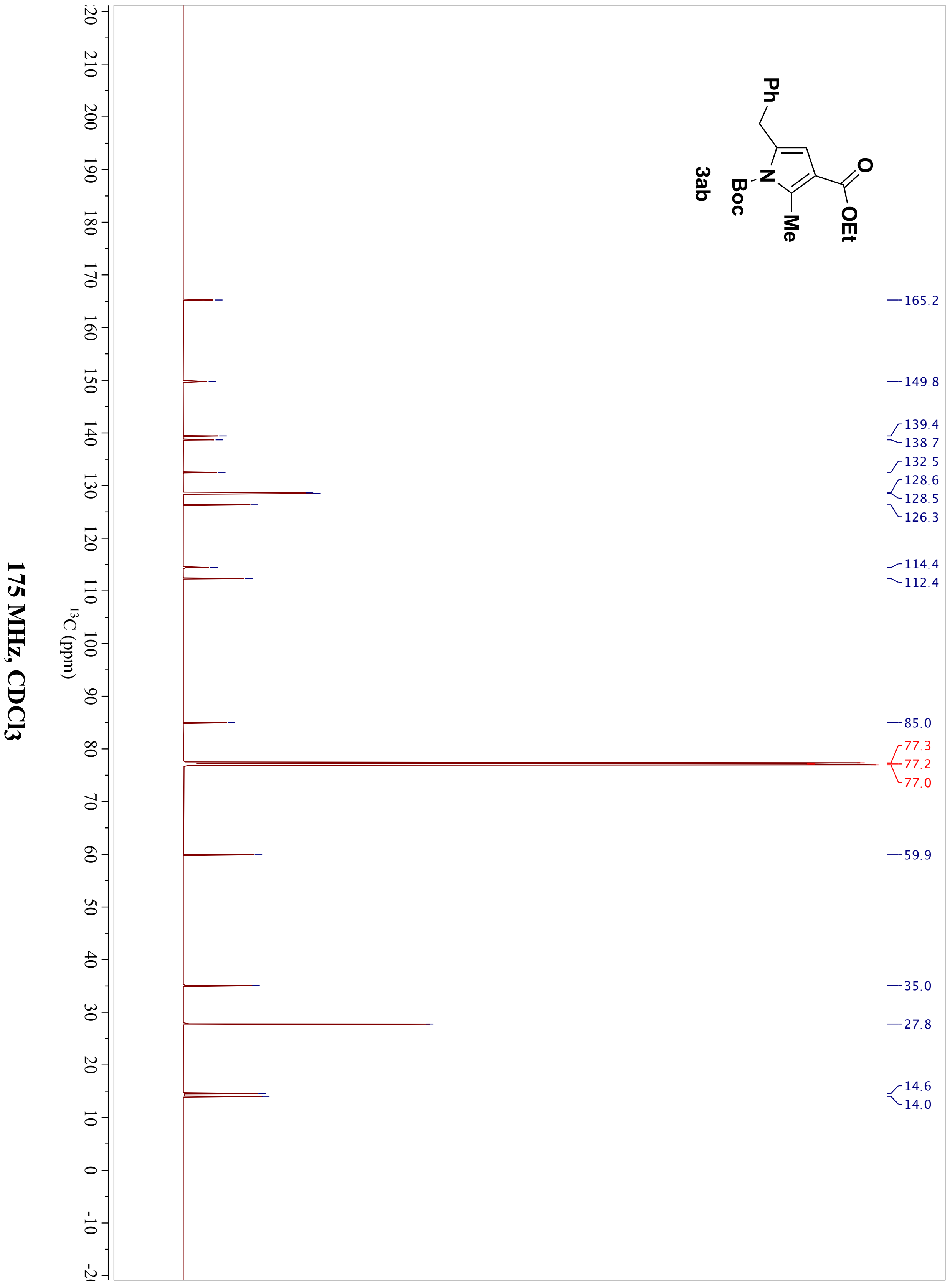
175 MHz, CDCl3
3ab
Ph
O
OEt
Me
N
Boc
165.2
149.8
139.4
138.7
132.5
128.6
128.5
126.3
114.4
112.4
85.0
77.3
77.2
77.0
59.9
35.0
27.8
14.6
14.0
13C (ppm)

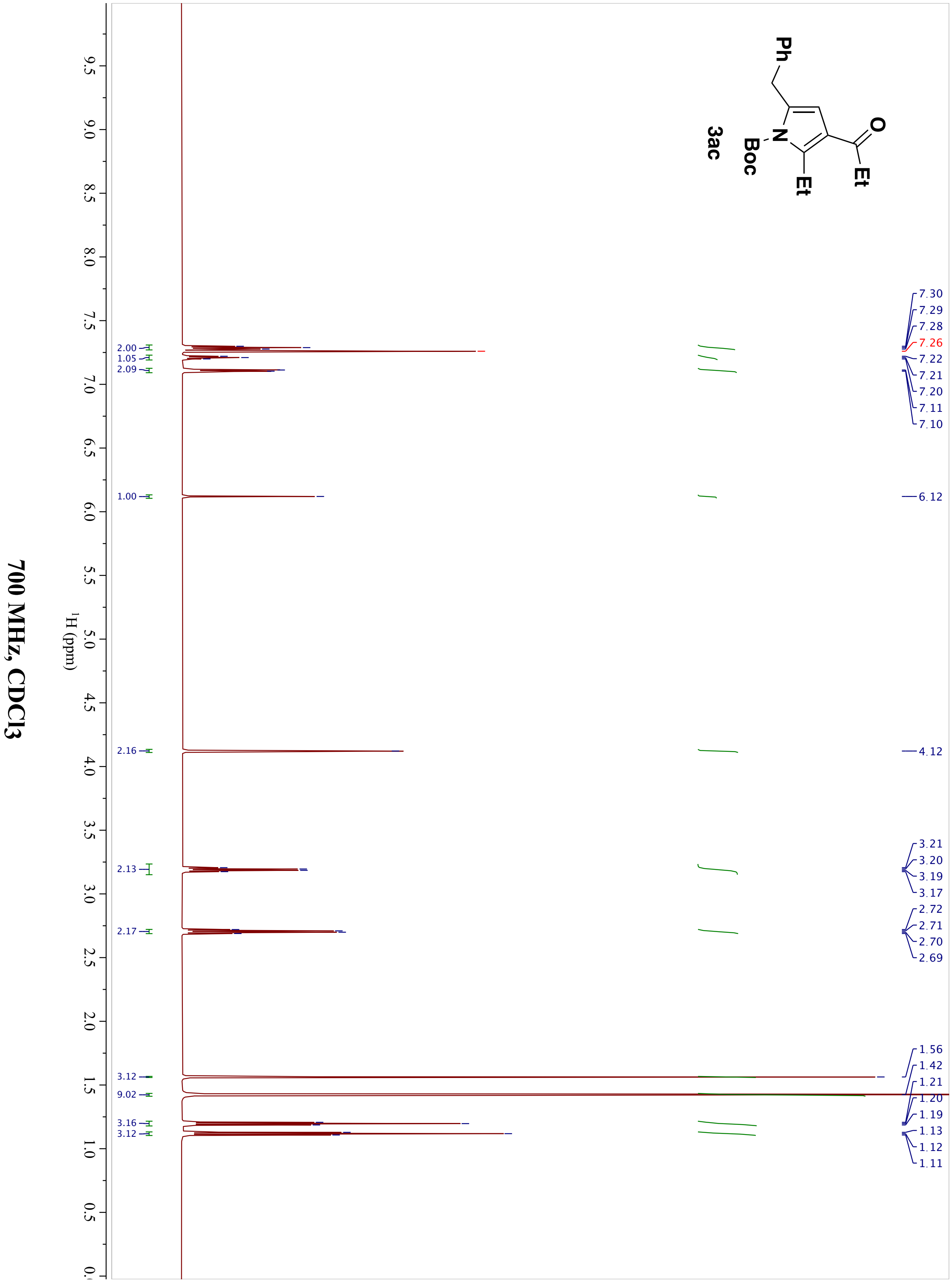
700 MHz, CDCl3
3ac
Ph
O
Et
N
Boc
Et
7.30
7.29
7.28
7.26
7.22
7.21
7.20
7.11
7.10
6.12
4.12
3.21
3.20
3.19
3.17
2.72
2.71
2.70
2.69
1.56
1.42
1.21
1.20
1.19
1.13
1.12
1.11
2.00
1.05
2.09
1.00
2.16
2.13
2.17
3.12
9.02
3.16
3.12
1H (ppm)

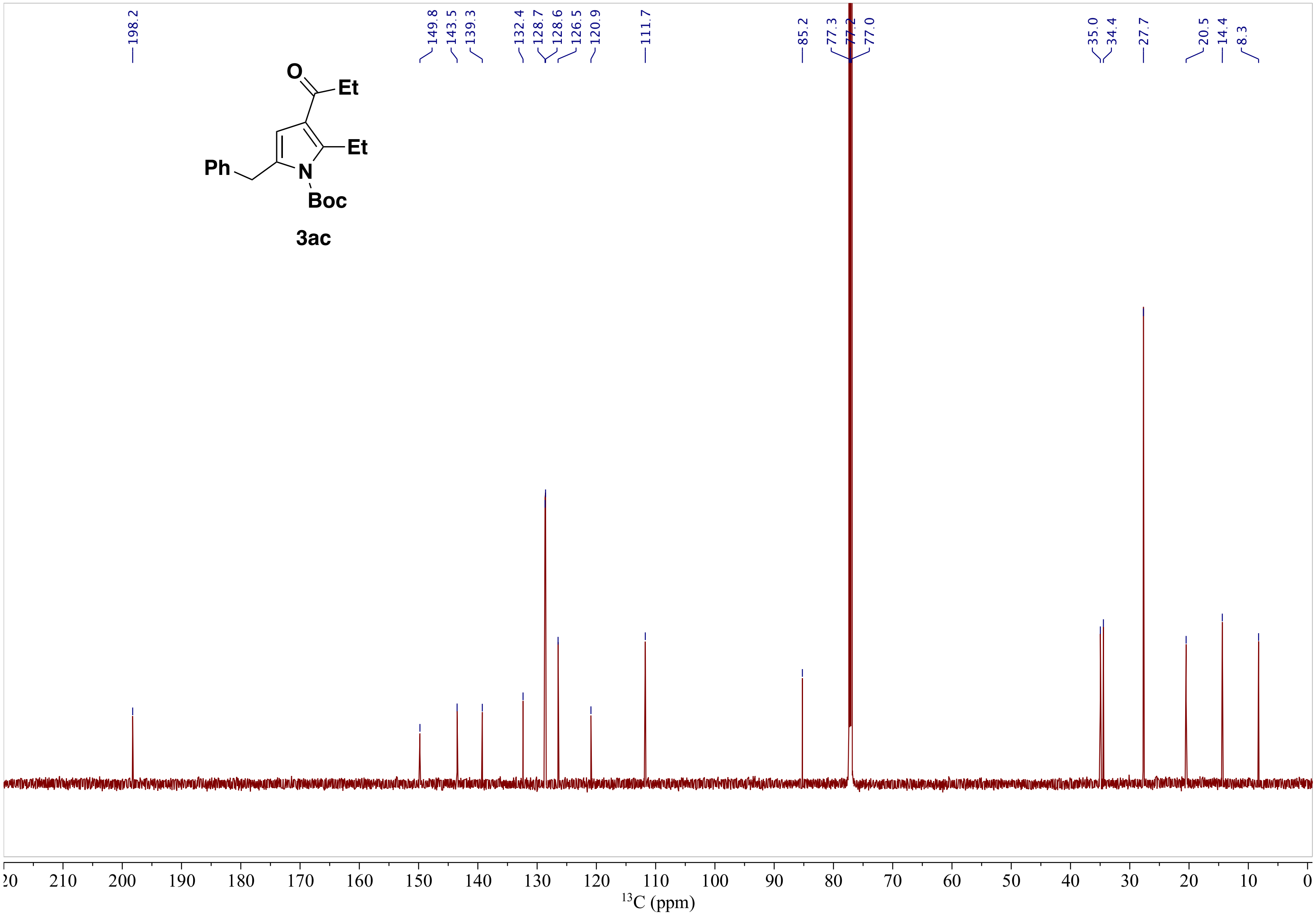

175 MHz, $CDCl_3$

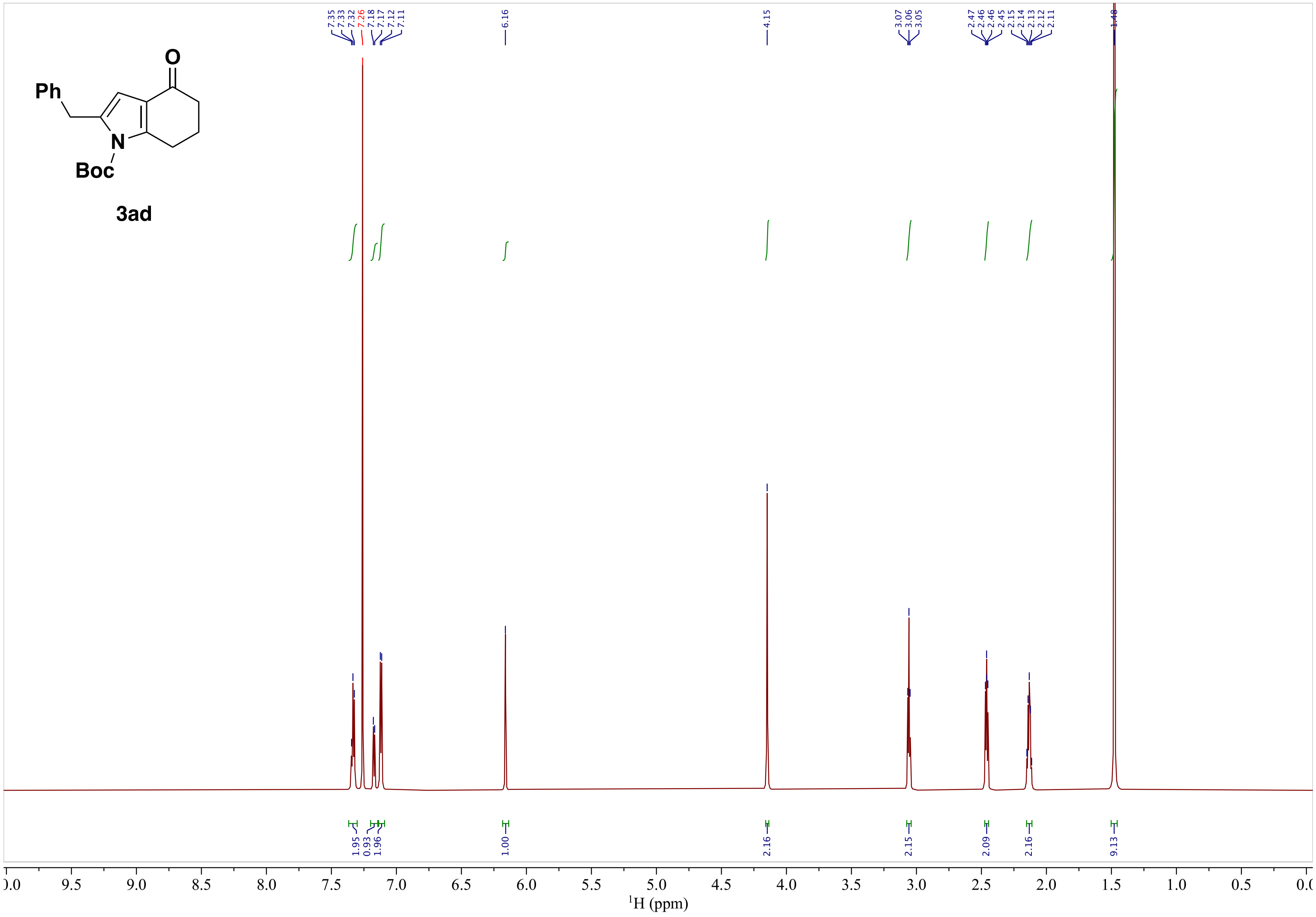

700 MHz, $CDCl_3$

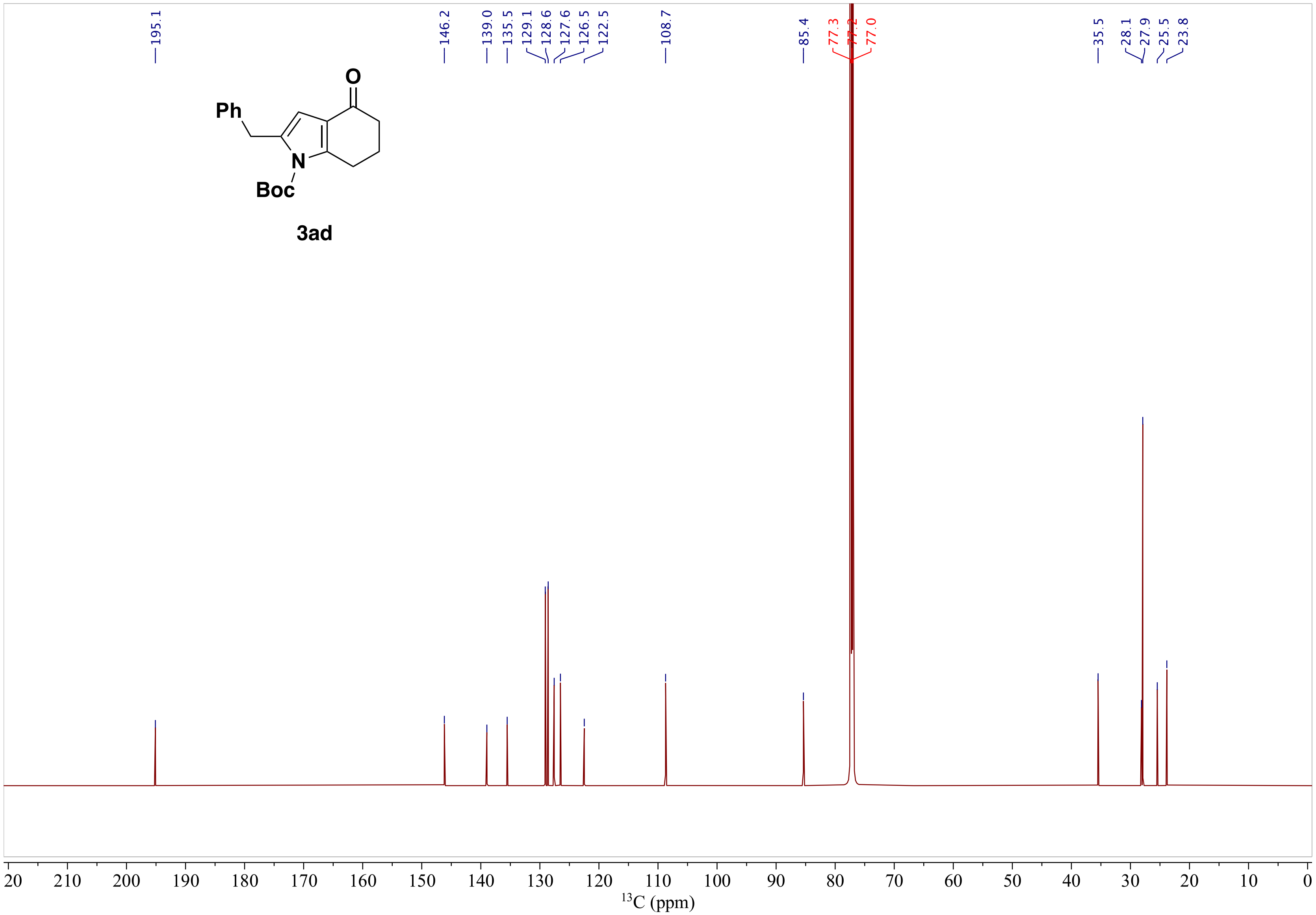

175 MHz, $CDCl_3$

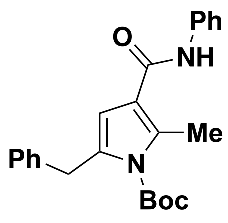

3ae

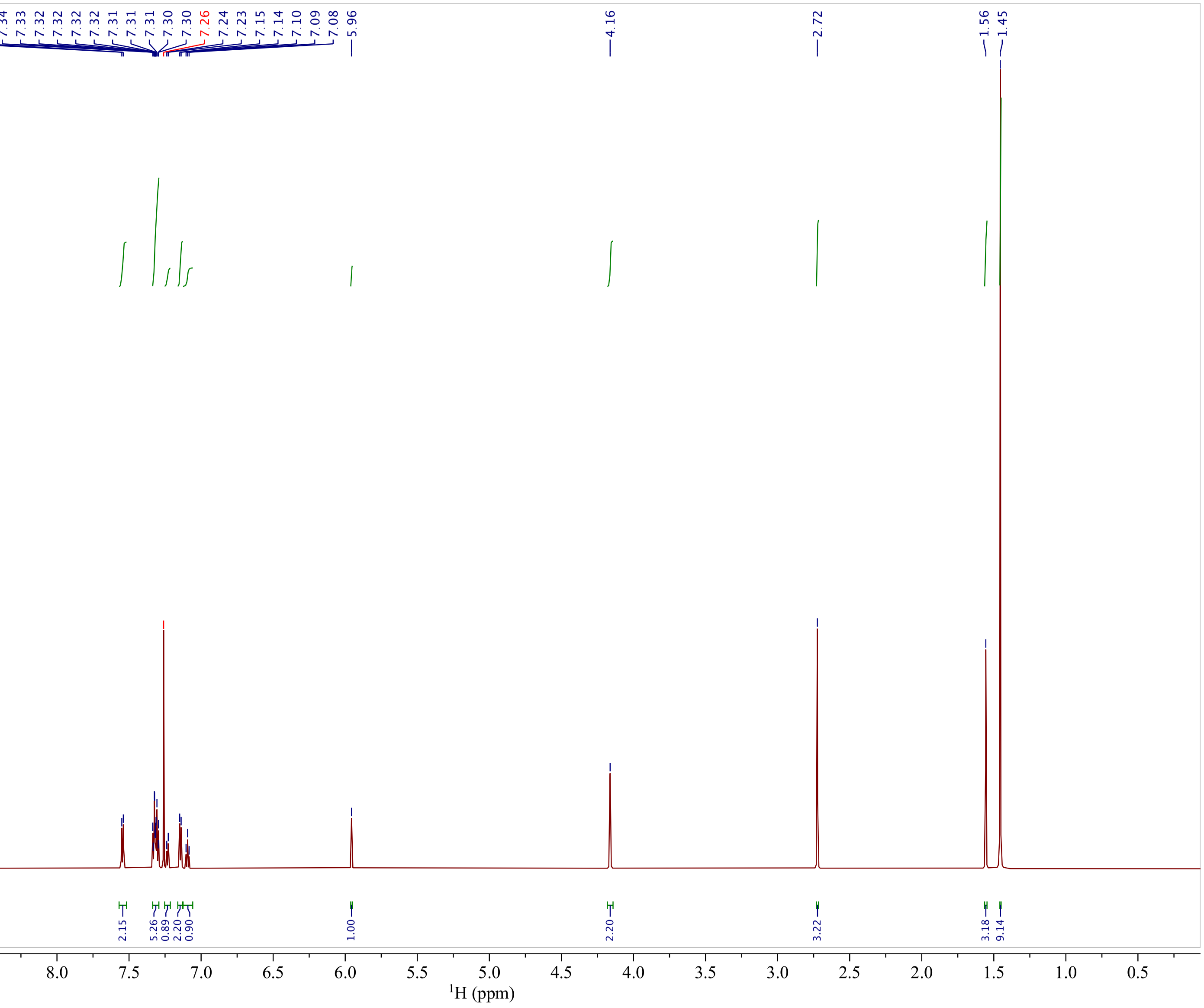

700 MHz, $CDCl_3$

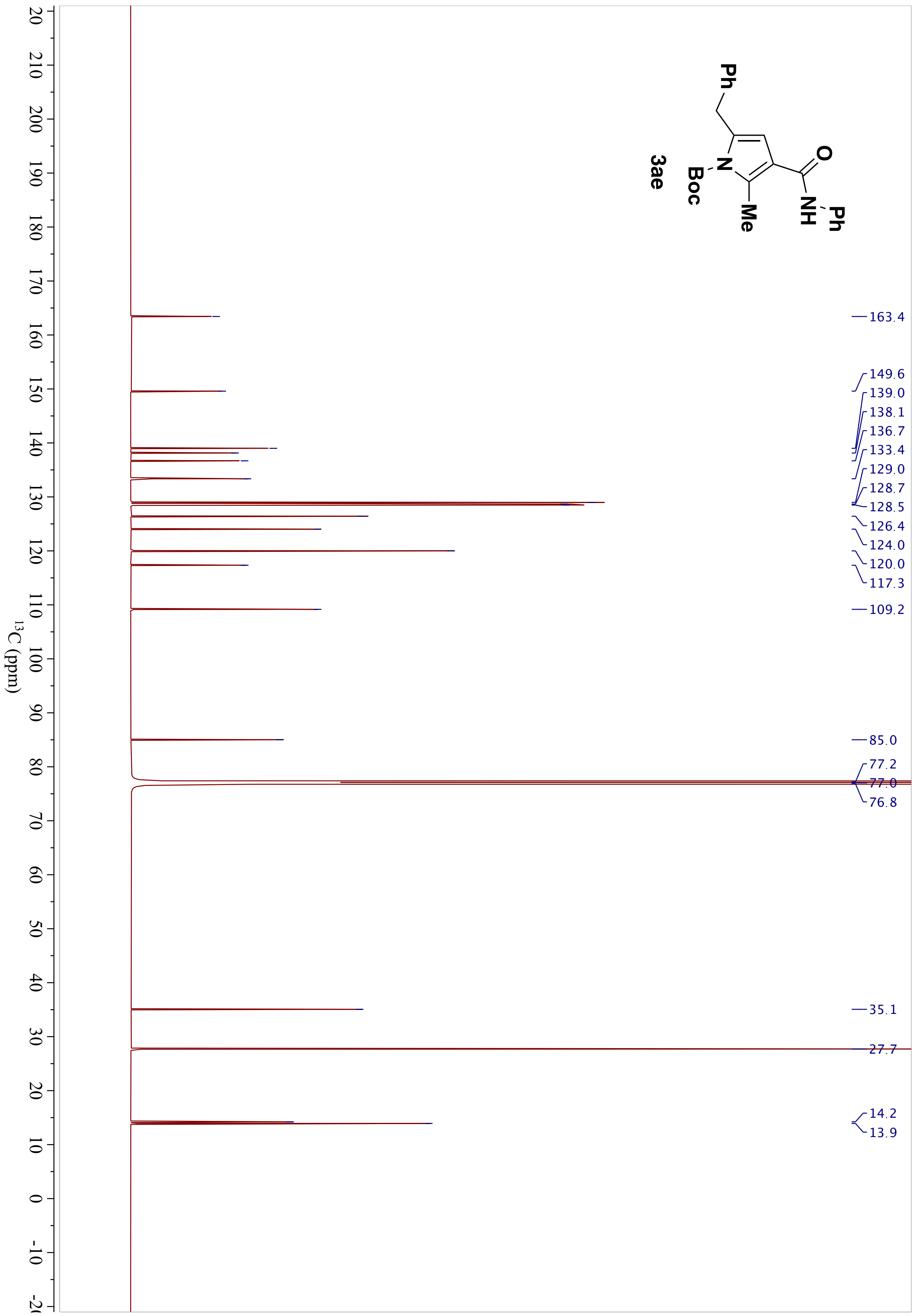

175 MHz, $CDCl_3$

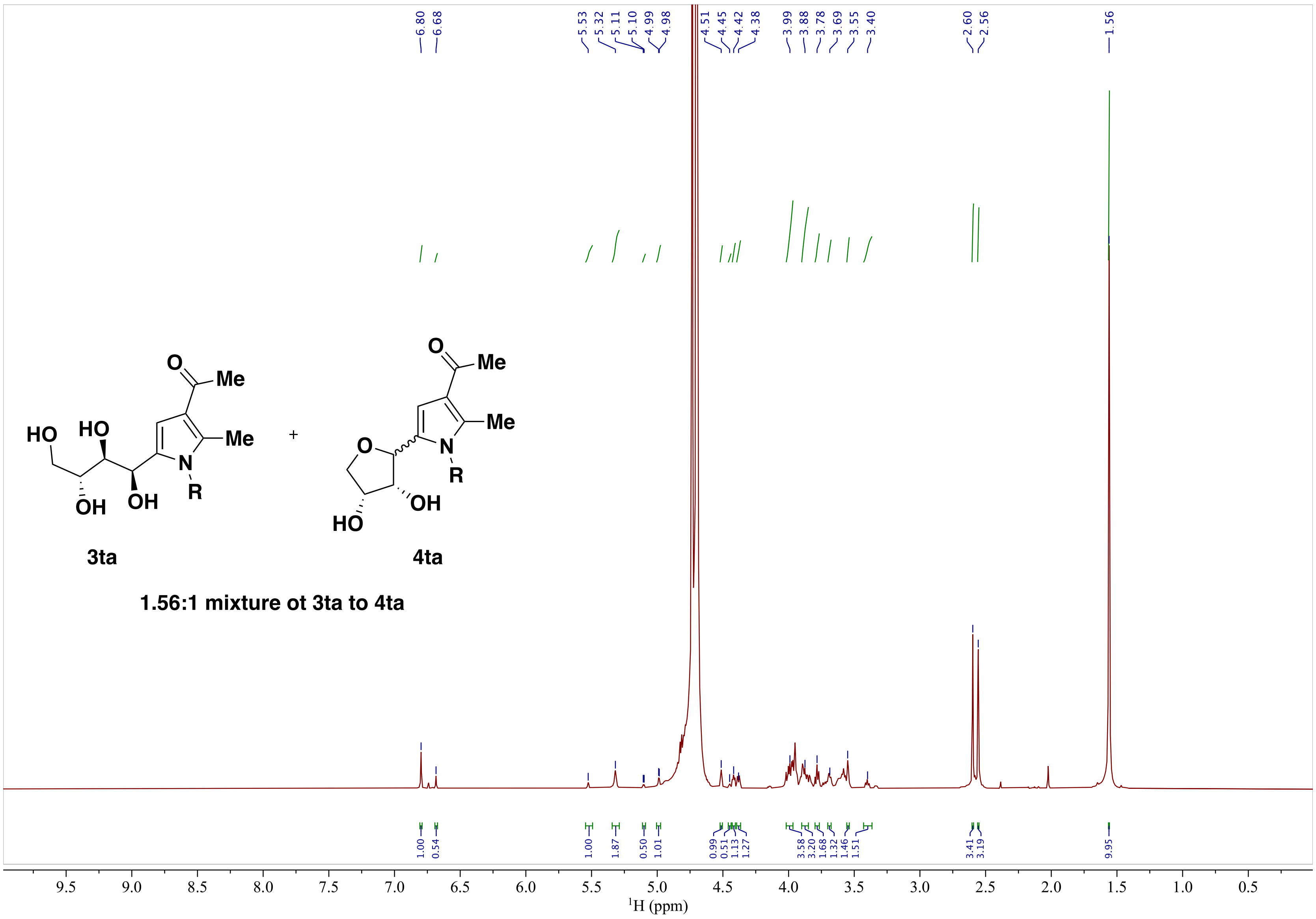
3ta
4ta
1.56:1 mixture ot 3ta to 4ta
6.80
6.68
5.53
5.32
5.11
5.10
4.99
4.98
4.51
4.45
4.42
4.38
3.99
3.88
3.78
3.69
3.55
3.40
2.60
2.56
1.56
1.00
0.54
1.00
1.87
0.50
1.01
0.99
0.51
1.13
1.27
3.58
3.20
1.68
1.32
1.46
1.51
3.41
3.19
9.95
9.5
9.0
8.5
8.0
7.5
7.0
6.5
6.0
5.5
5.0
4.5
4.0
3.5
3.0
2.5
2.0
1.5
1.0
0.5
^{1}H (ppm)

700 MHz, D_2O

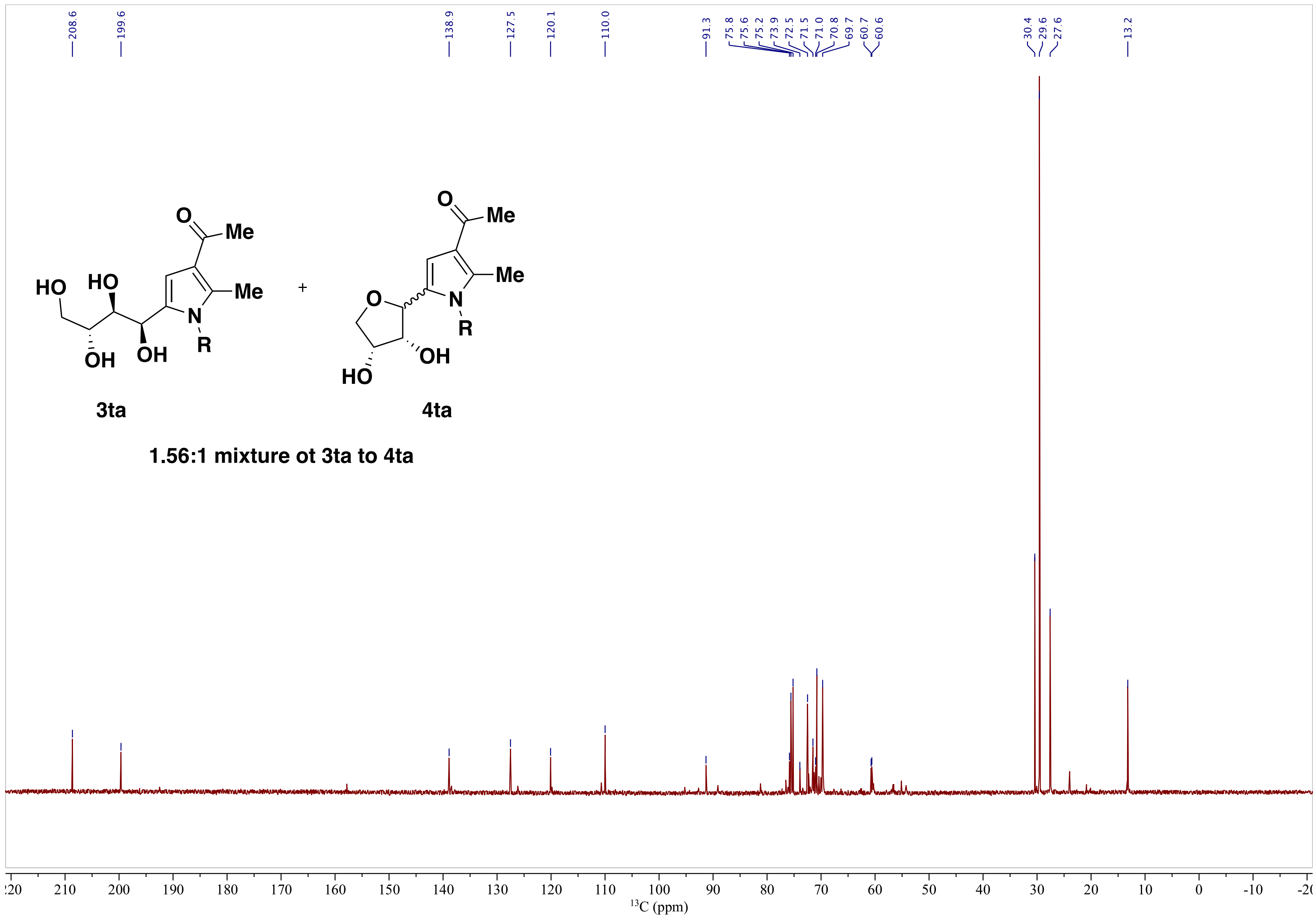

208.6
199.6
138.9
127.5
120.1
110.0
91.3
75.8
75.6
75.2
73.9
72.5
71.5
71.0
70.8
69.7
60.7
60.6
30.4
29.6
27.6
13.2
HO
HO
OH
OH
N
R
Me
O
Me
3ta
+
O
N
R
Me
O
Me
OH
HO
4ta
1.56:1 mixture ot 3ta to 4ta
220 210 200 190 180 170 160 150 140 130 120 110 100 90 80 70 60 50 40 30 20 10 0 -10 -20
13C (ppm)

175 MHz, D_2O

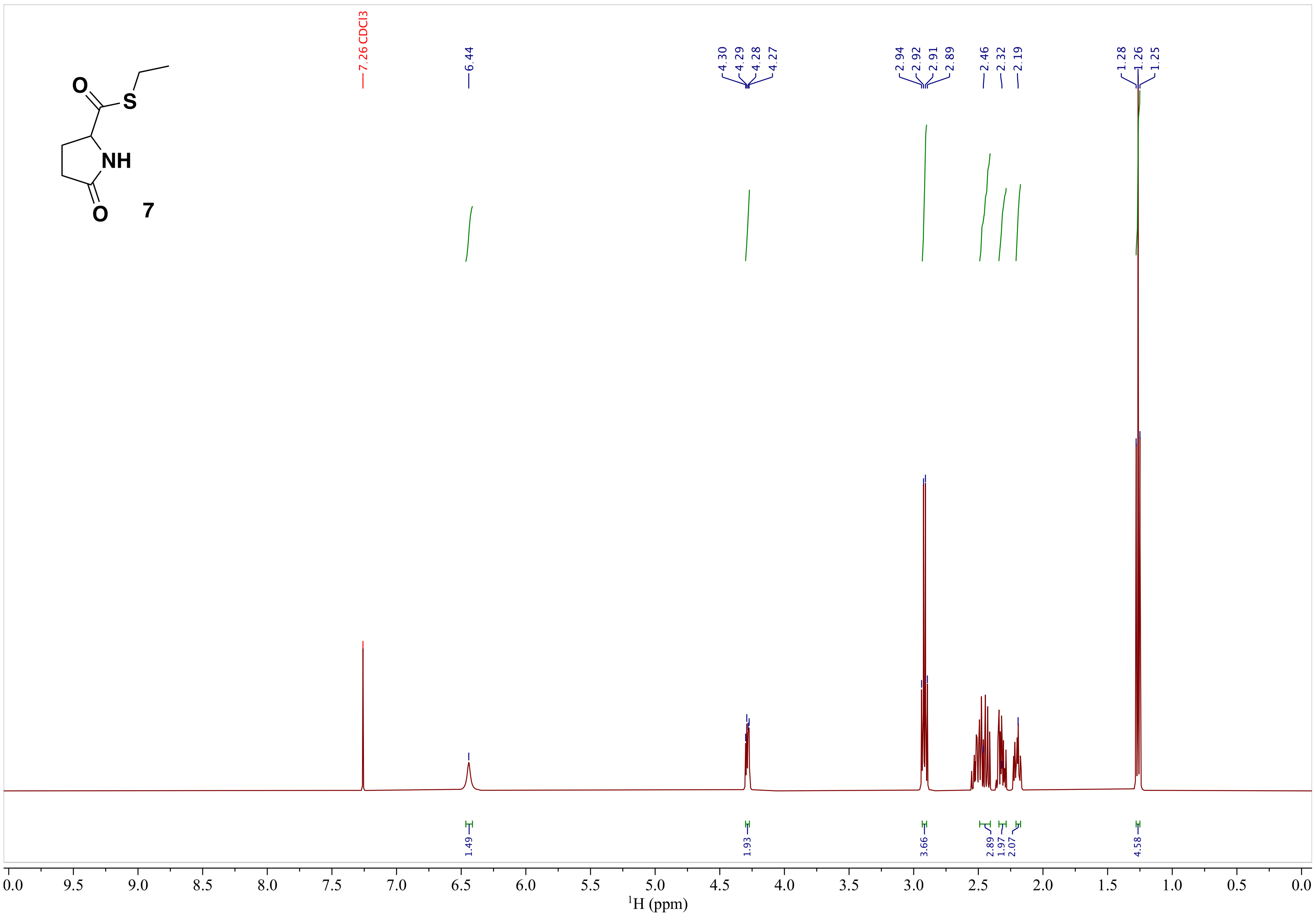

700 MHz, $CDCl_3$

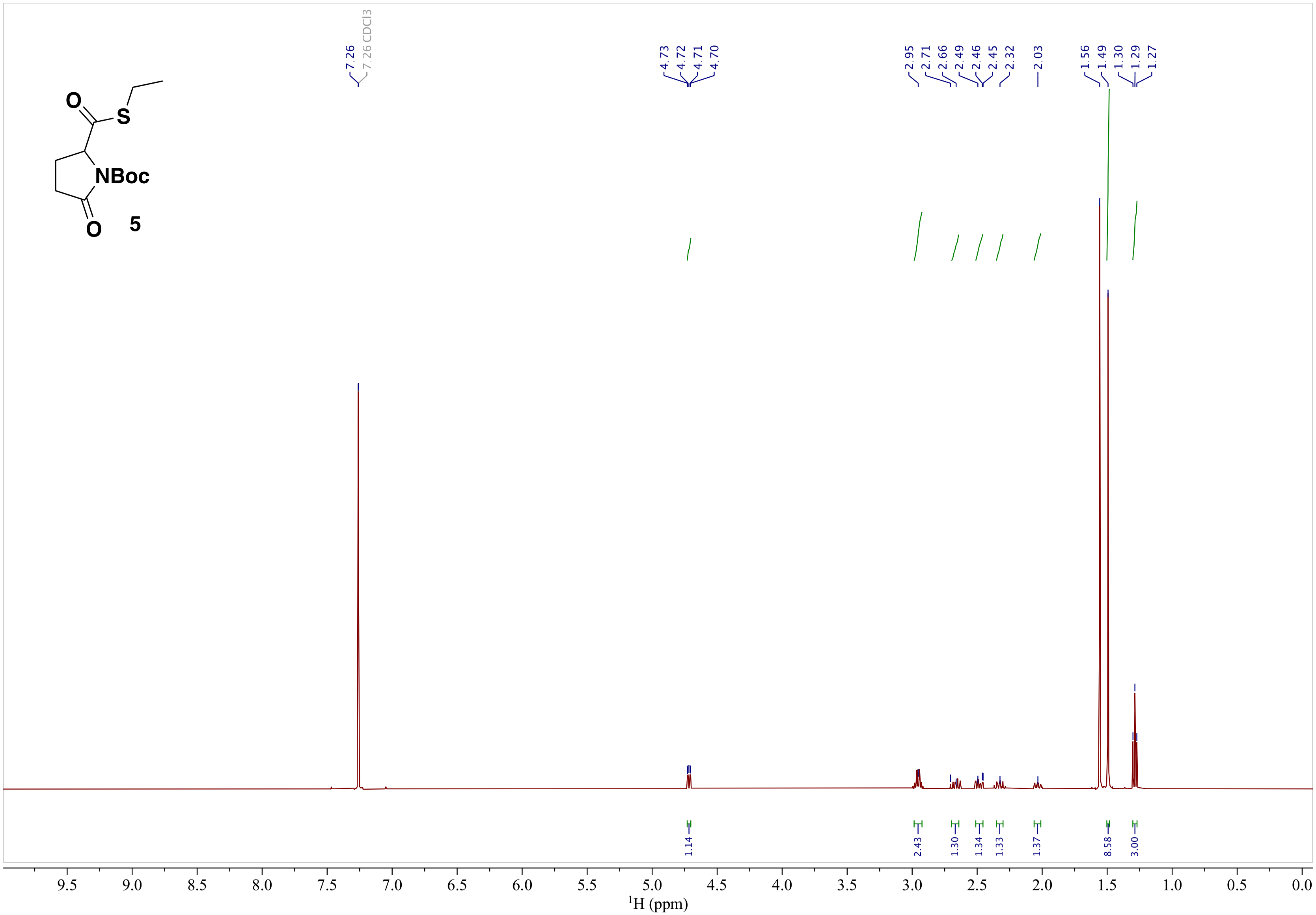

700 MHz, $CDCl_3$

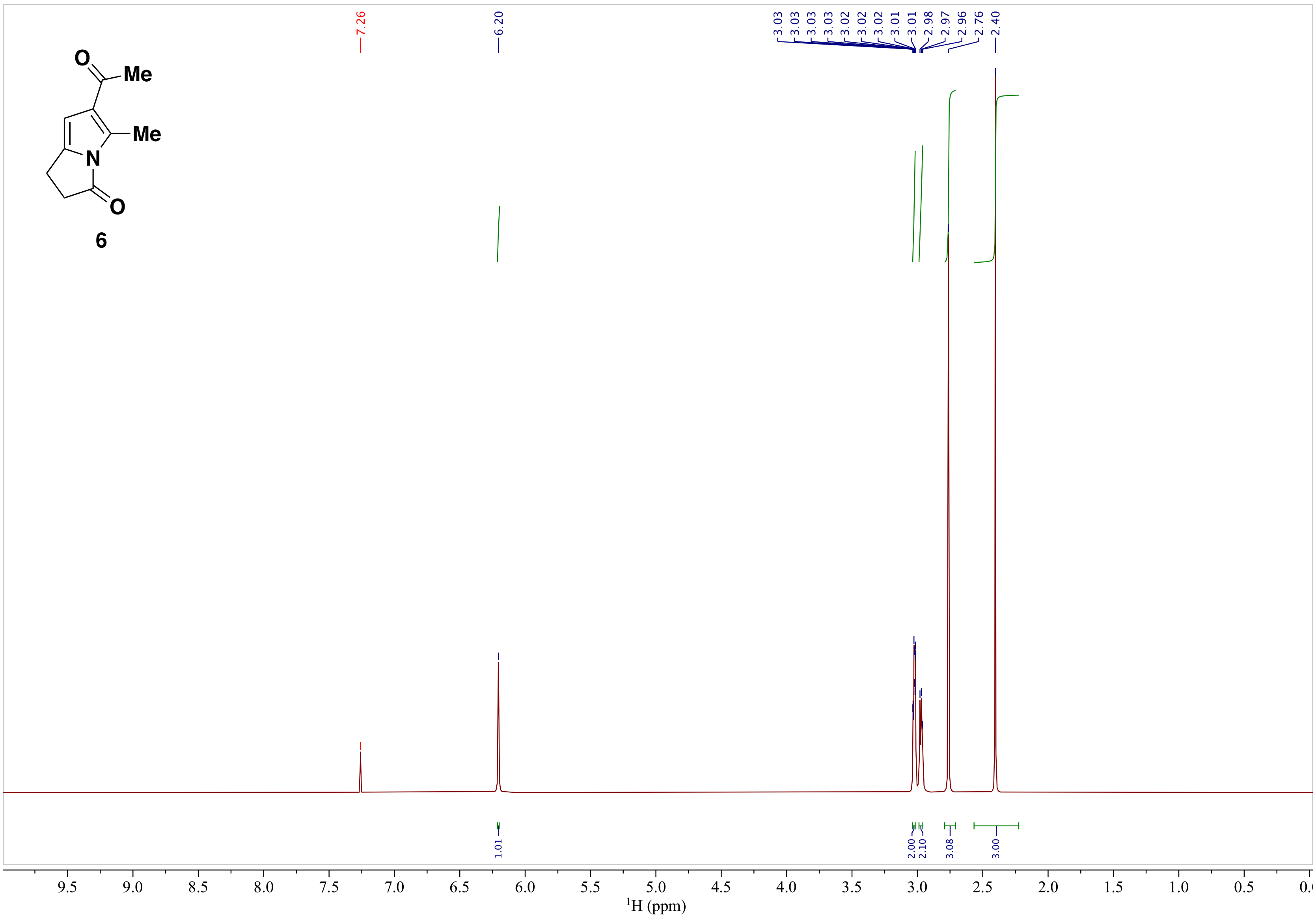

700 MHz, $CDCl_3$

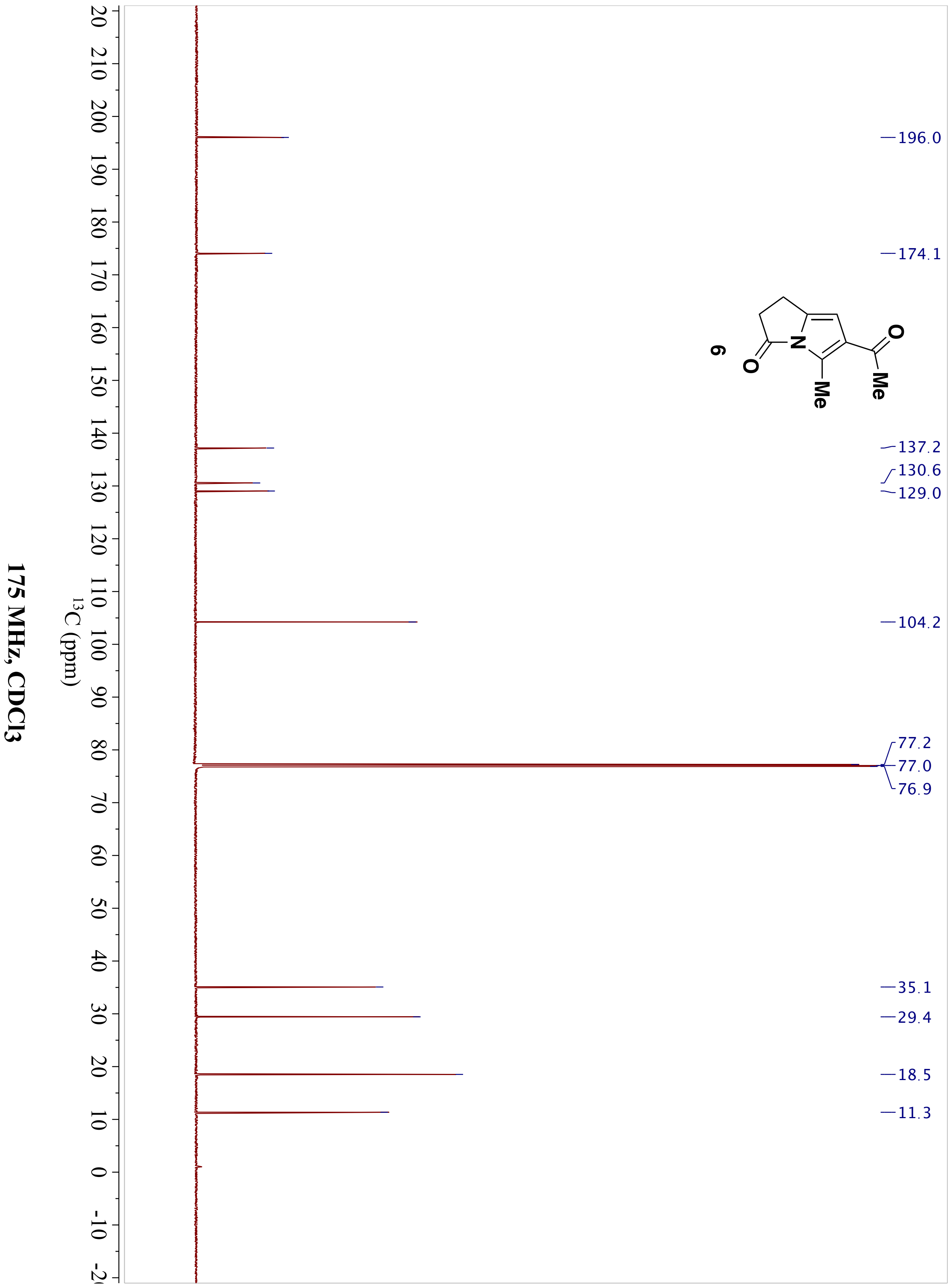

175 MHz, CDCl3
13C (ppm)
196.0
174.1
137.2
130.6
129.0
104.2
77.2
77.0
76.9
35.1
29.4
18.5
11.3
6
O
N
Me

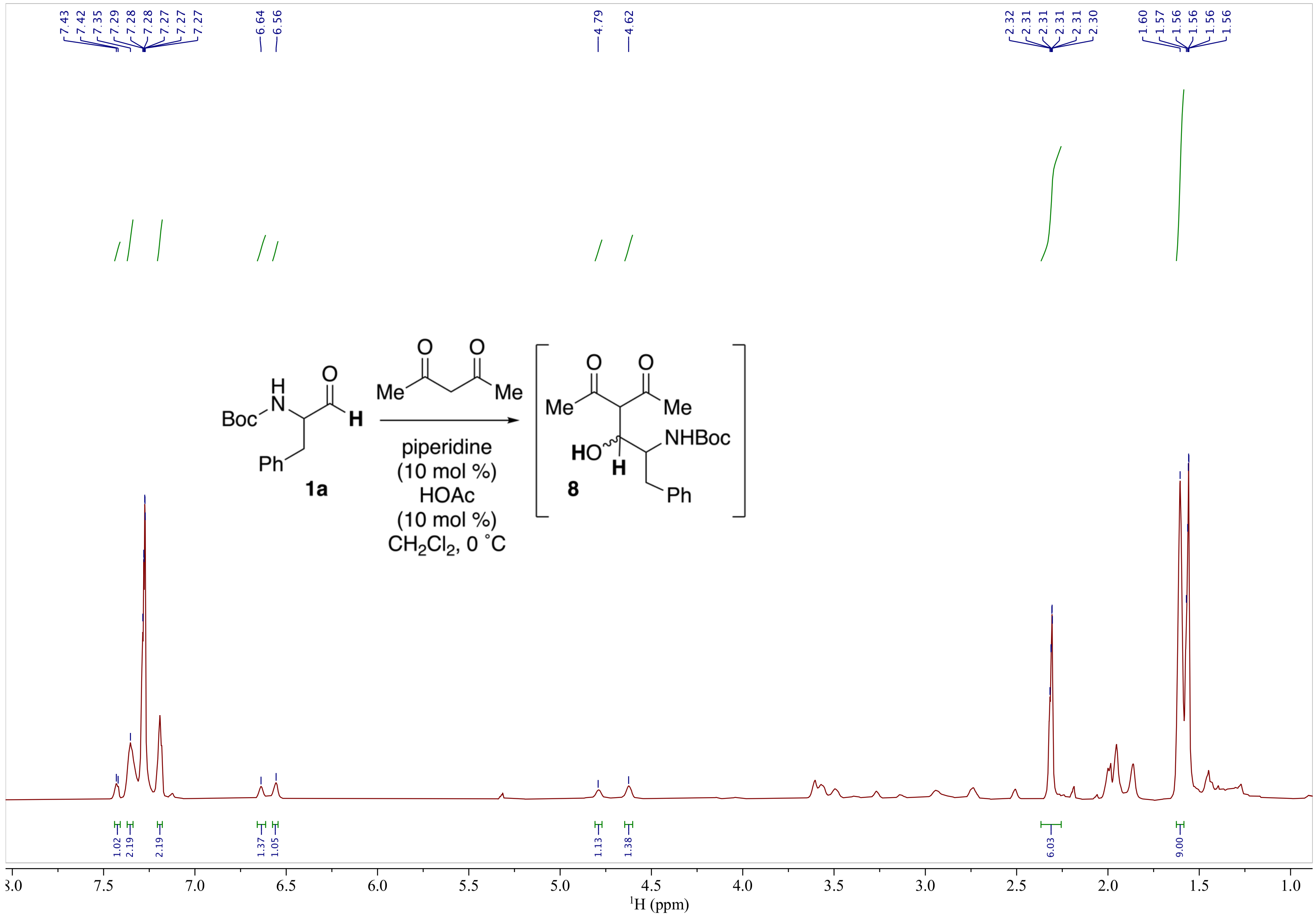

700 MHz, $CDCl_3$

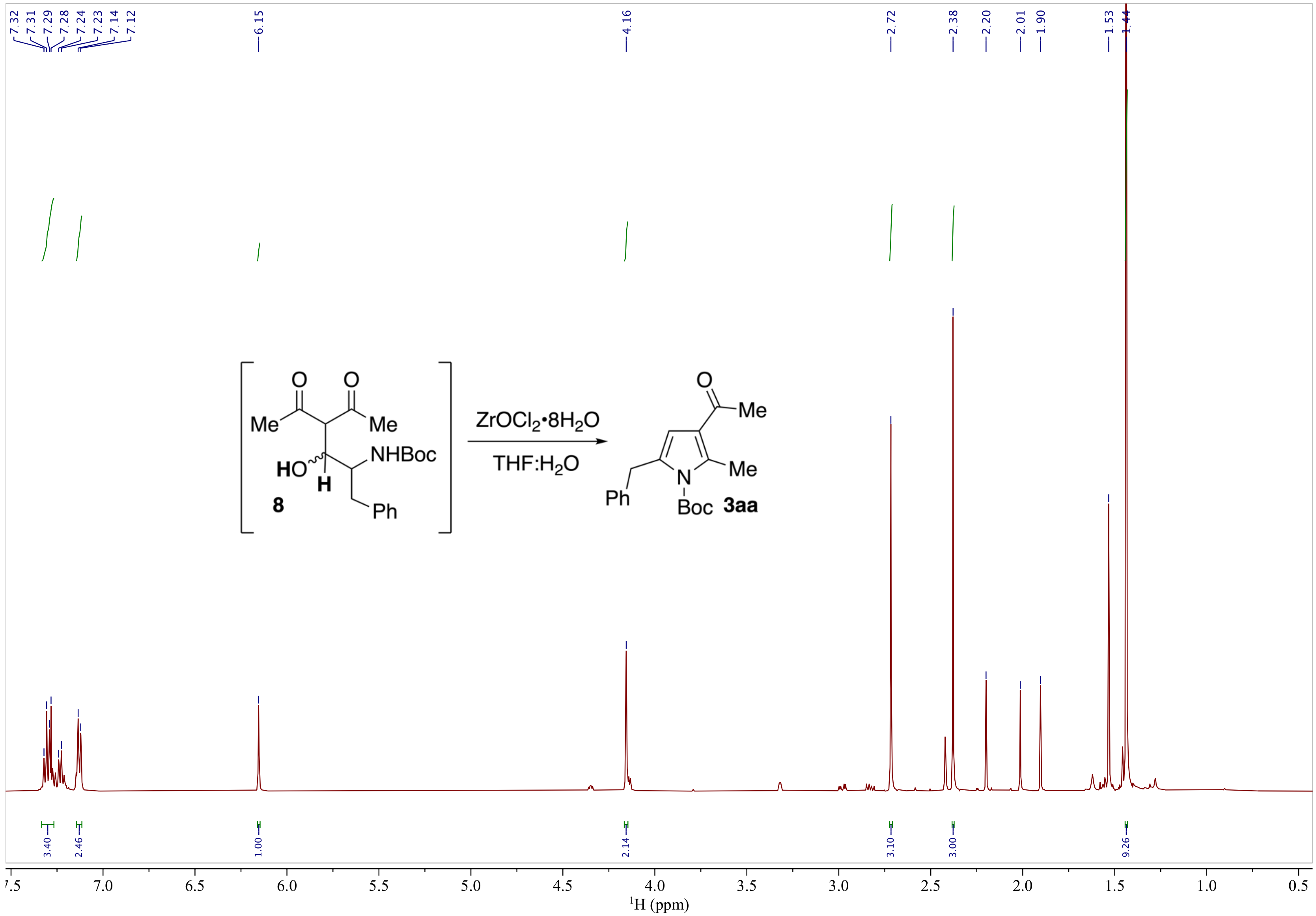

700 MHz, $CDCl_3$

3. HPLC Data for 3ra

Sample Name: *rac*-**3ra**
Column Info: RegisReflect C-Amylose A, 5μm, 250mm*4.6mm
Mobile Phase: 2%IPA/Hexanes
Flow Rate: 1.5 ml/min

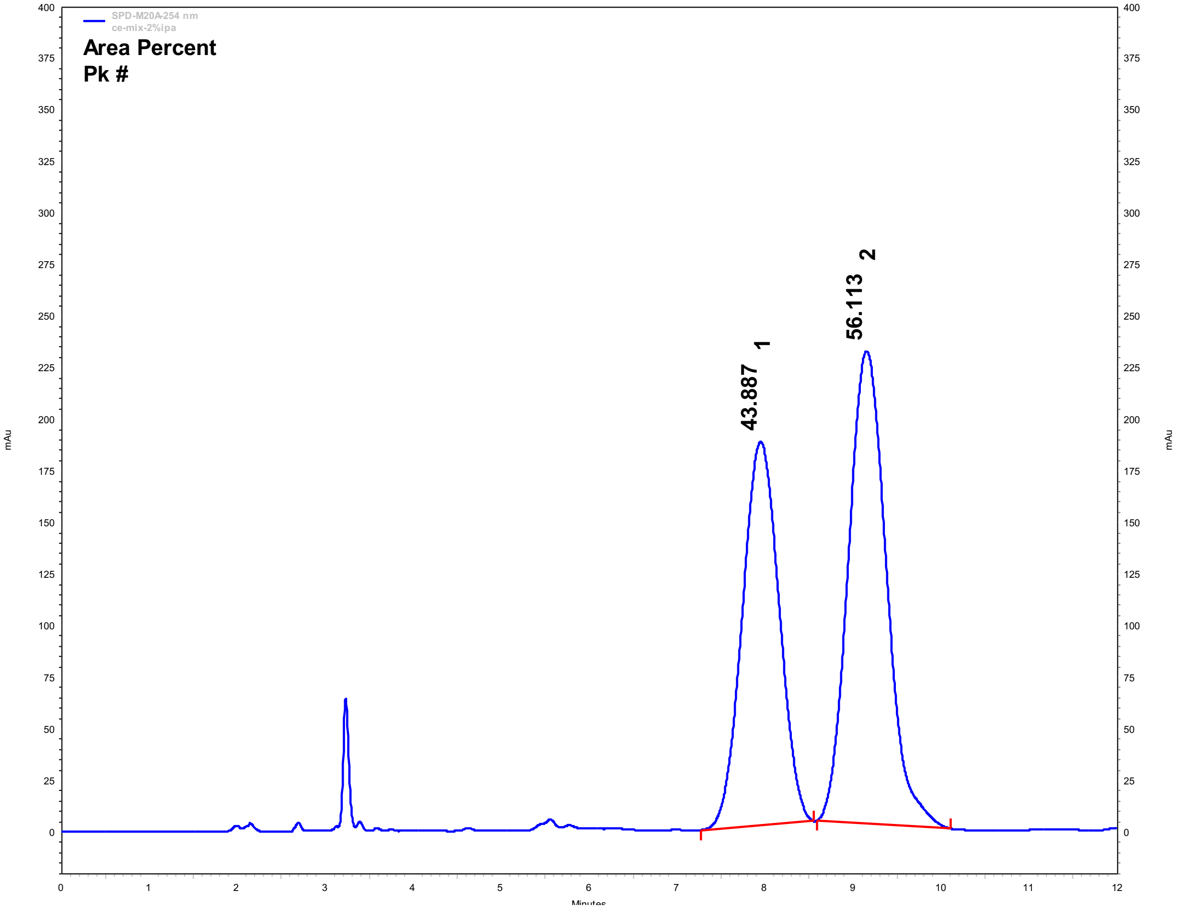

SPD-M20A-254 nm Results

Retention Time	Area	Area %	Height	Height %
7.956	5395284	43.89	185711	44.77
9.160	6898173	56.11	229059	55.23
Totals	12293457	100.00	414770	100.00

Sample Name: (*S*)-**3ra**
Column Info: RegisReflect C-Amylose A, 5μm, 250mm*4.6mm
Mobile Phase: 2%IPA/Hexanes
Flow Rate: 1.5 ml/min

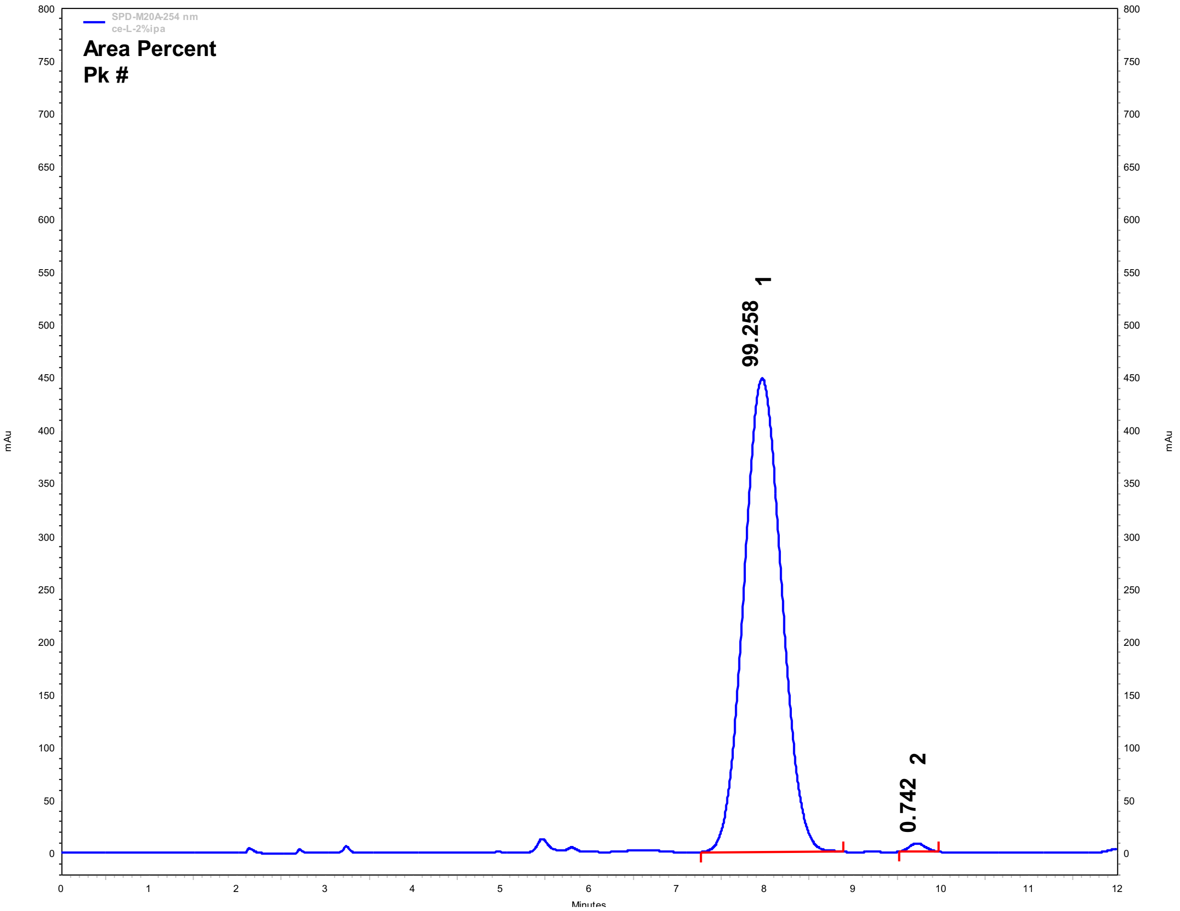

SPD-M20A-254 nm Results

Retention Time	Area	Area %	Height	Height %
7.972	13379965	99.26	448116	98.31
9.732	100007	0.74	7690	1.69

Totals				
	13479972	100.00	455806	100.00

Sample Name: (*R*)-**3ra**
Column Info: RegisReflect C-Amylose A, 5μm, 250mm*4.6mm
Mobile Phase: 2%IPA/Hexanes
Flow Rate: 1.5 ml/min

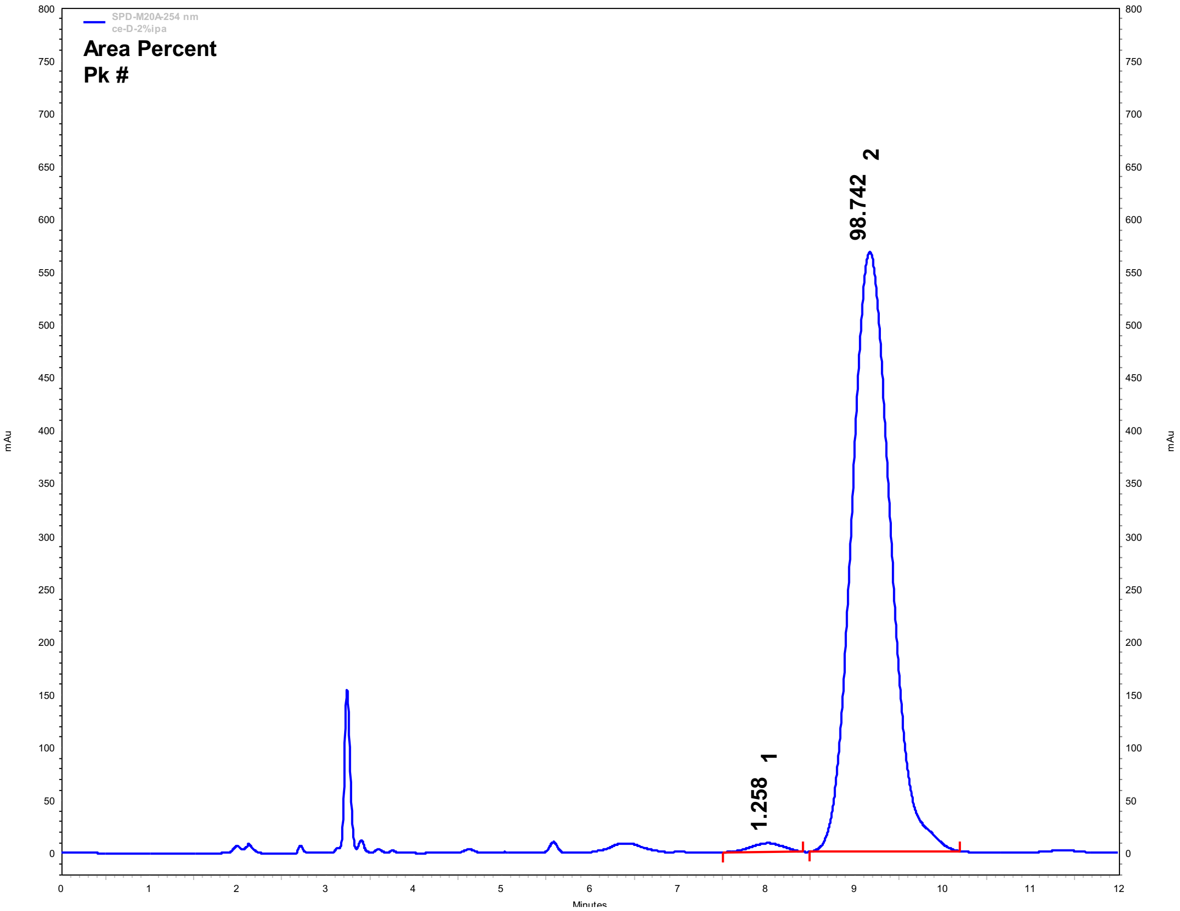

SPD-M20A-254 nm Results

Retention Time	Area	Area %	Height	Height %
8.028	221294	1.26	8499	1.48
9.180	17364645	98.74	567350	98.52
Totals	17585939	100.00	575849	100.00
